# Oral microbiota analysis of tongue coating in patients with esophageal adenocarcinoma

**DOI:** 10.1097/MD.0000000000045160

**Published:** 2025-10-10

**Authors:** Huijie Wang, Jinfeng Wang, Jinli Liu, Xiaoyang Shi, Zhichao Wang, Xu Cao

**Affiliations:** a Department of Endoscopy, Shijiazhuang Traditional Chinese Medicine Hospital, Shijiazhuang, China; b Department of Surgery, Shijiazhuang Traditional Chinese Medicine Hospital, Shijiazhuang, China.

**Keywords:** 16S rRNA, esophageal adenocarcinoma, metabolic pathway, microbiota, PICRUSt, tongue coating

## Abstract

The incidence of esophageal adenocarcinoma (EAC) has been steadily rising in China, emphasizing the need for effective strategies in primary prevention and early detection. Emerging evidence suggests that tongue coating microbiota may play a significant role in gastrointestinal diseases. However, the alterations in tongue coating microbiota and associated metabolic pathways in Chinese EAC patients remain poorly understood. This case-control study analyzed tongue coating microbiota samples from 28 EAC patients and 28 age- and sex-matched control subjects. Microbiota composition and predicted metabolic functions were assessed using PICRUSt2. Alpha diversity was measured using the Shannon and Chao1 indices, and microbial taxa were compared between groups using the Wilcoxon signed-rank test. The Shannon index was significantly higher in the EAC group (*P* = .033), suggesting greater microbial diversity, while no significant difference was found in the Chao1 index (*P* = .50). The EAC group exhibited significant enrichment of Porphyromonas and Tannerella, while Rothia was significantly reduced. Predicted metabolic pathways, including pyrimidine metabolism, amino acid metabolism, and carbon metabolism, showed notable differences between the groups. This exploratory study highlights distinct alterations in tongue coating microbiota and metabolic functions in EAC patients, offering potential insights for the development of noninvasive diagnostic approaches for EAC.

## 1. Introduction

In 2020, the morbidity and death rates of esophageal cancer (EC) were ranked seventh and sixth, respectively, globally,^[1]^ associated with high mortality, poor prognosis at diagnosis, and variation by geographical location.^[2]^ In China, the incidence of EC in 2020 was 13.80/100,000, ranking sixth, and the mortality rate was 12.70/100,000, ranking fourth.^[3]^ Based on the histological classification, the most common ECs are esophageal adenocarcinoma (EAC) and esophageal squamous cell carcinoma (ESCC). Although the main type of histological in China is ESCC, the incidence of EAC has also risen continually.^[4–9]^

Recent research found that alterations in the composition and functions of the oral microbiota from the United States population are associated with Barrett esophagus (BE), a precancerous lesion of EAC, and EAC itself. As the primary precursor lesion of EAC, BE not only reshapes the local microbiota, but also promotes the displacement of oral microorganisms to the esophageal mucosa. However, the relationship between oral microbiota and EAC remains unclear^[10,11]^; hence, further investigate the relationship is needed.

The occurrence of ecological imbalance is related to the host metabolic response that regulates cancer progression.^[12]^ Research suggests that metabolite production and catabolism associated with microbiota activity drive the pathology of many common human diseases due to dysbiosis.^[13–15]^ The ecological imbalance of the oral microbiota may cause cancer through different mechanisms. Two possible common mechanisms are chronic inflammation and the synthesis of metabolites that may induce mutations.^[16]^ Exploring how specific microbiota and metabolites contribute to human health is a critical area of research.^[17,18]^ Researchers have demonstrated that specific microbiota and metabolites can be used to diagnose or predict disease.^[19]^ Thus, researchers expect that in the next decade, predictive models built based on microbiota or metabolite characteristics will become noninvasive clinical tools.^[19]^

The oral cavity is an open system with a complex structure and microbial environment.^[20]^ Different sample types are used to study the oral microbiome, and each sample has its own advantages and limitations. Saliva, often used for its noninvasive collection and representation of whole-oral-community bacteria, suffers from high variability due to flow rate, diet, and time-of-day effects,^[21]^ potentially diluting niche-specific signals. Recent studies have shown that the salivary microbiome does not represent the entire oral microbiome,^[22,23]^ and is significantly less representative than the microbiome of the supragingival plaque.^[24]^ The tooth surface is the only nonshedding surface in the oral cavity and therefore provides an ideal environment for bacterial growth and the formation of dental plaque. Studies have shown that dental plaque has higher α-diversity, microbial richness and homogeneity than saliva and tongue samples.^[22]^ However, its composition is heavily influenced by oral hygiene practices and periodontal status, introducing confounding variables.^[25]^ The structural characteristics of the epithelial layer of the oral mucosa mean that it is constantly shed. Although it remains continuously colonized by oral microorganisms, these microorganisms are relatively restricted.^[26]^ Interestingly, the tongue has a higher density and more microbial diversity than other mucosal surfaces.^[27]^ At the same time, traditional Chinese medicine regards the tongue as an important indicator to identify health conditions, known as tongue diagnosis.^[28]^ The microbiota is an important component of the tongue. The rich microbiota on the tongue reflects individual characteristics and disease status.^[5,29,30]^

Considering the increasing incidence, high fatality rate, mostly progressive, and poor prognosis at diagnosis of EAC in China over the years, there is an urgent need to achieve primary prevention and early detection of EAC. This study provides a theoretical basis for the future development of noninvasive detection tools by comparing the changes in tongue coating microbiota and predicted metabolic functions between patients with EC and healthy controls.

## 2. Materials and methods

### 2.1. Study design and participants

Tongue coating samples were collected from 2 groups, consisting of 28 patients with EAC and 28 healthy individuals, in this case-control study. The subjects were recruited from patients presenting to Shijiazhuang Traditional Chinese Medicine Hospital from March 2020 to February 2023. Demographic information, clinical data, medication history, smoking, and drinking status were collected.

The study was approved by the Ethics Committee of Shijiazhuang Traditional Chinese Medicine Hospital in 2018 and was conducted by the Helsinki Declaration. All subjects provided written informed consent before the collection of samples.

The diagnostic process of EAC involved endoscopic examination, where a small amount of mucosa was taken from the lesion site for histopathological evaluation based on the WHO Classification of Tumors 2019^[31]^ for suspected EAC patients.

#### 2.1.1. Inclusion criteria

All subjects were from the Han population residing in permanent urban areas of Shijiazhuang. Patients with EAC were included if they were first diagnosed. The control group consisted of healthy individuals matched for age (±5 years) and sex during the same period.

#### 2.1.2. Exclusion criteria

Subjects were excluded if they had undergone any antineoplastic treatment or upper gastrointestinal surgery, had active oral infection, other malignancies, used antibiotics within 6 months before sampling, or were using proton pump inhibitors or other drugs for gastrointestinal disorders.

### 2.2. Sample collection

The patient fasted (≥8 hours) before the endoscopy without brushing their teeth and tongue coating. Also, patients were asked to gargle 3 times (10 mL each time) with sterile water before sampling. A sterile disposable cotton swab was used to wipe 3 times from the middle of the tongue coating (about 2 cm long wiping motion) and put in sterile cryopreservation tubes. The samples were then rapidly frozen in liquid nitrogen and stored at −80°C until analysis.

### 2.3. 16S rRNA gene analysis

Genomic DNA was extracted using the EZNA^®^ soil DNA Kit (Omega Bio-Tek, Norcross). Primer pairs 338F (5’-ACTCCTACGGGAGGCAGCAG-3’) and 806R (5’-GGACTACHVGGTWTCTAAT-3’) were used on an ABI GeneAmp^®^ 9700 PCR thermocycler (Applied Biosystems, Foster City) to amplify the V3-V4 hypervariable region of the bacterial 16S rRNA gene. The PCR amplification reactions were performed in triplicate, involving an initial denaturation at 95°C for 3 minutes, followed by 27 cycles of denaturation at 95°C for 30 seconds, annealing at 55°C for 30 seconds, and extension at 72°C for 45 seconds. A final extension step was performed at 72°C for 10 minutes, followed by holding at 4°C. Subsequently, PCR products were extracted from 2% agarose gels and purified with the AxyPrep DNA Gel Extraction Kit (Axygen Biosciences, Union City). The purified DNA was quantified using a Quantus™ Fluorometer (Promega, Madison). Amplicons were pooled at equimolar concentrations and paired-end sequenced on the Illumina MiSeq PE300 platform (Illumina, San Diego) using standard protocols by Majorbio Bio-Pharm Technology Co., Ltd (Shanghai, China). The raw sequencing reads were deposited into the NCBI Sequence Read Archive (SRA) database (Accession Number: PRJNA974387). Quality control and splicing of raw sequenced sequences using fastp version 0.20.0^[32]^ and FLASH version 1.2.7.^[33]^ UPARSE version 7.1^[34,35]^ was used to cluster OTUs at 97% similarity and to identify and remove chimeric sequences. Taxonomic analysis of representative sequences for each OTU was performed with RDP Classifier version 2.13 against a 16S rRNA database (Silva v138) with a confidence threshold of 0.7.^[36]^ The Shannon and Chao1 indices in the α-diversity analysis were assessed using Mothur software (version v.1.30.2). To minimize the potential impact of sequencing depth on α-and β-diversity measures, the number of 16S rRNA gene sequences from each sample was rarefied to 26,687. A phylogenetic tree was constructed using the maximum likelihood method, followed by the generation of a Bray–Curtis distance matrix through FastUniFrac analysis. Subsequently, principal coordinates analysis (PCoA) analysis and visualization were carried out based on this distance matrix using R (version 3.3.1), and an ANOSIM analysis was performed using the vegan package in R. Metagenomic function prediction was conducted using PICRUSt2 (Phylogenetic Investigation of Communities by Reconstruction of Unobserved States)^[37]^ based on OTU representative sequences, with the identified features mapped to the Kyoto Encyclopedia of Genes and Genomes (KEGG) pathway.

### 2.4. Statistical analysis

All analyses were conducted by the stats package (version 4.2.1) in R.

Baseline data, including continuous or categorical variables, was analyzed by paired Student *t* test or McNemar test, respectively. *P* < .05 indicated statistical significance (two-tailed).

Differences between groups for the Shannon and Chao1 indices was performed by the paired Student *t* test. The Wilcoxon signed-rank test was conducted to explore differential genera as well as differential pathways derived from PICRUSt2. *P*-values were adjusted by false discovery rate. An adjusted *P*-value of <.05 was considered statistically significant.

## 3. Results

### 3.1. Baseline characteristics of participants

There were 28 patients with EAC and 28 controls, and the age and sex of participants were matched in this study. The baseline characteristics are presented in Table 1. The average age was 66.8 ± 8.9 years in the EAC group and 66.3 ± 8.7 years in controls. No statistical differences were detected in smoking and drinking status between the EAC and controls. No participants withdrew, and all tongue coating samples were eligible for analysis.

**Table 1:** Baseline characteristics of participants.

Variables	Case n = 28	Control n = 28	*P*-value
Age, yr, Mean ± SD	66.8 ± 8.9	66.3 ± 8.7	.066
Sex (%)			
Female	6 (21.4)	6 (21.4)	1
Male	22 (78.6)	22 (78.6)	
Smoking status (%)			
No	18 (64.3)	20 (71.4)	.563
Yes	10 (35.7)	8 (28.6)	
Drinking status (%)			
No	17 (60.7)	22 (78.6)	.131
Yes	11 (39.3)	6 (21.4)	

Data are presented as the mean ± SD or N (%).

### 3.2. Diversity of the tongue coating microbiota in patients with EAC and controls

**Figure 1.**
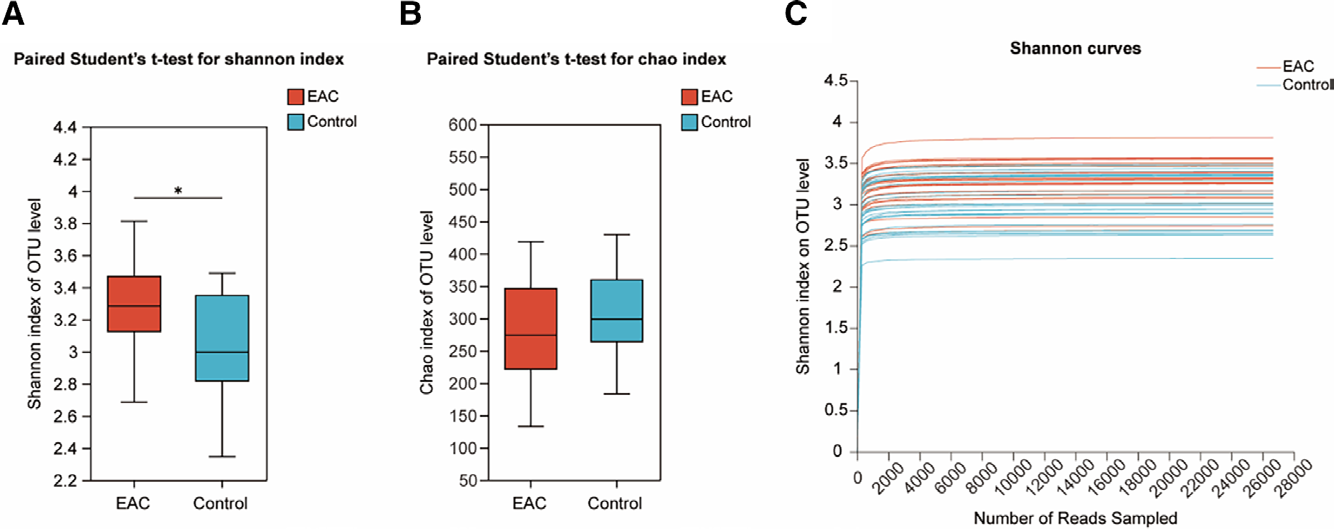
Comparison of α-diversity index of the tongue coating microbiota between the EAC and control groups (* *P* < .05). (A) Boxplots of Shannon index via the paired Student *t* test, *P* = .033; (B) Boxplots of Chao1 index in groups via the paired Student *t* test, *P* = .50; (C) Shannon curves. EAC = esophageal adenocarcinoma.

**Figure 2.**
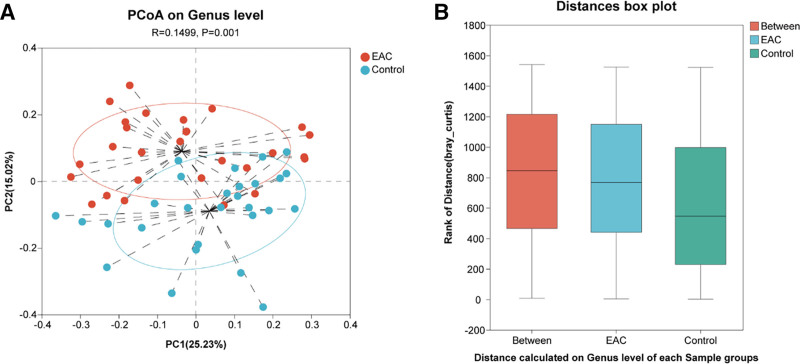
Comparison of the composition of the tongue coating microbiota between the EAC and control groups. There were significant differences in β-diversity (*P* = .001). (A) PCoA plots are based on Bray–Curtis distance. (B) Boxplots of within- and between-group Bray–Curtis distances (ANOSIM analysis). ANOSIM = analysis of similarities, EAC = esophageal adenocarcinoma, PCoA, principal coordinates analysis.

The Shannon (*P* = .033) and Chao1 (*P* = .50) indices were analyzed between the 2 groups. As shown in Figure 1, the Shannon index (Fig. 1A) is significantly higher for the EAC group than the control group. However, no significant difference is observed in the Chao1 index (Fig. 1B). This result indicated that there is no significant difference in the species richness of tongue coating microbiota, while differences exist in the evenness of species distribution. The Shannon dilution curve (Fig. 1C) shows that as sequencing depth increases, the curve tends to flatten, indicating sufficient sequencing depth. Furthermore, PCoA (Fig. 2A) and analysis of similarities (ANOSIM) based on Bray–Curtis distance (Fig. 2B) indicates that the composition and relative abundance of the tongue coating microbiota differed significantly between the EAC and controls (*P* = .001, *R* = 0.1499). Moreover, the alterations between groups were significantly greater than those within the group (*P = *.002, *R*^2^ = 0.092). The phylogenetic tree (Fig. S1, Supplemental Digital Content, https://links.lww.com/MD/Q333) reveals the evolutionary relationships of species at the genus level. Diversity analysis revealed significant differences in tongue coating microbiota diversity between EAC patients and controls.

### 3.3. Microbial differences in the tongue coating between patients with EAC and controls

**Figure 3.**
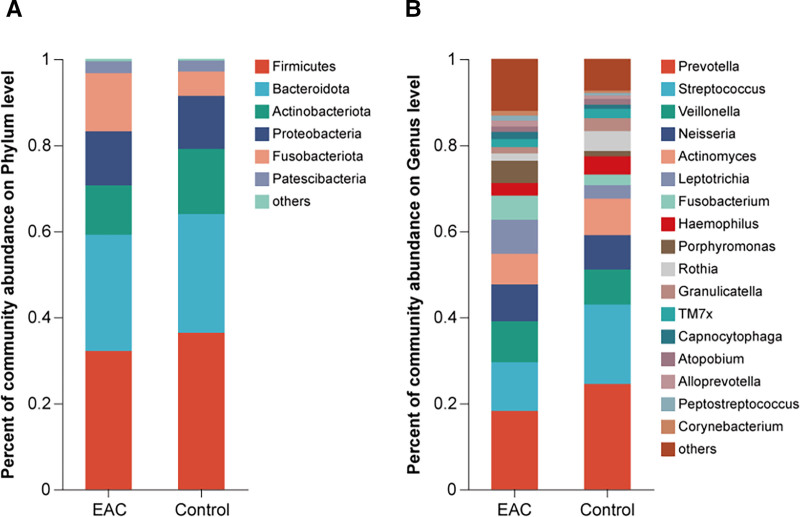
Comparison of the relative abundance of the tongue coating microbiota between the EAC and control groups. Identified 6 phylum and 17 genera in 2 groups. (A) Mean relative abundance at the phylum level. (B) Mean relative abundance at the genus level. EAC = esophageal adenocarcinoma.

The microbiota composition at the phylum level (relative abundance < 1% were classified as others) is shown in Figure 3A. Six main phyla were as follows: *Firmicutes* (EAC of 32.2% and Control of 36.4%), *Bacteroidota* (EAC of 27.0% and Control of 27.6%), *Proteobacteria* (EAC of 12.6% and Control of 12.3%), *Actinobacteriota* (EAC of 11.5% and Control of 15.1%), *Fusobacteriota* (EAC of 13.5% and Control of 5.6%), and *Patescibacteria* (EAC of 2.8% and Control of 2.5%). The microbial composition at the genus level (relative abundance < 1% were classified as others) is shown in Figure 3B. The main genera (EAC > 5%) were *Prevotella* (EAC 18.2% and Control 24.5%), *Streptococcus* (EAC 11.3% and Control 18.5%), *Veillonella* (EAC 9.5% and Control 8.1%), *Neisseria* (EAC 8.5% and Control 8.0%), *Leptotrichia* (EAC 7.9% and Control 3.1%), *Actinomyces* (EAC 7.2% and Control 8.5%), *Fusobacterium* (EAC 5.6% and Control 2.5%) and *Porphyromonas* (EAC 5.2% and Control 1.2%). There were commonalities and differences in the composition of the tongue microbiota in the EAC and control groups. We identified similar dominant genera in both groups, but differences in relative abundance rankings.

**Figure 4.**
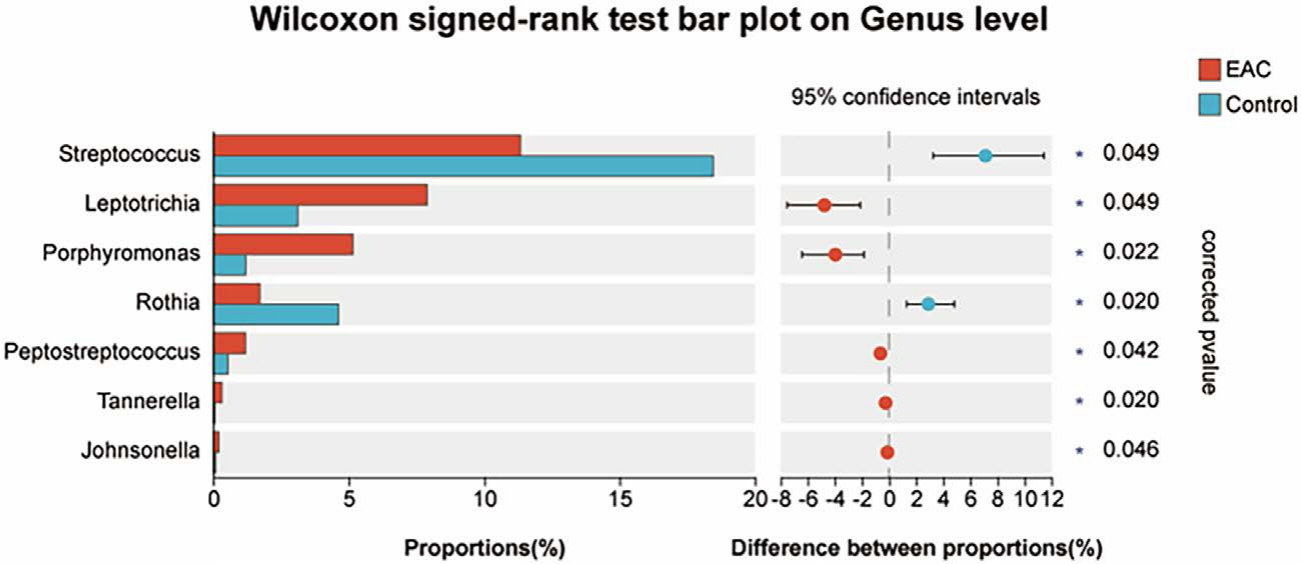
Significant differences in tongue coating microbiota between the EAC and control groups. Bar plot of Wilcox signed-rank test of the genera with significant differences between 2 groups (^*^P < .05). EAC = esophageal adenocarcinoma.

We used the Wilcoxon signed-rank test to explore the alterations in microbiota at the genus level. Herein, we identified 7 genera whose relative abundance differed significantly between the healthy and EAC groups (Fig. 4). Among them, *Leptotrichia, Porphyromonas, Peptostreptococcus, Tannerella*, and *Johnsonella* exhibited a significant increase in the EAC group, while *Streptococcus* and *Rothia* showed a significant decrease. These results identified significant alterations in genus-level abundance in EAC patients.

**Figure 5.**
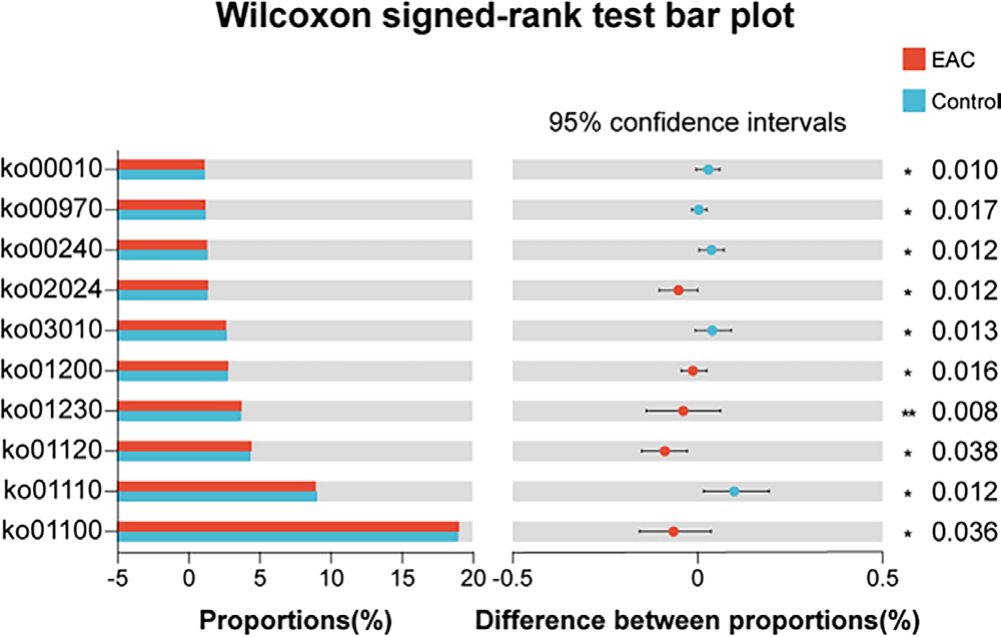
PICRUSt2 analysis between the EAC and control groups. There were significant differences in metabolic functions predicted. Bar plot of Wilcox signed-rank test of the top 10 metabolic functions with significant differences between the EAC and control groups (* *P* < .05, ** *P* < .01). EAC = esophageal adenocarcinoma, PICRUSt2 = Phylogenetic Investigation of Communities by Reconstruction of Unobserved States.

Next, we predicted metagenomics from 16S rRNA gene sequencing results using PICRUSt2 based on identifying KEGG pathways involved in samples from the EAC and controls. The Wilcoxon signed-rank test conducted on Pathway level3 abundance table (Table S1, Supplemental Digital Content, https://links.lww.com/MD/Q334) showed several metabolic pathways that differ significantly between the 2 groups (Fig. 5). Among the top 10 pathways, ko01100 (Metabolic pathways), ko01120 (Microbial metabolism in diverse environments), ko01230 (Biosynthesis of amino acids), ko01200 (Carbon metabolism) and ko02024 (Quorum sensing) were significantly increased in the EAC group, while ko01110 (Biosynthesis of secondary metabolites), ko03010 (Ribosome), ko00240 (Pyrimidine metabolism), ko00970 (Aminoacyl-tRNA biosynthesis) and ko00010 (Glycolysis/Gluconeogenesis) were significantly decreased in the EAC group. This analysis predicted functional differences in metabolic pathways between groups.

## 4. Discussion

Recently, the incidence of EAC has been steadily increasing in China. However, studies on EC-related microorganisms in China have predominantly focused on ESCC, with only a few assessing EAC. This study represents the first attempt to analyze the characteristics of EAC-related oral microbiota and predict associated metabolic pathways by tongue coating as an oral microbial sample in China. The Shannon index, which represents microbial community diversity, and PCoA analysis indicate significant differences in the composition and diversity of tongue coating microbiota between the 2 groups, suggesting significant changes in the tongue coating microbiota of EAC patients. This is inconsistent with previous studies that used saliva or mouthwash as oral microbiota samples for EAC,^[10,38,39]^ and may be due to the selection of different oral samples. Furthermore, our results, which demonstrate significant differences in the Shannon index between groups (*P* = .033), lend additional support to the usage of tongue coating as a sample for obtaining oral microbial characteristics among EAC patients. The observed increase in Shannon diversity and enrichment of genera like *Leptotrichia* align with prior reports linking oral dysbiosis to gastrointestinal inflammation.^[40]^ While these findings do not establish causality, they support further investigation into whether tongue coating microbiota could modulate EAC progression via inflammatory pathways.

In this study, oral *Leptotrichia, Porphyromonas, Peptostreptococcus, Tannerella*, and *Johnsonella* exhibited enrichment in the EAC group, whereas *Rothia* and *Streptococcus* showed decreased abundance. The relationship between tongue coating microbiota and EAC remains understudied. Snider et al found that increased abundance of *Streptococcus* and *Veillonella* in BE compared to controls in salivary microbiota.^[38]^ Furthermore, they also discovered that many genera were enriched in the control group, including *Neisseria, Lautropia*, and *Corynebacterium*. However, they did not identify risk-related genera for EAC. BE is a precancerous lesion for the development of EAC, and its microbiota changes might represent an early risk of EAC. Apart from the possible difference in results due to variations in oral samples, this may also explain the difference in the relative abundance of *Streptococcus* compared to our findings. Peters et al.^[10]^ examined the oral microbiota (prediagnostic mouthwash) and indicated that *Tannerella forsythia*, a periodontal pathogen, is positively correlated with EAC risk, which was consistent with our study. *T. forsythia* has been implicated in EC by facilitating nutrient supply and proliferation through GLUT-1 and GLUT-4 expression, as well as inducing pro-inflammatory cytokines.^[41]^ The detection of *T. forsythia* in precancerous lesions potentially becomes a major diagnostic and prognostic indicator of EAC. Moreover, *P. gingivalis*, producing gingipain K that affects the host immune system, has been associated with EAC.^[41]^

Our study revealed that oral *Leptotrichia, Peptostreptococcus, Rothia* and *Johnsonell* may be associated with EAC risk. Although these associations have not been previously reported, they have been found in studies on the EAC esophageal tissues or other tumors. Although *Leptotrichia* has extensive genetic diversity, with 6 species belonging to the genus identified to date,^[42]^ its exact relationship to human physiology remains undetermined. It is considered an opportunistic pathogen that is more likely to cause disease under conditions of local or systemic susceptibility. It has been described in several digestive cancers (e.g., esophagus,^[43]^ colon,^[44]^ gastric,^[45]^ and pancreatic^[46]^). In addition, *Leptotrichia* stimulates an immune response in humans.^[42]^ Thus, we can speculate that the immune activity induced by *Leptotrichia*, in addition to *Leptotrichia* itself, may also promote tumorigenesis.^[43]^ *Peptostreptoccus* was enriched in tongue coating from patients with precancerous esophageal lesions^[47]^ and was the most common oral microbiota in squamous oral cancer.^[48]^ A study on oral cancer-associated microbiota found that the relative abundance of *Rothia* was decreased in patients with oral cancer.^[9]^ A recent study showed that *Johnsonella ignava* is highly correlated with the tumor site of oral squamous carcinoma.^[49]^ Since the microbiota mentioned above have not been reported to be associated with EAC, we need large sample sizes to confirm the correlation and further study the potential mechanisms for EAC.

Microbial imbalance induces systemic metabolic changes^[50]^ and vice versa.^[51]^ Based on the results of the PICRUSt2 analyses, we can speculate that a variety of metabolic functions of the tongue coating microbiota may be altered in EAC. Among these functions, Ribosome, Pyrimidine metabolism, and Aminoacyl-tRNA biosynthesis are closely associated with cell proliferation, and unrestricted cell proliferation is a hallmark of cancer.^[52]^ Glycolysis and Carbon metabolism are intricately linked to oncogenes and tumor suppressors, providing selective advantages to rapidly proliferating cells.^[53]^ Recent studies have also highlighted the involvement of amino acid metabolism in cancer, particularly the role of glutamine.^[54]^ Therefore, changes in the tongue coating microbiota of EAC may be accompanied by alternations in metabolic function, and the interaction between these factors may contribute to the progression of EAC.

Our findings suggest that patients with EAC have a unique tongue coating microbiota. Many studies have developed diagnostic or predictive models for cancer based on unique oral microbiota characteristics. Zhou et al provided an oral microbiota-based diagnostic model that can be accurately and efficiently applied to a large-scale population screening program for oral squamous cell carcinoma.^[55]^ A recent study identified changes in the oral microbiota related to lung adenocarcinoma and constructed a predictive model for lung cancer based on microbiota changes.^[56]^ Based on the unique tongue coating microbiota features we identified in EAC patients, it may be possible to construct predictive or diagnostic models for EAC in the future, facilitating the development of noninvasive and effective methods for primary prevention and early detection of EAC.

Nevertheless, there were some limitations. First, the number of samples was limited, necessitating a large-scale study to substantiate these conclusions. Second, although this study attempted to control for possible factors that could affect the tongue coating microbiota, including dietary parameters and oral diseases, there are still many unknown or unmeasured potential confounding factors that cannot be completely excluded. Third, our study population was the Chinese Han population, which may limit the generalizability of the findings. Based on this study, in the future, we can explore the multi-omics integration analysis of EAC patients to increase the reliability of the results by mutual validation.

## 5. Conclusion

This exploratory case-control study presents novel findings on the differences in tongue coating microbiota and predicted metabolic functions between EAC patients and healthy individuals. Understanding these differences can pave the way for developing noninvasive methods for EAC based on the tongue coating microbiota composition in the future.

## Author contributions

**Conceptualization:** Huijie Wang, Xu Cao.

**Data curation:** Huijie Wang, Jinfeng Wang, Jinli Liu, Xiaoyang Shi, Zhichao Wang.

**Formal analysis:** Huijie Wang, Jinfeng Wang, Jinli Liu.

**Investigation:** Huijie Wang, Jinfeng Wang.

**Methodology:** Jinfeng Wang.

**Project administration:** Xu Cao.

**Software:** Jinli Liu.

**Supervision:** Xu Cao.

**Writing – original draft:** Huijie Wang, Xu Cao.

**Writing – review & editing:** Xu Cao.

## Supplementary Material




