# Supplementary figures and images for: Oral microbiota analysis of tongue coating in patients with esophageal adenocarcinoma
Source: Medicine (Baltimore). 2025 Oct 10;104(41):e45160. doi: 10.1097/MD.0000000000045160 (PMC12517899; doi:10.1097/MD.0000000000045160)

Supplementary Figure S1: A phylogenetic tree using the maximum likelihood method.


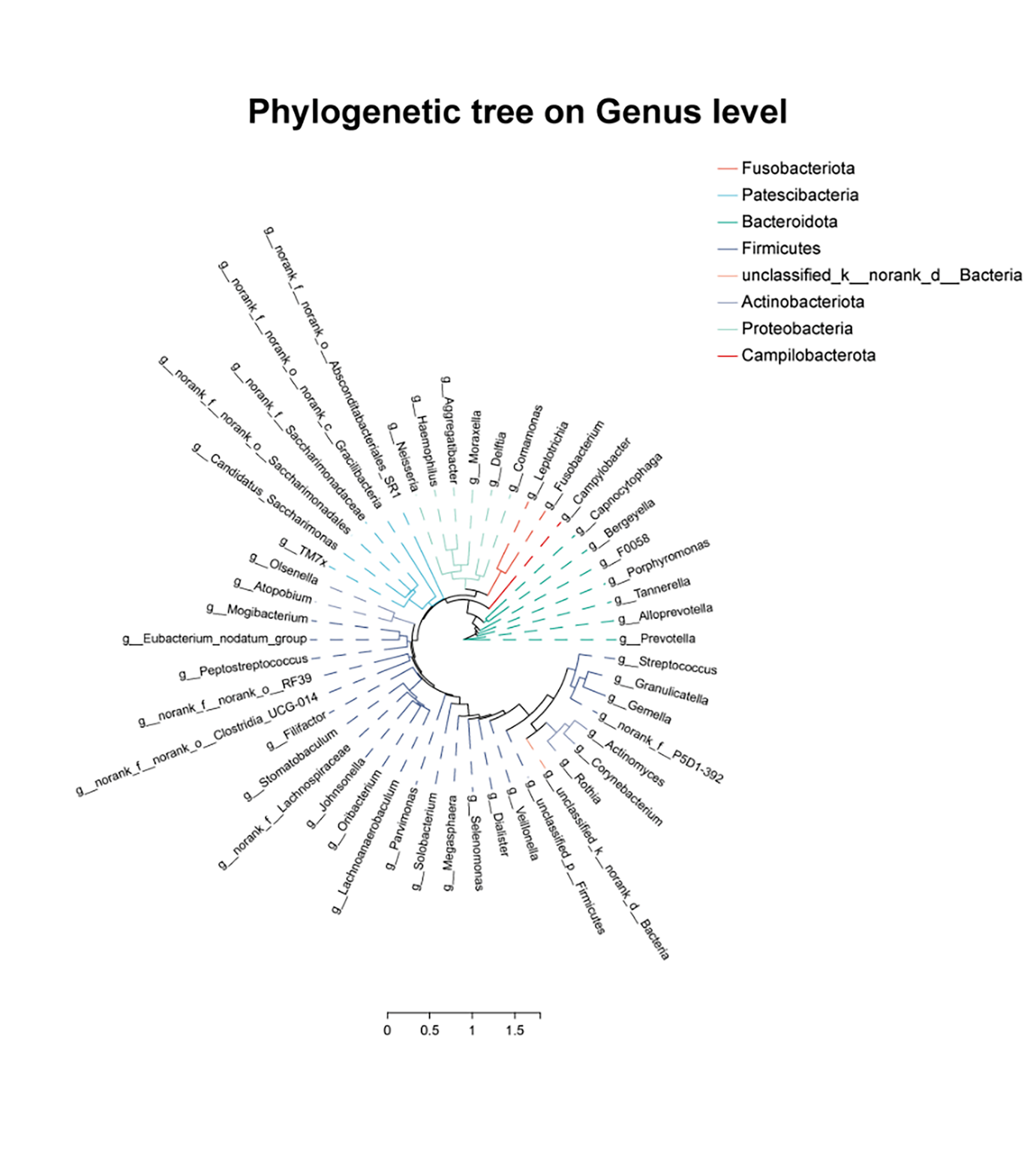

Supplement: Supplementary file 1 [file medi-104-e45160-s001.docx]
