# Supplementary material for: Oral microbiota analysis of tongue coating in patients with esophageal adenocarcinoma
Source: Medicine (Baltimore). 2025 Oct 10;104(41):e45160. doi: 10.1097/MD.0000000000045160 (PMC12517899; doi:10.1097/MD.0000000000045160)
Supplement: Supplementary file 2 [file medi-104-e45160-s002.doc]

| Pathway level1 | Pathway level2 | Pathway level3 |
| --- | --- | --- |
| Metabolism | Global and overview maps | ko01100 |
| Metabolism | Global and overview maps | ko01110 |
| Metabolism | Global and overview maps | ko01120 |
| Metabolism | Global and overview maps | ko01230 |
| Metabolism | Global and overview maps | ko01200 |
| Genetic Information Processing | Translation | ko03010 |
| Environmental Information Processing | Membrane transport | ko02010 |
| Metabolism | Nucleotide metabolism | ko00230 |
| Cellular Processes | Cellular community - prokaryotes | ko02024 |
| Metabolism | Nucleotide metabolism | ko00240 |
| Genetic Information Processing | Translation | ko00970 |
| Metabolism | Carbohydrate metabolism | ko00520 |
| Metabolism | Carbohydrate metabolism | ko00010 |
| Metabolism | Amino acid metabolism | ko00270 |
| Metabolism | Energy metabolism | ko00190 |
| Environmental Information Processing | Signal transduction | ko02020 |
| Metabolism | Carbohydrate metabolism | ko00620 |
| Genetic Information Processing | Replication and repair | ko03440 |
| Metabolism | Amino acid metabolism | ko00260 |
| Metabolism | Carbohydrate metabolism | ko00500 |
| Metabolism | Glycan biosynthesis and metabolism | ko00550 |
| Metabolism | Energy metabolism | ko00720 |
| Genetic Information Processing | Replication and repair | ko03430 |
| Metabolism | Metabolism of cofactors and vitamins | ko00860 |
| Metabolism | Amino acid metabolism | ko00250 |
| Metabolism | Amino acid metabolism | ko00400 |
| Metabolism | Carbohydrate metabolism | ko00030 |
| Genetic Information Processing | Replication and repair | ko03030 |
| Metabolism | Energy metabolism | ko00680 |
| Metabolism | Carbohydrate metabolism | ko00020 |
| Environmental Information Processing | Membrane transport | ko03070 |
| Genetic Information Processing | Folding, sorting and degradation | ko03060 |
| Metabolism | Carbohydrate metabolism | ko00630 |
| Metabolism | Carbohydrate metabolism | ko00051 |
| Metabolism | Global and overview maps | ko01210 |
| Metabolism | Energy metabolism | ko00710 |
| Metabolism | Metabolism of cofactors and vitamins | ko00790 |
| Metabolism | Glycan biosynthesis and metabolism | ko00540 |
| Genetic Information Processing | Folding, sorting and degradation | ko03018 |
| Metabolism | Amino acid metabolism | ko00300 |
| Metabolism | Metabolism of cofactors and vitamins | ko00670 |
| Metabolism | Global and overview maps | ko01212 |
| Metabolism | Metabolism of cofactors and vitamins | ko00770 |
| Metabolism | Carbohydrate metabolism | ko00052 |
| Metabolism | Carbohydrate metabolism | ko00650 |
| Metabolism | Carbohydrate metabolism | ko00640 |
| Metabolism | Lipid metabolism | ko00061 |
| Metabolism | Metabolism of terpenoids and polyketides | ko00900 |
| Cellular Processes | Cell growth and death | ko04112 |
| Metabolism | Lipid metabolism | ko00564 |
| Metabolism | Metabolism of cofactors and vitamins | ko00760 |
| Human Diseases | Drug resistance: antimicrobial | ko01501 |
| Metabolism | Metabolism of cofactors and vitamins | ko00730 |
| Genetic Information Processing | Replication and repair | ko03410 |
| Genetic Information Processing | Replication and repair | ko03420 |
| Environmental Information Processing | Membrane transport | ko02060 |
| Metabolism | Energy metabolism | ko00910 |

**Supplementary Table S1**: Pathway level3 abundance table in two groups.

| Metabolism | Amino acid metabolism | ko00290 |
| --- | --- | --- |
| Metabolism | Energy metabolism | ko00195 |
| Metabolism | Xenobiotics biodegradation and metabolism | ko00983 |
| Metabolism | Metabolism of cofactors and vitamins | ko00780 |
| Metabolism | Amino acid metabolism | ko00220 |
| Metabolism | Metabolism of other amino acids | ko00450 |
| Metabolism | Energy metabolism | ko00920 |
| Metabolism | Amino acid metabolism | ko00330 |
| Human Diseases | Drug resistance: antimicrobial | ko01502 |
| Metabolism | Biosynthesis of other secondary metabolites | ko00521 |
| Metabolism | Metabolism of other amino acids | ko00480 |
| Metabolism | Amino acid metabolism | ko00340 |
| Metabolism | Lipid metabolism | ko00561 |
| Environmental Information Processing | Signal transduction | ko04066 |
| Human Diseases | Drug resistance: antimicrobial | ko01503 |
| Metabolism | Metabolism of cofactors and vitamins | ko00740 |
| Cellular Processes | Cellular community - prokaryotes | ko02026 |
| Metabolism | Carbohydrate metabolism | ko00660 |
| Metabolism | Metabolism of cofactors and vitamins | ko00130 |
| Metabolism | Biosynthesis of other secondary metabolites | ko00261 |
| Human Diseases | Drug resistance: antineoplastic | ko01523 |
| Cellular Processes | Cellular community - prokaryotes | ko05111 |
| Organismal Systems | Endocrine system | ko04922 |
| Genetic Information Processing | Folding, sorting and degradation | ko04122 |
| Metabolism | Amino acid metabolism | ko00280 |
| Metabolism | Glycan biosynthesis and metabolism | ko00511 |
| Organismal Systems | Aging | ko04212 |
| Human Diseases | Cancer: overview | ko05230 |
| Metabolism | Metabolism of cofactors and vitamins | ko00750 |
| Genetic Information Processing | Transcription | ko03020 |
| Cellular Processes | Transport and catabolism | ko04146 |
| Cellular Processes | Cell growth and death | ko04217 |
| Metabolism | Metabolism of other amino acids | ko00471 |
| Metabolism | Amino acid metabolism | ko00310 |
| Metabolism | Metabolism of other amino acids | ko00460 |
| Metabolism | Lipid metabolism | ko00071 |
| Metabolism | Metabolism of terpenoids and polyketides | ko00523 |
| Organismal Systems | Endocrine system | ko03320 |
| Metabolism | Amino acid metabolism | ko00350 |
| Metabolism | Carbohydrate metabolism | ko00040 |
| Human Diseases | Infectious disease: bacterial | ko05132 |
| Metabolism | Amino acid metabolism | ko00360 |
| Human Diseases | Infectious disease: bacterial | ko05134 |
| Metabolism | Metabolism of other amino acids | ko00430 |
| Metabolism | Biosynthesis of other secondary metabolites | ko00333 |
| Cellular Processes | Transport and catabolism | ko04142 |
| Metabolism | Amino acid metabolism | ko00380 |
| Metabolism | Metabolism of other amino acids | ko00473 |
| Metabolism | Biosynthesis of other secondary metabolites | ko00401 |
| Human Diseases | Cardiovascular disease | ko05418 |
| Human Diseases | Infectious disease: bacterial | ko05152 |
| Organismal Systems | Environmental adaptation | ko04626 |
| Metabolism | Lipid metabolism | ko00600 |
| Cellular Processes | Cell growth and death | ko04216 |
| Human Diseases | Endocrine and metabolic disease | ko04931 |
| Organismal Systems | Environmental adaptation | ko04714 |
| Metabolism | Carbohydrate metabolism | ko00562 |
| Metabolism | Biosynthesis of other secondary metabolites | ko00960 |

| Human Diseases | Neurodegenerative disease | ko05016 |
| --- | --- | --- |
| Organismal Systems | Endocrine system | ko04920 |
| Cellular Processes | Cellular community - prokaryotes | ko02025 |
| Organismal Systems | Aging | ko04213 |
| Organismal Systems | Immune system | ko04621 |
| Metabolism | Carbohydrate metabolism | ko00053 |
| Metabolism | Metabolism of other amino acids | ko00410 |
| Cellular Processes | Cell motility | ko02030 |
| Environmental Information Processing | Signal transduction | ko04070 |
| Human Diseases | Infectious disease: bacterial | ko05120 |
| Metabolism | Biosynthesis of other secondary metabolites | ko00998 |
| Metabolism | Metabolism of terpenoids and polyketides | ko01051 |
| Metabolism | Biosynthesis of other secondary metabolites | ko00966 |
| Metabolism | Xenobiotics biodegradation and metabolism | ko00362 |
| Metabolism | Biosynthesis of other secondary metabolites | ko00525 |
| Environmental Information Processing | Signal transduction | ko04016 |
| Organismal Systems | Nervous system | ko04727 |
| Human Diseases | Neurodegenerative disease | ko05010 |
| Metabolism | Global and overview maps | ko01220 |
| Organismal Systems | Nervous system | ko04724 |
| Metabolism | Glycan biosynthesis and metabolism | ko00531 |
| Organismal Systems | Endocrine system | ko04910 |
| Human Diseases | Immune disease | ko05340 |
| Cellular Processes | Cell motility | ko02040 |
| Metabolism | Glycan biosynthesis and metabolism | ko00603 |
| Metabolism | Metabolism of terpenoids and polyketides | ko00908 |
| Environmental Information Processing | Signal transduction | ko04152 |
| Metabolism | Metabolism of cofactors and vitamins | ko00785 |
| Genetic Information Processing | Translation | ko03008 |
| Human Diseases | Cancer: overview | ko05206 |
| Metabolism | Biosynthesis of other secondary metabolites | ko00950 |
| Human Diseases | Infectious disease: bacterial | ko05133 |
| Metabolism | Biosynthesis of other secondary metabolites | ko00332 |
| Human Diseases | Infectious disease: bacterial | ko05130 |
| Metabolism | Biosynthesis of other secondary metabolites | ko00405 |
| Human Diseases | Neurodegenerative disease | ko05014 |
| Human Diseases | Cancer: overview | ko05200 |
| Human Diseases | Endocrine and metabolic disease | ko04940 |
| Organismal Systems | Endocrine system | ko04918 |
| Human Diseases | Drug resistance: antineoplastic | ko01524 |
| Environmental Information Processing | Signal transduction | ko04068 |
| Genetic Information Processing | Translation | ko03013 |
| Metabolism | Lipid metabolism | ko00590 |
| Human Diseases | Endocrine and metabolic disease | ko04930 |
| Human Diseases | Cancer: overview | ko05205 |
| Human Diseases | Infectious disease: viral | ko05165 |
| Human Diseases | Cancer: overview | ko05203 |
| Metabolism | Xenobiotics biodegradation and metabolism | ko00627 |
| Metabolism | Biosynthesis of other secondary metabolites | ko00524 |
| Organismal Systems | Aging | ko04211 |
| Metabolism | Glycan biosynthesis and metabolism | ko00513 |
| Metabolism | Glycan biosynthesis and metabolism | ko00604 |
| Metabolism | Metabolism of terpenoids and polyketides | ko01055 |
| Metabolism | Xenobiotics biodegradation and metabolism | ko00625 |
| Metabolism | Metabolism of other amino acids | ko00440 |
| Cellular Processes | Cell growth and death | ko04214 |
| Metabolism | Lipid metabolism | ko00121 |
| Metabolism | Biosynthesis of other secondary metabolites | ko00940 |

| Genetic Information Processing | Folding, sorting and degradation | ko04141 |
| --- | --- | --- |
| Environmental Information Processing | Signal transduction | ko04013 |
| Metabolism | Xenobiotics biodegradation and metabolism | ko00633 |
| Metabolism | Glycan biosynthesis and metabolism | ko00510 |
| Cellular Processes | Cell growth and death | ko04210 |
| Metabolism | Metabolism of terpenoids and polyketides | ko01053 |
| Metabolism | Xenobiotics biodegradation and metabolism | ko00626 |
| Human Diseases | Infectious disease: bacterial | ko05150 |
| Organismal Systems | Digestive system | ko04974 |
| Metabolism | Xenobiotics biodegradation and metabolism | ko00643 |
| Environmental Information Processing | Signal transduction | ko04151 |
| Metabolism | Lipid metabolism | ko00072 |
| Metabolism | Xenobiotics biodegradation and metabolism | ko00982 |
| Metabolism | Xenobiotics biodegradation and metabolism | ko00980 |
| Organismal Systems | Digestive system | ko04973 |
| Organismal Systems | Digestive system | ko04972 |
| Organismal Systems | Digestive system | ko04978 |
| Metabolism | Glycan biosynthesis and metabolism | ko00572 |
| Metabolism | Metabolism of cofactors and vitamins | ko00830 |
| Organismal Systems | Excretory system | ko04964 |
| Organismal Systems | Endocrine system | ko04915 |
| Human Diseases | Cancer: specific types | ko05215 |
| Organismal Systems | Immune system | ko04612 |
| Organismal Systems | Immune system | ko04657 |
| Organismal Systems | Endocrine system | ko04914 |
| Organismal Systems | Immune system | ko04659 |
| Human Diseases | Cancer: specific types | ko05211 |
| Human Diseases | Endocrine and metabolic disease | ko04934 |
| Human Diseases | Infectious disease: parasitic | ko05146 |
| Metabolism | Xenobiotics biodegradation and metabolism | ko00622 |
| Organismal Systems | Endocrine system | ko04614 |
| Human Diseases | Neurodegenerative disease | ko05012 |
| Human Diseases | Endocrine and metabolic disease | ko04932 |
| Organismal Systems | Endocrine system | ko04917 |
| Organismal Systems | Circulatory system | ko04260 |
| Metabolism | Xenobiotics biodegradation and metabolism | ko00621 |
| Human Diseases | Cancer: overview | ko05231 |
| Metabolism | Biosynthesis of other secondary metabolites | ko00311 |
| Human Diseases | Cancer: overview | ko05204 |
| Metabolism | Lipid metabolism | ko01040 |
| Human Diseases | Cancer: specific types | ko05225 |
| Metabolism | Glycan biosynthesis and metabolism | ko00571 |
| Metabolism | Biosynthesis of other secondary metabolites | ko00965 |
| Organismal Systems | Development and regeneration | ko04361 |
| Environmental Information Processing | Signal transduction | ko04072 |
| Metabolism | Xenobiotics biodegradation and metabolism | ko00361 |
| Environmental Information Processing | Signal transduction | ko04011 |
| Metabolism | Lipid metabolism | ko00140 |
| Cellular Processes | Cell growth and death | ko04113 |
| Metabolism | Lipid metabolism | ko00592 |
| Metabolism | Metabolism of terpenoids and polyketides | ko01054 |
| Cellular Processes | Transport and catabolism | ko04138 |
| Metabolism | Lipid metabolism | ko00591 |
| Metabolism | Metabolism of other amino acids | ko00472 |
| Human Diseases | Infectious disease: parasitic | ko05143 |
| Human Diseases | Infectious disease: parasitic | ko05142 |
| Metabolism | Lipid metabolism | ko00565 |
| Metabolism | Metabolism of terpenoids and polyketides | ko00903 |

| Metabolism | Metabolism of terpenoids and polyketides | ko00906 |
| --- | --- | --- |
| Genetic Information Processing | Folding, sorting and degradation | ko03050 |
| Human Diseases | Immune disease | ko05322 |
| Metabolism | Metabolism of terpenoids and polyketides | ko00281 |
| Human Diseases | Cancer: specific types | ko05219 |
| Human Diseases | Infectious disease: bacterial | ko05131 |
| Human Diseases | Infectious disease: bacterial | ko05100 |
| Metabolism | Metabolism of terpenoids and polyketides | ko00981 |
| Metabolism | Xenobiotics biodegradation and metabolism | ko00930 |
| Metabolism | Biosynthesis of other secondary metabolites | ko00941 |
| Metabolism | Biosynthesis of other secondary metabolites | ko00945 |
| Human Diseases | Neurodegenerative disease | ko05020 |
| Metabolism | Lipid metabolism | ko00120 |
| Metabolism | Xenobiotics biodegradation and metabolism | ko00623 |
| Cellular Processes | Transport and catabolism | ko04144 |
| Genetic Information Processing | Transcription | ko03040 |
| Organismal Systems | Immune system | ko04622 |
| Metabolism | Xenobiotics biodegradation and metabolism | ko00624 |
| Metabolism | Xenobiotics biodegradation and metabolism | ko00642 |
| Metabolism | Xenobiotics biodegradation and metabolism | ko00364 |
| Metabolism | Xenobiotics biodegradation and metabolism | ko00791 |
| Genetic Information Processing | Transcription | ko03022 |
| Genetic Information Processing | Replication and repair | ko03450 |
| Environmental Information Processing | Signal transduction | ko04071 |
| Human Diseases | Infectious disease: viral | ko05168 |
| Human Diseases | Infectious disease: viral | ko05160 |
| Human Diseases | Cancer: specific types | ko05210 |
| Human Diseases | Infectious disease: viral | ko05161 |
| Human Diseases | Infectious disease: viral | ko05163 |
| Human Diseases | Infectious disease: viral | ko05164 |
| Cellular Processes | Cell growth and death | ko04215 |
| Human Diseases | Infectious disease: viral | ko05169 |
| Human Diseases | Infectious disease: viral | ko05170 |
| Human Diseases | Infectious disease: viral | ko05167 |
| Human Diseases | Infectious disease: viral | ko05162 |
| Human Diseases | Cancer: specific types | ko05222 |
| Human Diseases | Infectious disease: parasitic | ko05145 |
| Human Diseases | Cardiovascular disease | ko05416 |
| Cellular Processes | Cell growth and death | ko04115 |
| Metabolism | Xenobiotics biodegradation and metabolism | ko00984 |
| Metabolism | Glycan biosynthesis and metabolism | ko00515 |
| Metabolism | Glycan biosynthesis and metabolism | ko00514 |
| Metabolism | Metabolism of terpenoids and polyketides | ko00909 |
| Human Diseases | Infectious disease: bacterial | ko05110 |
| Cellular Processes | Cell growth and death | ko04110 |
| Cellular Processes | Cell growth and death | ko04111 |
| Human Diseases | Cardiovascular disease | ko05410 |
| Cellular Processes | Cellular community - eukaryotes | ko04510 |
| Cellular Processes | Cell motility | ko04810 |
| Human Diseases | Cardiovascular disease | ko05414 |
| Human Diseases | Cardiovascular disease | ko05412 |
| Environmental Information Processing | Signaling molecules and interaction | ko04514 |
| Environmental Information Processing | Signaling molecules and interaction | ko04512 |
| Metabolism | Biosynthesis of other secondary metabolites | ko00944 |
| Organismal Systems | Nervous system | ko04728 |
| Human Diseases | Substance dependence | ko05034 |
| Human Diseases | Substance dependence | ko05031 |
| Human Diseases | Substance dependence | ko05030 |

| Organismal Systems | Nervous system | ko04726 |
| --- | --- | --- |
| Environmental Information Processing | Signal transduction | ko04024 |
| Metabolism | Xenobiotics biodegradation and metabolism | ko00365 |
| Organismal Systems | Endocrine system | ko04919 |
| Organismal Systems | Digestive system | ko04976 |
| Metabolism | Lipid metabolism | ko00100 |
| Metabolism | Biosynthesis of other secondary metabolites | ko00232 |
| Organismal Systems | Immune system | ko04640 |
| Genetic Information Processing | Translation | ko03015 |
| Metabolism | Glycan biosynthesis and metabolism | ko00563 |
| Human Diseases | Infectious disease: bacterial | ko05135 |
| Metabolism | Energy metabolism | ko00196 |
| Metabolism | Biosynthesis of other secondary metabolites | ko00404 |
| Organismal Systems | Endocrine system | ko04924 |
| Human Diseases | Neurodegenerative disease | ko05017 |
| Metabolism | Biosynthesis of other secondary metabolites | ko00901 |
| Metabolism | Metabolism of terpenoids and polyketides | ko00253 |
| Metabolism | Biosynthesis of other secondary metabolites | ko00943 |
| Environmental Information Processing | Signal transduction | ko04150 |
| Metabolism | Lipid metabolism | ko00062 |
| Organismal Systems | Nervous system | ko04721 |
| Organismal Systems | Nervous system | ko04723 |
| Cellular Processes | Transport and catabolism | ko04145 |
| Organismal Systems | Excretory system | ko04966 |
| Human Diseases | Immune disease | ko05323 |
| Metabolism | Metabolism of terpenoids and polyketides | ko01059 |
| Metabolism | Metabolism of terpenoids and polyketides | ko01052 |
| Metabolism | Xenobiotics biodegradation and metabolism | ko00363 |
| Metabolism | Metabolism of terpenoids and polyketides | ko01057 |
| Organismal Systems | Immune system | ko04624 |
| Organismal Systems | Endocrine system | ko04928 |
| Metabolism | Biosynthesis of other secondary metabolites | ko00997 |
| Organismal Systems | Endocrine system | ko04916 |
| Organismal Systems | Immune system | ko04062 |
| Human Diseases | Cancer: specific types | ko05220 |
| Cellular Processes | Cellular community - eukaryotes | ko04530 |
| Environmental Information Processing | Signal transduction | ko04020 |
| Organismal Systems | Development and regeneration | ko04320 |
| Cellular Processes | Cellular community - eukaryotes | ko04540 |
| Cellular Processes | Cellular community - eukaryotes | ko04520 |
| Organismal Systems | Immune system | ko04666 |
| Cellular Processes | Cellular community - eukaryotes | ko04550 |
| Human Diseases | Cancer: specific types | ko05221 |
| Organismal Systems | Immune system | ko04662 |
| Metabolism | Metabolism of terpenoids and polyketides | ko00905 |
| Human Diseases | Cancer: specific types | ko05224 |
| Human Diseases | Drug resistance: antineoplastic | ko01521 |
| Human Diseases | Drug resistance: antineoplastic | ko01522 |
| Human Diseases | Cancer: specific types | ko05213 |
| Environmental Information Processing | Signal transduction | ko04012 |
| Organismal Systems | Immune system | ko04664 |
| Human Diseases | Cancer: specific types | ko05226 |
| Human Diseases | Cancer: specific types | ko05214 |
| Organismal Systems | Endocrine system | ko04912 |
| Organismal Systems | Endocrine system | ko04935 |
| Environmental Information Processing | Signal transduction | ko04630 |
| Environmental Information Processing | Signal transduction | ko04010 |
| Human Diseases | Substance dependence | ko05032 |

| Organismal Systems | Immune system | ko04650 |
| --- | --- | --- |
| Environmental Information Processing | Signaling molecules and interaction | ko04080 |
| Organismal Systems | Nervous system | ko04722 |
| Human Diseases | Substance dependence | ko05033 |
| Human Diseases | Cancer: specific types | ko05223 |
| Environmental Information Processing | Signal transduction | ko04014 |
| Organismal Systems | Endocrine system | ko04926 |
| Organismal Systems | Immune system | ko04660 |
| Organismal Systems | Endocrine system | ko04923 |
| Environmental Information Processing | Signal transduction | ko04330 |
| Environmental Information Processing | Signal transduction | ko04310 |
| Organismal Systems | Excretory system | ko04962 |
| Cellular Processes | Cell growth and death | ko04218 |
| Human Diseases | Infectious disease: parasitic | ko05144 |
| Metabolism | Metabolism of terpenoids and polyketides | ko00902 |
| Organismal Systems | Nervous system | ko04725 |
| Metabolism | Glycan biosynthesis and metabolism | ko00601 |
| Environmental Information Processing | Signal transduction | ko04075 |

Description EAC_21 EAC_20 EAC_1

Metabolic pathways 8751612.75 8400827.32 8302223.85

Biosynthesis of secondary metabolites 4189718.89 4075736.71 3918145.47

Microbial metabolism in diverse environments 2079338.78 2029678.58 1938270.84

Biosynthesis of amino acids 1831791.11 1697720.95 1682442.92

Carbon metabolism 1314402.66 1266499.9 1204469.23

Ribosome 1192058.73 1168136.9 1161058.68

ABC transporters 1001500.98 1038756.06 1000114.8

Purine metabolism 788937.51 815433.52 769411.57

Quorum sensing 626775.46 640729.05 632761.76

Pyrimidine metabolism 563993.06 568504.18 571087.1

Aminoacyl-tRNA biosynthesis 547946.25 537295 537283.19

Amino sugar and nucleotide sugar metabolism 447277.2 514041.43 496033.24

Glycolysis / Gluconeogenesis 478088.08 513748.26 504081.75

Cysteine and methionine metabolism 527575.77 497067.94 505193.56

Oxidative phosphorylation 534073.54 535621.64 470473.87

Two-component system 527121.03 519084.32 456543.34

Pyruvate metabolism 511325.86 477990.85 471240.42

Homologous recombination 458095.28 456686.46 444494.52

Glycine, serine and threonine metabolism 409885.85 418503.63 399442.42

Starch and sucrose metabolism 327727.03 431042.64 414107.82

Peptidoglycan biosynthesis 406600.66 400218.59 398420.44

Carbon fixation pathways in prokaryotes 443804.36 373992.06 393502.5

Mismatch repair 394251.88 377111.38 384107.65

Porphyrin and chlorophyll metabolism 482146.45 348502.92 452805.16

Alanine, aspartate and glutamate metabolism 389467.79 367765.33 364598.01 Phenylalanine, tyrosine and tryptophan biosynthesis 387876.95 368626.25 346768.71 Pentose phosphate pathway 347408.54 342340.69 332111.07

DNA replication 326056.96 321607.06 316431.42

Methane metabolism 313886.6 323475.17 297842.81

Citrate cycle (TCA cycle) 341197.28 314066.56 283973.68

Bacterial secretion system 373665.04 295242.21 307701.31

Protein export 302033.11 287230.2 284408.63

Glyoxylate and dicarboxylate metabolism 334945.37 277008.35 279673.13

Fructose and mannose metabolism 215622.3 268555.33 274798.08

2-Oxocarboxylic acid metabolism 361900.27 293049.31 290274.47

Carbon fixation in photosynthetic organisms 275825.69 253695.06 260860.44

Folate biosynthesis 304978.4 263105.23 251830.66

Lipopolysaccharide biosynthesis 322067.85 218790.35 272522.65

RNA degradation 265714.93 260623.79 253249.11

Lysine biosynthesis 268337.01 256068.74 262722

One carbon pool by folate 250354.98 240401.05 251583.5

Fatty acid metabolism 269108.63 260617.24 245727.97

Pantothenate and CoA biosynthesis 281215.69 264268.31 253332.88

Galactose metabolism 182524.17 253220.23 238644.19

Butanoate metabolism 271690.15 220337.39 257041.14

Propanoate metabolism 299189.92 265118.21 269222.41

Fatty acid biosynthesis 266551.24 256426.73 241490.7

Terpenoid backbone biosynthesis 232251.71 234652.98 237513.21

Cell cycle - Caulobacter 236138.96 224855.65 228205.48

Glycerophospholipid metabolism 227106.72 273792.11 214270.62

Nicotinate and nicotinamide metabolism 217150.42 227091.73 210183.8

beta-Lactam resistance 257873.51 201774.02 223055.85

Thiamine metabolism 191033.59 189462.98 194857.79

Base excision repair 198946.18 195661.36 198696.14

Nucleotide excision repair 180099.58 192113.51 182404.25

Phosphotransferase system (PTS) 154249.33 170701.51 223319.2

Nitrogen metabolism 194328.72 179949.01 175334.96

| Valine, leucine and isoleucine biosynthesis | 220540.07 | 199710.62 | 181785.11 |
| --- | --- | --- | --- |
| Photosynthesis | 175395.04 | 169728.15 | 174161.74 |
| Drug metabolism - other enzymes | 150775.17 | 159823.08 | 156676.28 |
| Biotin metabolism | 203910.16 | 161856.88 | 156726.01 |
| Arginine biosynthesis | 200199.65 | 158412.14 | 164099.4 |
| Selenocompound metabolism | 151950.41 | 155961.12 | 156446.5 |
| Sulfur metabolism | 177622.17 | 170940.67 | 151328.82 |
| Arginine and proline metabolism | 175083.63 | 142473.62 | 148566.67 |
| Vancomycin resistance | 118715.05 | 129210.05 | 122980.89 |
| Streptomycin biosynthesis | 123730.94 | 135521.02 | 117095.31 |
| Glutathione metabolism | 140264.98 | 145628.88 | 115965.99 |
| Histidine metabolism | 172580.96 | 109654.05 | 138523.12 |
| Glycerolipid metabolism | 110621.29 | 138416.57 | 121538.86 |
| HIF-1 signaling pathway | 113828.33 | 114125.59 | 116016.08 |
| Cationic antimicrobial peptide (CAMP) resistance | 135914.33 | 113670.34 | 113112.63 |
| Riboflavin metabolism | 118034.79 | 90353.66 | 111115.95 |
| Biofilm formation - Escherichia coli | 92251.77 | 116409.12 | 93087.06 |
| C5-Branched dibasic acid metabolism | 134821.94 | 126298.9 | 113893.17 |
| Ubiquinone and other terpenoid-quinone biosynth | 121144.82 | 116219.03 | 108820.29 |
| Monobactam biosynthesis | 107923.35 | 107992.88 | 101378.31 |
| Antifolate resistance | 103982.16 | 103490.58 | 103533.47 |
| Biofilm formation - Vibrio cholerae | 107015.72 | 99871.41 | 103546.27 |
| Glucagon signaling pathway | 90701.47 | 109593.24 | 99632.25 |
| Sulfur relay system | 96155.37 | 111510.3 | 93139.34 |
| Valine, leucine and isoleucine degradation | 107394.68 | 97067.14 | 100682.9 |
| Other glycan degradation | 38623.5 | 97110.08 | 68994.51 |
| Longevity regulating pathway - worm | 101172.83 | 100517.52 | 90251.42 |
| Central carbon metabolism in cancer | 76982.54 | 92874.41 | 84667.85 |
| Vitamin B6 metabolism | 83673.12 | 89123.57 | 77698.49 |
| RNA polymerase | 87401.82 | 86597.07 | 87025.83 |
| Peroxisome | 74405.9 | 97450.21 | 71742.65 |
| Necroptosis | 71051.53 | 77893.12 | 72298.06 |
| D-Glutamine and D-glutamate metabolism | 76485.89 | 76253.56 | 74289.69 |
| Lysine degradation | 77849.43 | 83169.27 | 73671.27 |
| Cyanoamino acid metabolism | 68211.06 | 77972.02 | 68384.16 |
| Fatty acid degradation | 65994.43 | 78060.51 | 69233.68 |
| Polyketide sugar unit biosynthesis | 61336.71 | 71882.77 | 63438.06 |
| PPAR signaling pathway | 49210.64 | 81564.33 | 61971.62 |
| Tyrosine metabolism | 88732.9 | 63634.85 | 73482.5 |
| Pentose and glucuronate interconversions | 62488.09 | 68810.91 | 64995.53 |
| Salmonella infection | 66879.93 | 60884.35 | 68021.41 |
| Phenylalanine metabolism | 79986.21 | 56435.27 | 67791.38 |
| Legionellosis | 74246.93 | 64218.44 | 64129.66 |
| Taurine and hypotaurine metabolism | 70889.07 | 69820.44 | 63401.68 |
| Prodigiosin biosynthesis | 65699.26 | 54036.04 | 57299.98 |
| Lysosome | 25640 | 63809.16 | 44924.76 |
| Tryptophan metabolism | 78183.57 | 75472.66 | 58004.88 |
| D-Alanine metabolism | 54374.02 | 64364.44 | 58078.04 |
| Novobiocin biosynthesis | 69181.25 | 53882.52 | 59015.25 |
| Fluid shear stress and atherosclerosis | 60292.06 | 53344.07 | 57284.67 |
| Tuberculosis | 51812.19 | 51029.44 | 54799.86 |
| Plant-pathogen interaction | 45201.46 | 51932.63 | 52261.36 |
| Sphingolipid metabolism | 19610.34 | 52758.01 | 41281.85 |
| Ferroptosis | 35113.67 | 56907.28 | 43763.75 |
| Insulin resistance | 44162.9 | 59872.32 | 47441.52 |
| Thermogenesis | 50744.5 | 64532.45 | 44454 |
| Inositol phosphate metabolism | 47555.26 | 69857.65 | 44675.89 |
| Tropane, piperidine and pyridine alkaloid biosynthe | 52229.45 | 38591.55 | 47193.72 |

Huntington disease 47351.93 44985.69 40176.56

Adipocytokine signaling pathway 32438.67 57441.28 38728.17

Biofilm formation - Pseudomonas aeruginosa 45221.43 49973.28 33271.5

Longevity regulating pathway - multiple species 44268.47 50945.66 38042.41

NOD-like receptor signaling pathway 36702.13 33983.88 43670.28

Ascorbate and aldarate metabolism 43857.49 25285.45 52394.61

beta-Alanine metabolism 40708.49 43405.97 38954.59

Bacterial chemotaxis 26023.66 43379.98 30169.76

Phosphatidylinositol signaling system 50278.43 48678.18 38126.4

Epithelial cell signaling in Helicobacter pylori infecti 43775.62 31554.29 39184.53

Biosynthesis of various secondary metabolites - par 46773.93 39242.68 35602.73

Biosynthesis of ansamycins 37820.78 30768.22 39995.36

Glucosinolate biosynthesis 41562.1 39069.94 36366.74

Benzoate degradation 49274.24 25000.49 45106.07

Acarbose and validamycin biosynthesis 30871.44 36370.93 31384.24

MAPK signaling pathway - plant 49930.09 46536.94 36919.38

GABAergic synapse 27242.97 38632.23 32552.95

Alzheimer disease 50429.8 39765.72 33810.13

Degradation of aromatic compounds 57906.7 33963.25 40747.88

Glutamatergic synapse 26567.97 37085.06 31257.29

Glycosaminoglycan degradation 27498.67 19782.42 28737.83

Insulin signaling pathway 29028.94 40094.52 27787.49

Primary immunodeficiency 27161.94 38069.35 30363.84

Flagellar assembly 21758.5 27313.11 14482

Glycosphingolipid biosynthesis - globo and isoglob 10882.84 14139.17 20999.85

Zeatin biosynthesis 24975.63 24788.13 26475.7

AMPK signaling pathway 25369.3 31891.05 25926.12

Lipoic acid metabolism 27186.64 38172.98 23308.87

Ribosome biogenesis in eukaryotes 30507.8 32823.89 27100.37

MicroRNAs in cancer 24581.81 24081.98 27974.27

Isoquinoline alkaloid biosynthesis 32694.32 21526.58 28663.17

Pertussis 36750.5 17942.75 37993.5

Carbapenem biosynthesis 36092.92 32303.1 29514.22

Pathogenic Escherichia coli infection 30860.8 27017.72 26171.13

Phenazine biosynthesis 34696.76 27439.27 26308.08

Amyotrophic lateral sclerosis (ALS) 16421.13 19706.98 20637.84

Pathways in cancer 27314.8 27296.64 22168.45

Type I diabetes mellitus 23080.3 23509.97 24009.53

Thyroid hormone synthesis 11011.43 16254.76 20143.13

Platinum drug resistance 32307.8 29513.3 25038.04

FoxO signaling pathway 27293.97 30324.4 21203.89

RNA transport 17695.6 22174.18 18713.98

Arachidonic acid metabolism 15327.13 16750.63 19466.09

Type II diabetes mellitus 22722.3 21900.47 21900.79

Proteoglycans in cancer 22286.63 21875.13 21808.7

Human papillomavirus infection 22411.3 21845.22 21838.54

Viral carcinogenesis 22411.3 21843.22 21807.54

Aminobenzoate degradation 28275.9 25640.85 20238.92

Neomycin, kanamycin and gentamicin biosynthesis 17988.8 25068.3 19751.11

Longevity regulating pathway 25986.8 24614.23 18725.72

Various types of N-glycan biosynthesis 8297.67 9674.83 13408.17

Glycosphingolipid biosynthesis - ganglio series 8297.67 9674.83 13408.17

Biosynthesis of vancomycin group antibiotics 16505.47 20557.05 17369.87

Chloroalkane and chloroalkene degradation 25064.77 17067.8 20717.25

Phosphonate and phosphinate metabolism 19278.63 12546.31 17272.44

Apoptosis - fly 14211.63 11445.63 15969.53

Secondary bile acid biosynthesis 13638.79 12542.04 17996.7

Phenylpropanoid biosynthesis 13025.5 22551.19 13036.73

Protein processing in endoplasmic reticulum 8508.33 9495.93 12760.82

MAPK signaling pathway - fly 18674.3 18572.47 15313.22

Nitrotoluene degradation 28561.16 12647.33 16613.59

N-Glycan biosynthesis 12841.66 14133.67 13799

Apoptosis 4511 6834 10656.5

Biosynthesis of siderophore group nonribosomal pe 11262.35 11741.52 15018.1

Naphthalene degradation 22980.94 14166.94 17830.34

Staphylococcus aureus infection 11935.3 18776.7 16132.57

Protein digestion and absorption 5992 6887.17 9342.5

Styrene degradation 17879.43 15705.78 13159.28

PI3K-Akt signaling pathway 6103.67 12544.84 10332.67

Synthesis and degradation of ketone bodies 11108.63 7554.5 13271.2

Drug metabolism - cytochrome P450 21585.14 14525.47 12696.31

Metabolism of xenobiotics by cytochrome P450 21585.14 14525.47 12691.31

Carbohydrate digestion and absorption 6301.8 10005.14 10916.53

Pancreatic secretion 5990.8 9947.89 10823.28

Mineral absorption 15166.17 11079.84 10547.83

Arabinogalactan biosynthesis - Mycobacterium 10342.97 10154.39 13351.45

Retinol metabolism 16090.64 11577.47 11486.56

Proximal tubule bicarbonate reclamation 15579.67 13546.59 11187.26

Estrogen signaling pathway 4796.5 6832.67 7823.5

Prostate cancer 4796.5 6832.67 7823.5

Antigen processing and presentation 4796.5 6832.67 7823.5

IL-17 signaling pathway 4796.5 6832.67 7823.5

Progesterone-mediated oocyte maturation 4796.5 6832.67 7823.5

Th17 cell differentiation 4796.5 6832.67 7823.5

Renal cell carcinoma 14581.83 13602.58 10147.17

Cushing syndrome 14581.83 13602.58 10147.17

Amoebiasis 8171.8 4986.64 10834.53

Xylene degradation 15345.43 10087.71 11957.95

Renin-angiotensin system 8539.5 6938.75 6910.75

Parkinson disease 19626 12972 7656

Non-alcoholic fatty liver disease (NAFLD) 19626 12972 7656

Prolactin signaling pathway 4368.3 7686.14 6495.03

Cardiac muscle contraction 19569 12748 7638

Dioxin degradation 13557.3 7767.82 8928.03

Choline metabolism in cancer 5272.5 7732.5 5160.42

Penicillin and cephalosporin biosynthesis 8874.83 3198.25 7213.5

Chemical carcinogenesis 12905 8211.67 5098.6

Biosynthesis of unsaturated fatty acids 7665.67 7037.59 4069.67

Hepatocellular carcinoma 7879.47 6637.39 4179.78

Lipoarabinomannan (LAM) biosynthesis 1490.34 6082.84 7670.84

Betalain biosynthesis 12011 5859.42 5290

Axon regeneration 4055.5 7248.08 3903.92

Phospholipase D signaling pathway 4055.5 7248.08 3903.92

Chlorocyclohexane and chlorobenzene degradation 2546.13 3523.31 4207.58

MAPK signaling pathway - yeast 7312.5 6041.76 3412.5

Steroid hormone biosynthesis 312 1156 3152.5

Meiosis - yeast 1590.67 5747.92 2532.92

alpha-Linolenic acid metabolism 6847 4812.67 2569.25

Nonribosomal peptide structures 1887.5 1501.58 3887.18

Autophagy - yeast 1989.67 6229.09 2633.42

Linoleic acid metabolism 6816 4724.5 3061.75

D-Arginine and D-ornithine metabolism 5623.33 1672.17 4702.33

African trypanosomiasis 1535.67 5989.34 2436.02

Chagas disease (American trypanosomiasis) 1535.67 5989.34 2436.02

Ether lipid metabolism 6823 4724 2554.75

Limonene and pinene degradation 550 1270.52 2288.17

| Carotenoid biosynthesis 6571.5 | | 4481.27 | 2674 |
| --- | --- | --- | --- |
| Proteasome 1276.67 | | 5650.84 | 2493.67 |
| Systemic lupus erythematosus 6383 | | 3997 | 2528 |
| Geraniol degradation 269 | | 216.51 | 1145 |
| Bladder cancer 1161 | | 5563.75 | 1564.5 |
| Shigellosis 814 | | 1636.92 | 537.75 |
| Bacterial invasion of epithelial cells 2692.27 | | 1419.18 | 2039.87 |
| Insect hormone biosynthesis 538 | | 1237.02 | 1226.67 |
| Caprolactam degradation 14 | | 33.5 | 1079 |
| Flavonoid biosynthesis 925.5 | | 1927 | 1134.75 |
| Stilbenoid, diarylheptanoid and gingerol biosynthes 925.5 | | 1927 | 1134.75 |
| Prion diseases 540.5 | | 1414.59 | 294 |
| Primary bile acid biosynthesis 686 | | 957.67 | 1011.5 |
| Toluene degradation 22 | | 36.25 | 530 |
| Endocytosis 861.14 | | 1004.46 | 1309.94 |
| Spliceosome 2294 | | 280 | 781 |
| RIG-I-like receptor signaling pathway 418.33 | | 964.42 | 569 |
| Polycyclic aromatic hydrocarbon degradation 6 | | 0.012 | 23 |
| Ethylbenzene degradation 27 | | 88.67 | 20.5 |
| Fluorobenzoate degradation 19 | | 36.25 | 20 |
| Atrazine degradation 12 | | 28.35 | 46.5 |
| Basal transcription factors 42.5 | | 134.5 | 567.5 |
| Non-homologous end-joining 11 | | 7.17 | 13 |
| Sphingolipid signaling pathway 115.67 | | 84.67 | 351.67 |
| Herpes simplex virus 1 infection 122 | | 247 | 19 |
| Hepatitis C 57 | | 224 | 18 |
| Colorectal cancer 57 | | 224 | 18 |
| Hepatitis B 57 | | 224 | 18 |
| Human cytomegalovirus infection 57 | | 224 | 18 |
| Influenza A 57 | | 224 | 18 |
| Apoptosis - multiple species 57 | | 224 | 18 |
| Epstein-Barr virus infection 57 | | 224 | 18 |
| Human immunodeficiency virus 1 infection 57 | | 224 | 18 |
| Kaposi sarcoma-associated herpesvirus infection 57 | | 224 | 18 |
| Measles 57 | | 224 | 18 |
| Small cell lung cancer 57 | | 224 | 18 |
| Toxoplasmosis 57 | | 224 | 18 |
| Viral myocarditis 57 | | 224 | 18 |
| p53 signaling pathway 57 | | 224 | 18 |
| Steroid degradation 0.012 | | 0.012 | 20 |
| Mannose type O-glycan biosynthesis 50 | | 183 | 69 |
| Other types of O-glycan biosynthesis 50 | | 183 | 69 |
| Sesquiterpenoid and triterpenoid biosynthesis 27 | | 76.17 | 127 |
| Vibrio cholerae infection 196 | | 72 | 8 |
| Cell cycle 31 | | 39 | 40 |
| Cell cycle - yeast 31 | | 39 | 40 |
| Hypertrophic cardiomyopathy (HCM) 0.012 | | 2 | 31 |
| Focal adhesion 0.012 | | 2 | 31 |
| Regulation of actin cytoskeleton 0.012 | | 2 | 31 |
| Dilated cardiomyopathy (DCM) 0.012 | | 2 | 31 |
| Arrhythmogenic right ventricular cardiomyopathy (A0.012 | | 2 | 31 |
| Cell adhesion molecules (CAMs) | 0.012 | 2 | 31 |
| ECM-receptor interaction | 0.012 | 2 | 31 |
| Flavone and flavonol biosynthesis | 1 | 8.67 | 3 |
| Dopaminergic synapse | 0.012 | 0.012 | 6 |
| Alcoholism | 0.012 | 0.012 | 5 |
| Amphetamine addiction | 0.012 | 0.012 | 5 |
| Cocaine addiction | 0.012 | 0.012 | 5 |

| Serotonergic synapse | 0.012 | 0.012 | 5 |
| --- | --- | --- | --- |
| cAMP signaling pathway | 4 | 0.012 | 2 |
| Furfural degradation | 0.012 | 0.012 | 0.012 |
| Thyroid hormone signaling pathway | 0.012 | 0.012 | 0.012 |
| Bile secretion | 0.012 | 1 | 1 |
| Steroid biosynthesis | 1 | 0.17 | 1 |
| Caffeine metabolism | 0.012 | 0.012 | 1 |
| Hematopoietic cell lineage | 0.012 | 0.012 | 1 |
| mRNA surveillance pathway | 16 | 23 | 0.012 |
| Glycosylphosphatidylinositol (GPI)-anchor biosynth | 0.012 | 0.012 | 2 |
| Yersinia infection | 0.012 | 0.25 | 1 |
| Photosynthesis - antenna proteins | 0.012 | 0.012 | 0.012 |
| Staurosporine biosynthesis | 0.012 | 0.012 | 0.012 |
| Renin secretion | 0.012 | 0.012 | 0.012 |
| Spinocerebellar ataxia | 0.012 | 0.012 | 0.012 |
| Indole alkaloid biosynthesis | 0.012 | 0.012 | 0.012 |
| Tetracycline biosynthesis | 0.012 | 0.012 | 0.012 |
| Isoflavonoid biosynthesis | 0.012 | 0.012 | 0.012 |
| mTOR signaling pathway | 0.012 | 0.012 | 0.012 |
| Fatty acid elongation | 0.012 | 0.012 | 0.012 |
| Synaptic vesicle cycle | 0.012 | 0.012 | 0.012 |
| Retrograde endocannabinoid signaling | 0.012 | 0.012 | 0.012 |
| Phagosome | 0.012 | 0.012 | 0.012 |
| Collecting duct acid secretion | 0.012 | 0.012 | 0.012 |
| Rheumatoid arthritis | 0.012 | 0.012 | 0.012 |
| Biosynthesis of enediyne antibiotics | 0.012 | 0.012 | 1 |
| Type I polyketide structures | 0.012 | 0.012 | 1 |
| Bisphenol degradation | 0.012 | 0.012 | 1 |
| Biosynthesis of type II polyketide products | 0.012 | 0.012 | 0.012 |
| Toll and Imd signaling pathway | 0.012 | 0.012 | 0.012 |
| Parathyroid hormone synthesis, secretion and actio | 0.012 | 0.012 | 0.012 |
| Biosynthesis of various secondary metabolites - par | 1 | 0.012 | 0.012 |
| Melanogenesis | 0.012 | 0.17 | 0.012 |
| Chemokine signaling pathway | 0.012 | 0.012 | 1 |
| Chronic myeloid leukemia | 0.012 | 0.012 | 0.012 |
| Tight junction | 0.012 | 0.012 | 1 |
| Calcium signaling pathway | 0.012 | 0.012 | 0.012 |
| Dorso-ventral axis formation | 0.012 | 0.012 | 0.012 |
| Gap junction | 0.012 | 0.012 | 0.012 |
| Adherens junction | 0.012 | 0.012 | 1 |
| Fc gamma R-mediated phagocytosis | 0.012 | 0.012 | 1 |
| Signaling pathways regulating pluripotency of stem | 0.012 | 0.012 | 0.012 |
| Acute myeloid leukemia | 0.012 | 0.012 | 0.012 |
| B cell receptor signaling pathway | 0.012 | 0.012 | 0.012 |
| Brassinosteroid biosynthesis | 0.012 | 0.012 | 0.012 |
| Breast cancer | 0.012 | 0.012 | 0.012 |
| EGFR tyrosine kinase inhibitor resistance | 0.012 | 0.012 | 0.012 |
| Endocrine resistance | 0.012 | 0.012 | 0.012 |
| Endometrial cancer | 0.012 | 0.012 | 0.012 |
| ErbB signaling pathway | 0.012 | 0.012 | 0.012 |
| Fc epsilon RI signaling pathway | 0.012 | 0.012 | 0.012 |
| Gastric cancer | 0.012 | 0.012 | 0.012 |
| Glioma | 0.012 | 0.012 | 0.012 |
| GnRH signaling pathway | 0.012 | 0.012 | 0.012 |
| Growth hormone synthesis, secretion and action | 0.012 | 0.012 | 0.012 |
| Jak-STAT signaling pathway | 0.012 | 0.012 | 0.012 |
| MAPK signaling pathway | 0.012 | 0.012 | 0.012 |
| Morphine addiction | 0.012 | 0.012 | 0.012 |

Natural killer cell mediated cytotoxicity 0.012 0.012 0.012

Neuroactive ligand-receptor interaction 0.012 0.012 0.012

Neurotrophin signaling pathway 0.012 0.012 0.012

Nicotine addiction 0.012 0.012 0.012

Non-small cell lung cancer 0.012 0.012 0.012

Ras signaling pathway 0.012 0.012 0.012

Relaxin signaling pathway 0.012 0.012 0.012

T cell receptor signaling pathway 0.012 0.012 0.012

Regulation of lipolysis in adipocytes 0.012 0.012 0.012

Notch signaling pathway 0.012 0.012 0.012

Wnt signaling pathway 0.012 0.012 0.012

Vasopressin-regulated water reabsorption 0.012 0.012 0.012

Cellular senescence 0.012 0.012 0.012

Malaria 0.012 0.012 0.012

Monoterpenoid biosynthesis 0.012 0.012 0.012

Cholinergic synapse 0.012 0.17 0.012

Glycosphingolipid biosynthesis - lacto and neolacto 0.012 0.012 0.012

Plant hormone signal transduction 0.012 0.012 0.012

| EAC_8 | EAC_9 | EAC_10 | EAC_11 | EAC_12 | EAC_2 |
| --- | --- | --- | --- | --- | --- |
| 8214984.87 | 9177905.56 | 8652149.13 | 7800679.54 | 8085039.73 | 9352061.18 |
| 3965984.33 | 4302080.34 | 4152619.18 | 3647493.84 | 3826711.31 | 4277516.83 |
| 1846744.44 | 2184537.42 | 2031975.81 | 1866908.46 | 1879240.22 | 2176427.18 |
| 1620815.95 | 1762734.14 | 1810985.36 | 1451147.5 | 1578592.46 | 1668558.89 |
| 1226527.11 | 1428291.32 | 1272511.15 | 1195273.73 | 1191918.86 | 1422731.67 |
| 1207277.67 | 1276576.42 | 1221961.77 | 1131549.07 | 1149627.46 | 1307601.81 |
| 738838.83 | 1082998.85 | 1061399.55 | 1012879.77 | 911105.33 | 887151.56 |
| 786044.76 | 847831.48 | 803564.54 | 758667.09 | 770153.83 | 846320.87 |
| 531697.84 | 639716.53 | 654153.32 | 591259.72 | 590570.81 | 602352.18 |
| 614623.3 | 605075.58 | 605863.29 | 558188.81 | 584350.22 | 641183.59 |
| 536863.08 | 576708.1 | 565876.67 | 510087.34 | 521970.74 | 599358.63 |
| 530683.73 | 504308.12 | 544002.09 | 496793.13 | 499953.18 | 530156.95 |
| 546539.26 | 515654.13 | 553458.42 | 473007.89 | 492161.99 | 540808.55 |
| 477065.05 | 524462.07 | 503251.32 | 426062.18 | 479173.84 | 508878.78 |
| 511072.74 | 579709.75 | 486384.79 | 436540.6 | 466375.99 | 545329.62 |
| 389126.62 | 561832.26 | 446794.45 | 499099.13 | 486135.83 | 516293.22 |
| 438755.96 | 542112.24 | 489834.57 | 448371.56 | 447223.02 | 528222.48 |
| 464194.79 | 494786.05 | 479857.52 | 438757.79 | 442586.37 | 492786.7 |
| 427700.28 | 446112.32 | 425914.36 | 389328.16 | 399018.43 | 423568.79 |
| 437428.78 | 371858.31 | 466753.62 | 355926.32 | 387449.2 | 307493.69 |
| 422644.75 | 428460.15 | 413973.94 | 385900.08 | 405086.79 | 436270.88 |
| 421964.8 | 459080.43 | 402394.81 | 365637.58 | 403104.17 | 486826.55 |
| 394870.38 | 422829.57 | 408623.96 | 369137.15 | 374431.64 | 416057.31 |
| 315529.27 | 411559.54 | 456872.94 | 316604.95 | 341692.67 | 541909.78 |
| 391894.43 | 418918.89 | 382157.55 | 330489.58 | 365336.41 | 401857.45 |
| 348903.83 | 367552.27 | 372778.56 | 307310.41 | 338205.22 | 359786.11 |
| 318760.3 | 360004.88 | 350675.75 | 315532.22 | 318367.09 | 363828.49 |
| 325560.68 | 346334.92 | 336721.76 | 310083.51 | 310472.42 | 350122.13 |
| 331277.79 | 347787.62 | 335074.95 | 314024.68 | 311586.65 | 334337.55 |
| 323155.17 | 359352.48 | 295615.99 | 280144.89 | 299802.34 | 381447.78 |
| 266217.65 | 401987.39 | 314138.63 | 310246.36 | 283463.86 | 324970.05 |
| 276497.98 | 310565.81 | 304324.59 | 278556.67 | 276703.76 | 315780.16 |
| 284095.94 | 327128.15 | 275013.04 | 266339.75 | 295481.83 | 347720.41 |
| 315218.89 | 245728.51 | 292653.17 | 250388.84 | 279049.13 | 259978.76 |
| 250295.33 | 334926.11 | 319094.11 | 250463.45 | 263085.61 | 291271.96 |
| 276159.8 | 293215.3 | 281421 | 248894.61 | 258936.99 | 285999.8 |
| 260524.41 | 297303.79 | 260288.48 | 255555.99 | 262083.2 | 340584.33 |
| 260189.9 | 302777.6 | 277753.71 | 238050.83 | 254715.45 | 349026.15 |
| 254629.12 | 280108.59 | 266308.68 | 253424.08 | 249922.8 | 304243.95 |
| 260481.44 | 272092.79 | 270804.45 | 228771.13 | 248695.96 | 274651.26 |
| 275197.99 | 270919.58 | 265269.92 | 228651.33 | 251409.29 | 267790.4 |
| 236448.03 | 310325.88 | 268772.7 | 274513.9 | 237347.64 | 291846.42 |
| 248875.7 | 277820.41 | 264619.74 | 231718.84 | 248289.18 | 278829.48 |
| 276227.58 | 213064.39 | 269261.06 | 221433.45 | 243116.45 | 222148.49 |
| 239037.96 | 284290.69 | 252978.4 | 231537.52 | 247713.75 | 320148.83 |
| 203032.18 | 299262.87 | 258365.17 | 234796.91 | 236927.96 | 293345.93 |
| 233686.8 | 298187.29 | 260603.65 | 258039.11 | 229899.04 | 278076.61 |
| 240391.37 | 248771.32 | 242811.28 | 221126.09 | 231731.62 | 268616.9 |
| 251028.41 | 252725.11 | 250862.84 | 222637.74 | 228348.46 | 257637.35 |
| 210210.44 | 283084.43 | 239622.1 | 246859.82 | 219873.54 | 262987.64 |
| 244508.23 | 268132.36 | 227963.19 | 216764.57 | 221299.39 | 274886.76 |
| 215239.77 | 253223.66 | 216284.13 | 189392.84 | 217616.71 | 213807.58 |
| 202394.77 | 241896.2 | 198624.52 | 199846.08 | 196118.89 | 227738.19 |
| 204149.84 | 218939.23 | 207028.72 | 186245.53 | 198727.15 | 217493.82 |
| 198888.14 | 189004.68 | 197735.88 | 174364.15 | 182676.41 | 207716.76 |
| 134075.01 | 161528.55 | 221580.05 | 173123.81 | 179030.46 | 128159.09 |
| 170199.61 | 175427.04 | 172727.75 | 146293.13 | 179134.03 | 191341.76 |

| 137421.56 | 199408.72 | 196301.58 | 162199.64 | 158430.44 | 175657.43 |
| --- | --- | --- | --- | --- | --- |
| 182609.98 | 169531.49 | 176312.31 | 141595.91 | 170491.86 | 149287.89 |
| 177438.42 | 166622.96 | 164109.2 | 160480.55 | 166071.14 | 173031.64 |
| 132924.6 | 234341.69 | 167033.38 | 194337.2 | 150276.74 | 238497.55 |
| 150327.04 | 188260.93 | 170911.73 | 132542.6 | 157473.04 | 166771.13 |
| 143526.9 | 155846.03 | 170797.81 | 148810.74 | 146050.65 | 141812.83 |
| 134823.86 | 205800.77 | 182928.85 | 165988.59 | 140778.27 | 163465.45 |
| 137844.71 | 176838.88 | 149877.11 | 135519.87 | 148228.34 | 169536.71 |
| 132807.11 | 132909.56 | 137037.36 | 128911.68 | 126382.69 | 151403.11 |
| 142762.72 | 149947.87 | 131412.8 | 113939.72 | 117862.77 | 144956.66 |
| 104329.38 | 166668.98 | 118499.05 | 144989.38 | 125168.39 | 152002.15 |
| 130452.92 | 149897.7 | 143869.22 | 92760.77 | 123269.2 | 150036.05 |
| 102854.53 | 125176.13 | 121851.55 | 110596.17 | 117491.58 | 102385.97 |
| 113522.71 | 114182.22 | 127853.26 | 112965.62 | 107675.87 | 126481.41 |
| 107742.86 | 149042.15 | 116123.61 | 122382.03 | 117491.18 | 122878.28 |
| 127915.23 | 134511.49 | 121386.85 | 101378.85 | 111841.05 | 132640.33 |
| 109803.7 | 126022.55 | 117059.81 | 130434.98 | 113250.81 | 97945.6 |
| 99068.3 | 121437.18 | 120259.76 | 99488.44 | 107853.59 | 99029.33 |
| 104546.28 | 108296.83 | 91845.38 | 90459.47 | 108972.94 | 126382.15 |
| 113157.24 | 117612.26 | 114908.87 | 104672.67 | 105400.53 | 112710.22 |
| 115549.28 | 110909.21 | 111479.43 | 97450.11 | 104275.71 | 112281.4 |
| 102902.61 | 114703.34 | 111997.94 | 107315.99 | 107550.4 | 102520.29 |
| 95247.59 | 96853.15 | 115469.58 | 107668.13 | 93517.28 | 108212.36 |
| 72557.37 | 116074.62 | 107177.65 | 120515.42 | 92544.75 | 104336.62 |
| 70963.9 | 99050.91 | 96766.45 | 105768.58 | 92960.1 | 135239.87 |
| 116873.7 | 65275.78 | 86687.5 | 80479.85 | 84804.65 | 120244.6 |
| 82790.77 | 112534.06 | 94841.61 | 100170.76 | 87235.09 | 123094.42 |
| 74001.99 | 80145.88 | 95273.37 | 95009.68 | 76357.12 | 100991.01 |
| 92942.65 | 102134.8 | 83051.27 | 85212.62 | 83484.05 | 102909.49 |
| 81341.7 | 91921.63 | 91027.08 | 81262.45 | 80619.04 | 90997.85 |
| 83756.58 | 87049.24 | 83427.73 | 85659.12 | 77362.12 | 96669.51 |
| 81406.8 | 71868.54 | 79343.02 | 63634.82 | 74702.38 | 74401.24 |
| 73498.67 | 77774.27 | 79234.54 | 71729.15 | 71878.88 | 84727.24 |
| 54943.52 | 104281.43 | 74585.59 | 80920.12 | 64152.58 | 95625.16 |
| 80546 | 79560.34 | 71752.79 | 71186.24 | 72464.46 | 71072.34 |
| 66907.62 | 82207.9 | 71763.66 | 75295.87 | 69575.54 | 76671.63 |
| 81155.3 | 77914.26 | 77341.1 | 64067.07 | 63574.79 | 81881.75 |
| 74816.72 | 66349.3 | 68529.5 | 62431.67 | 67257.78 | 64173.51 |
| 58663.67 | 97668.82 | 66646.96 | 67136.89 | 69059.8 | 75226.16 |
| 80903.46 | 65169.86 | 71878.92 | 60023.96 | 66992.88 | 64922.94 |
| 72149.2 | 72740 | 73906.17 | 62648.71 | 64270.04 | 74645.81 |
| 53995.68 | 74693.98 | 61612.79 | 59537.06 | 70187.36 | 68694.7 |
| 67063.36 | 77996.03 | 63020.39 | 59049.62 | 62263.61 | 77101.31 |
| 56652.74 | 75524.4 | 67775.28 | 63279.56 | 59099.78 | 69357.66 |
| 66412.29 | 75479.2 | 63875.72 | 65670.64 | 61987.04 | 71062.54 |
| 78684.02 | 42799.1 | 56131.92 | 52524.76 | 55833.24 | 86644.69 |
| 39531.73 | 91561.58 | 54444.96 | 75547.38 | 56835.15 | 89977.78 |
| 53701.65 | 56258.12 | 62407.1 | 55121.43 | 56864.64 | 59827.27 |
| 56341.79 | 62179.61 | 60210.14 | 45549.87 | 56512.72 | 59072.98 |
| 54919.25 | 62236.89 | 62561.47 | 54488.89 | 55406.2 | 61643.75 |
| 61042.22 | 56547.96 | 56403.52 | 52182.34 | 56147.78 | 56166.84 |
| 53941.73 | 53626.22 | 57656.33 | 52908.36 | 53027.3 | 61891.36 |
| 67541.93 | 37327.24 | 49148.17 | 38922.34 | 51750.24 | 51364.06 |
| 61851.67 | 44109.35 | 49545.44 | 44160.57 | 49051.06 | 54921.83 |
| 55173.24 | 48656.1 | 56385.12 | 44281.72 | 48412.48 | 42277.26 |
| 52105.5 | 62170.44 | 47347.85 | 44908.65 | 46834.47 | 61420.6 |
| 49038.2 | 52268.48 | 47492.72 | 41869.3 | 45660.72 | 48466.91 |
| 48980.65 | 49529.24 | 48845.58 | 34056.77 | 46492.81 | 46447.63 |

| 43380.2 | 61641.57 | 38222.23 | 46567.46 | 44047.79 | 60630.56 |
| --- | --- | --- | --- | --- | --- |
| 51585.33 | 38842.77 | 45199.02 | 36769.31 | 42170.65 | 46757.6 |
| 34788.11 | 50489.41 | 45920.17 | 59038.86 | 49961.78 | 36895.41 |
| 34786.55 | 55221.18 | 45275.58 | 55199.59 | 37788.81 | 63599.23 |
| 48498.98 | 39330.82 | 50210.49 | 36403.19 | 39067.73 | 42994.73 |
| 35375.34 | 33884.58 | 43622.87 | 31640.01 | 45687.11 | 43107.71 |
| 40019.59 | 47584.03 | 38984.28 | 40683.58 | 40197.61 | 57427.17 |
| 45788.68 | 33303.37 | 45123.37 | 41367.34 | 44726.63 | 26832.48 |
| 31969.11 | 60264.47 | 41949.72 | 47598.8 | 38899.31 | 48937.76 |
| 39939.73 | 48776.98 | 40362.32 | 33148.87 | 36567.23 | 46684.48 |
| 33102.03 | 55751.82 | 34753.88 | 43756.8 | 35621.06 | 42765.31 |
| 38809.65 | 33903.42 | 46334.42 | 32400.28 | 36386.41 | 33636.99 |
| 34526.78 | 38943.55 | 41441.97 | 34725.7 | 34569.81 | 38633.66 |
| 19027.55 | 30241.77 | 29428.8 | 35319.55 | 43478.64 | 39687.7 |
| 40784.94 | 38895.22 | 38496.97 | 33174.7 | 32701.73 | 39580.5 |
| 21755.03 | 43832.57 | 34693.22 | 36494.79 | 31633.39 | 44810.36 |
| 34152.8 | 32449.41 | 35107.17 | 29984.18 | 31758.74 | 33369.98 |
| 28594.22 | 58664.18 | 32215.99 | 35161.52 | 31620.8 | 48417.08 |
| 20781.56 | 56836.94 | 27758.18 | 35413.84 | 36163.38 | 37936.72 |
| 33767.8 | 30270.08 | 33715.83 | 28356.18 | 31173.74 | 31703.48 |
| 35314.17 | 28827.5 | 27652.17 | 29538 | 31679.16 | 57517.7 |
| 32743.52 | 35250.73 | 38855.72 | 33076.12 | 28827.15 | 26577.58 |
| 33694.52 | 36386.83 | 32893.55 | 26774.12 | 29492.15 | 28084.18 |
| 48969.75 | 18920.4 | 37522 | 30284.25 | 35616.28 | 18346.15 |
| 37561.84 | 20018.67 | 23978.33 | 23115.67 | 27267.16 | 41185.3 |
| 33998.89 | 28980.02 | 28209.15 | 25669.19 | 28468.99 | 34216.98 |
| 27099.72 | 28488.1 | 30486.16 | 28834.85 | 27411.9 | 27861.33 |
| 25064.96 | 36463.29 | 29217.91 | 35911.1 | 23751.4 | 39813.73 |
| 25895.72 | 34620.35 | 28253.49 | 28616.35 | 26801.32 | 32634.58 |
| 34955.96 | 26907 | 26554 | 24015.18 | 27066.99 | 33889.43 |
| 27809.18 | 32543.95 | 28036.75 | 20965.42 | 27926.07 | 29654.05 |
| 10768 | 29233.9 | 26718.7 | 23013 | 20279.99 | 50606.3 |
| 21487.96 | 30211.58 | 30791.14 | 22946.88 | 25510.32 | 24952.66 |
| 26046.22 | 33582.68 | 27396.33 | 26270.52 | 25658.31 | 31656.08 |
| 25577.28 | 31144.93 | 27018.47 | 23067.2 | 27108.15 | 29835.66 |
| 31073.06 | 28069.36 | 19537.49 | 25004.86 | 29442.31 | 27762.98 |
| 19399.88 | 33419.58 | 21562.17 | 29424.51 | 25887.53 | 32202.25 |
| 23165.72 | 26280.68 | 24806.66 | 23099.02 | 22311.15 | 27166.58 |
| 30796.7 | 20004.49 | 17560.21 | 18594.3 | 27172.82 | 17154.41 |
| 14334.72 | 28351.78 | 23638.83 | 24498.02 | 21885.02 | 27927.43 |
| 13616.97 | 32137.04 | 23045.91 | 30069.76 | 18050.32 | 41359.58 |
| 20464.86 | 30054.25 | 22034.55 | 30243.8 | 21317.07 | 29380.76 |
| 30919.56 | 24409.11 | 17643.24 | 16969.11 | 25578.91 | 20953.98 |
| 23323.72 | 22053.86 | 23563.65 | 21079.02 | 21511.73 | 24641.08 |
| 22751.39 | 23777.02 | 23180.65 | 21177.69 | 21565.49 | 24887.08 |
| 22997.72 | 22047.36 | 23342.65 | 21058.52 | 21434.98 | 24578.08 |
| 22996.72 | 22033.36 | 23307.65 | 21034.52 | 21406.98 | 24576.08 |
| 7451.99 | 40087.51 | 16097.71 | 29669.14 | 22995.54 | 26708.71 |
| 25562.22 | 21433.28 | 23117.48 | 19479.52 | 19725.99 | 23005.23 |
| 11558.14 | 30274.71 | 20261.74 | 28234.6 | 16159.99 | 38999.58 |
| 23999.17 | 14053.67 | 16629.33 | 19839.5 | 16695.83 | 36466.8 |
| 23999.17 | 14053.67 | 16629.33 | 19839.5 | 16695.83 | 36466.8 |
| 20587.47 | 22302.51 | 21144.4 | 20044.6 | 17728.49 | 21844.25 |
| 13152.53 | 29972.25 | 17939.71 | 20127.97 | 16654.21 | 14541.43 |
| 21539.31 | 15696.45 | 16625.76 | 15703.94 | 20766.15 | 15177.33 |
| 18542.55 | 14446.35 | 19217.07 | 18885.77 | 18830.06 | 17645.5 |
| 18976.81 | 14168.54 | 16885.57 | 15439.62 | 19108.4 | 10631.41 |
| 22230.25 | 17472.31 | 15652.59 | 14351.25 | 14462.16 | 14037.1 |

| 26789.42 | 13132.89 | 14914.42 | 12656.25 | 18992.99 | 14446.55 |
| --- | --- | --- | --- | --- | --- |
| 10658.64 | 19382.71 | 17062.74 | 20453.6 | 12401.49 | 27553.08 |
| 10000.34 | 15484.91 | 17325.93 | 17351.75 | 18138.74 | 24188 |
| 17781.59 | 11045.72 | 15093.59 | 12538.75 | 14179.25 | 17762.48 |
| 22675 | 11647.5 | 11142 | 10138 | 16450.32 | 18992.8 |
| 17134.65 | 8189.9 | 14251.12 | 9686.23 | 14002.75 | 12768.4 |
| 10761.28 | 27270.77 | 14820.13 | 14508.38 | 12914.23 | 12733.86 |
| 5336 | 16002.96 | 9938.05 | 15234.3 | 14682.23 | 6941.75 |
| 22860 | 10765.5 | 11071.5 | 12102 | 13907.5 | 19013.8 |
| 3858.77 | 24823.56 | 9693.88 | 16998.12 | 11185.39 | 17020.78 |
| 15338.58 | 12090.73 | 11724.59 | 11041.24 | 11706.91 | 14414.15 |
| 7854.06 | 9053.28 | 12916.81 | 13061.69 | 11503.78 | 9351.43 |
| 6155.14 | 26349.97 | 10488.08 | 13937.94 | 10476.87 | 15833.93 |
| 6153.14 | 26346.97 | 10472.08 | 13925.94 | 10463.04 | 15832.93 |
| 19159.64 | 5400.55 | 12123.49 | 5708.52 | 11591.33 | 5439.83 |
| 18832.64 | 5380.05 | 11867.49 | 5664.02 | 11486.58 | 5374.83 |
| 12021.33 | 20124.16 | 7401.67 | 8962.83 | 11722.83 | 13071.4 |
| 6471.47 | 5419.28 | 8799.74 | 6984.26 | 9732.58 | 8185.6 |
| 10770.14 | 18072.72 | 9936.65 | 9548.19 | 7934.15 | 10280.93 |
| 6085.58 | 13004.89 | 10246.51 | 8990.49 | 8828.24 | 11882.5 |
| 13278.75 | 10214.4 | 8905.42 | 9199.08 | 9788.58 | 12052.15 |
| 13278.75 | 10214.4 | 8905.42 | 9199.08 | 9788.58 | 12052.15 |
| 13278.75 | 10214.4 | 8905.42 | 9199.08 | 9788.58 | 12052.15 |
| 13278.75 | 10214.4 | 8905.42 | 9199.08 | 9788.58 | 12052.15 |
| 13278.75 | 10214.4 | 8905.42 | 9199.08 | 9788.58 | 12052.15 |
| 13278.75 | 10214.4 | 8905.42 | 9199.08 | 9788.58 | 12052.15 |
| 4885 | 12622.07 | 8869.75 | 9622.25 | 8844.41 | 12266.5 |
| 4885 | 12622.07 | 8869.75 | 9622.25 | 8844.41 | 12266.5 |
| 11014.64 | 11550.8 | 9490.23 | 5735.44 | 9981.41 | 4361.43 |
| 3633.11 | 12060.93 | 6650.8 | 8905.71 | 9752.45 | 8959.86 |
| 11366 | 10620.25 | 5562.25 | 6085.75 | 8499.75 | 7092.4 |
| 2614 | 25148 | 4899 | 9558.5 | 6482.81 | 17290 |
| 2614 | 25148 | 4899 | 9558.5 | 6482.81 | 17290 |
| 2716.89 | 8479.87 | 6900.15 | 10891.69 | 6876.33 | 6064.18 |
| 2548 | 25081.5 | 4821 | 8890 | 5962.49 | 16761 |
| 2844.64 | 9977.3 | 4776.73 | 5501.94 | 7582.89 | 8080.93 |
| 7113.83 | 6556.75 | 9224.75 | 8873.92 | 7970.41 | 5750 |
| 7258.26 | 2521.48 | 6342.08 | 5141.59 | 8494.99 | 5072.33 |
| 1672 | 16674 | 3552.68 | 8600 | 6175.47 | 9468 |
| 1440.83 | 11790.33 | 3623.18 | 9800.16 | 5684.49 | 6783.5 |
| 1170.13 | 10516.61 | 3709 | 9936.68 | 6734.22 | 7354.6 |
| 2637.16 | 1981.66 | 2859.84 | 2002.99 | 2250.83 | 2830.5 |
| 1918 | 14966.9 | 2736.67 | 7647 | 4473.33 | 7275 |
| 3676.83 | 6059.75 | 8298.5 | 8170.67 | 5500.66 | 5045.75 |
| 3676.83 | 6058.75 | 8298.5 | 8170.67 | 5499.66 | 5045.75 |
| 1459.47 | 3323.13 | 3038.74 | 8220.27 | 5850.87 | 2593.33 |
| 899.5 | 10892 | 3199 | 7781 | 3758.5 | 11446.5 |
| 5300 | 2110.5 | 2445.67 | 315 | 4354.5 | 771 |
| 2371.33 | 1442.08 | 3573.92 | 3383.41 | 2755.25 | 2323.5 |
| 981.5 | 9161.75 | 2420.76 | 5229.25 | 2840.41 | 5860.5 |
| 3307.33 | 2932.23 | 5020.83 | 3340.17 | 2189.33 | 4015.5 |
| 2809.83 | 2355.08 | 3607.75 | 3296.41 | 2809.75 | 2818.5 |
| 955.5 | 8537.25 | 2304.42 | 3865.75 | 2064.58 | 5554.5 |
| 1423.67 | 1592.13 | 2513 | 625.67 | 3522 | 4434.67 |
| 2179.83 | 1379.08 | 3463.09 | 3164.91 | 1679.75 | 2309 |
| 2179.83 | 1378.08 | 3464.09 | 3166.91 | 1675.75 | 2308.5 |
| 978.5 | 8538.25 | 2320.42 | 3968.25 | 2072.24 | 5548.5 |
| 1060.33 | 1837 | 1706.17 | 5558.67 | 3725.31 | 905.5 |

| 872 | 8290 | 1832 | 4292 | 2105.83 | 5537 |
| --- | --- | --- | --- | --- | --- |
| 2257.83 | 1286.33 | 2563.17 | 1571.16 | 1634 | 2137.5 |
| 785 | 7750 | 1452 | 2002 | 1736 | 5265 |
| 396 | 1412 | 926.68 | 5946 | 4248.46 | 1038 |
| 2020.5 | 956 | 2315.17 | 1249.83 | 1476.16 | 1531.5 |
| 2694.5 | 831.5 | 1657.3 | 1664.75 | 1807.98 | 1076.25 |
| 3427.27 | 810.94 | 1543.51 | 1364.87 | 3023.33 | 611.2 |
| 1018.33 | 1182 | 1486.83 | 4064.67 | 2433.99 | 554.5 |
| 77 | 711 | 405.34 | 2957.5 | 2490.8 | 427.5 |
| 217.75 | 2051.4 | 1839.5 | 2696.5 | 333.25 | 1820.5 |
| 217.75 | 2051.4 | 1839.5 | 2696.5 | 333.25 | 1820.5 |
| 143.5 | 1236 | 921.67 | 3444.5 | 1645.83 | 381 |
| 612 | 970 | 577 | 846.5 | 1078.5 | 884.5 |
| 40 | 83 | 302.34 | 2276 | 1881.03 | 515 |
| 216.14 | 322.8 | 698.06 | 839.94 | 992.66 | 140.6 |
| 518 | 50 | 849 | 56 | 948 | 1398 |
| 559.67 | 672 | 375.67 | 970.67 | 647 | 112.33 |
| 51 | 127 | 260 | 1973.5 | 1635.16 | 154 |
| 37 | 662.5 | 190.34 | 1928 | 1242.66 | 357 |
| 30 | 77 | 227.34 | 1735 | 1424.14 | 488 |
| 45 | 142.5 | 162 | 1201 | 1071.49 | 685 |
| 165.5 | 124 | 1.5 | 77.5 | 98 | 20 |
| 27 | 113 | 150 | 1141 | 948.66 | 64.5 |
| 14.33 | 288.33 | 184.67 | 263.33 | 83 | 594 |
| 67 | 72.5 | 78 | 668.5 | 520.32 | 534 |
| 66 | 66.5 | 78 | 668.5 | 520.32 | 529 |
| 66 | 66.5 | 78 | 668.5 | 520.32 | 529 |
| 66 | 66.5 | 78 | 668.5 | 520.32 | 529 |
| 66 | 66.5 | 78 | 668.5 | 520.32 | 529 |
| 66 | 66.5 | 78 | 668.5 | 520.32 | 529 |
| 66 | 66.5 | 78 | 668.5 | 520.32 | 529 |
| 66 | 66.5 | 78 | 668.5 | 520.32 | 529 |
| 66 | 66.5 | 78 | 668.5 | 520.32 | 529 |
| 66 | 66.5 | 78 | 668.5 | 520.32 | 529 |
| 66 | 66.5 | 78 | 668.5 | 520.32 | 529 |
| 66 | 66.5 | 78 | 668.5 | 520.32 | 529 |
| 66 | 66.5 | 78 | 668.5 | 520.32 | 529 |
| 66 | 66.5 | 78 | 668.5 | 520.32 | 529 |
| 66 | 66.5 | 78 | 668.5 | 520.32 | 529 |
| 0.012 | 1 | 0.012 | 25 | 3 | 0.012 |
| 30 | 229 | 162 | 17 | 41 | 0.012 |
| 30 | 229 | 162 | 17 | 41 | 0.012 |
| 38 | 272 | 47 | 19 | 169 | 0.012 |
| 18 | 38 | 8 | 43 | 0.33 | 39 |
| 16 | 28 | 22 | 37 | 115 | 14 |
| 16 | 28 | 22 | 37 | 115 | 14 |
| 1 | 14 | 36 | 13 | 28.5 | 2 |
| 1 | 14 | 35 | 7 | 28 | 2 |
| 1 | 14 | 35 | 7 | 28 | 2 |
| 1 | 14 | 35 | 7 | 28 | 2 |
| 1 | 14 | 35 | 7 | 28 | 2 |
| 1 | 14 | 35 | 7 | 28 | 2 |
| 1 | 14 | 35 | 7 | 28 | 2 |
| 199 | 3 | 8.67 | 6 | 4.5 | 0.012 |
| 2 | 4 | 16 | 13 | 14.83 | 1 |
| 2 | 4 | 16 | 12 | 14.83 | 1 |
| 2 | 4 | 16 | 12 | 14.83 | 1 |
| 2 | 4 | 16 | 12 | 14.83 | 1 |

| 2 | 4 | 16 | 12 | 14.83 | 1 |
| --- | --- | --- | --- | --- | --- |
| 3 | 1 | 0.012 | 1 | 0.012 | 0.012 |
| 0.012 | 1 | 0.012 | 10 | 25.32 | 0.012 |
| 0.012 | 1 | 0.012 | 3 | 1.99 | 0.012 |
| 0.012 | 3 | 117 | 176 | 1 | 6 |
| 0.012 | 0.012 | 0.012 | 1 | 0.012 | 0.012 |
| 0.012 | 0.012 | 0.012 | 1 | 1 | 0.012 |
| 0.012 | 0.012 | 0.012 | 1 | 0.012 | 0.012 |
| 0.012 | 2 | 2 | 0.012 | 1 | 10 |
| 0.012 | 2 | 0.012 | 4 | 7 | 0.012 |
| 0.012 | 0.012 | 4.02 | 1 | 0.012 | 0.012 |
| 0.012 | 0.012 | 0.012 | 0.012 | 0.012 | 0.012 |
| 0.012 | 1 | 3 | 16 | 2.5 | 0.012 |
| 0.012 | 0.012 | 1 | 6 | 0.5 | 0.012 |
| 0.012 | 0.012 | 0.012 | 4 | 0.012 | 0.012 |
| 0.012 | 1 | 0.012 | 0.012 | 1 | 0.012 |
| 0.012 | 0.012 | 0.012 | 0.012 | 0.012 | 0.012 |
| 0.012 | 0.012 | 0.012 | 0.012 | 0.012 | 0.012 |
| 0.012 | 0.012 | 0.012 | 17 | 0.012 | 0.012 |
| 0.012 | 0.012 | 0.012 | 0.012 | 0.012 | 0.012 |
| 0.012 | 0.012 | 0.012 | 17 | 0.012 | 0.012 |
| 0.012 | 0.5 | 0.012 | 0.012 | 0.012 | 0.012 |
| 0.012 | 0.012 | 0.012 | 17 | 0.012 | 0.012 |
| 0.012 | 0.012 | 0.012 | 17 | 0.012 | 0.012 |
| 0.012 | 0.012 | 0.012 | 17 | 0.012 | 0.012 |
| 0.012 | 0.012 | 0.012 | 1 | 0.012 | 0.012 |
| 0.012 | 0.012 | 0.012 | 1 | 0.012 | 0.012 |
| 0.012 | 0.012 | 0.012 | 1 | 0.012 | 0.012 |
| 0.012 | 0.012 | 1 | 0.012 | 0.012 | 0.012 |
| 0.012 | 0.012 | 0.012 | 2 | 0.012 | 0.012 |
| 0.012 | 0.012 | 0.012 | 0.012 | 0.012 | 0.012 |
| 0.012 | 0.012 | 0.012 | 0.012 | 0.012 | 0.012 |
| 0.012 | 0.012 | 0.012 | 0.012 | 0.012 | 0.012 |
| 0.012 | 0.012 | 0.012 | 1 | 0.012 | 0.012 |
| 0.012 | 0.012 | 0.012 | 0.012 | 0.012 | 0.012 |
| 0.012 | 0.012 | 0.012 | 1 | 0.012 | 0.012 |
| 0.012 | 0.012 | 0.012 | 0.012 | 0.012 | 0.012 |
| 0.012 | 0.012 | 0.012 | 0.012 | 0.012 | 0.012 |
| 0.012 | 0.012 | 0.012 | 0.012 | 0.012 | 0.012 |
| 0.012 | 0.012 | 0.012 | 1 | 0.012 | 0.012 |
| 0.012 | 0.012 | 0.012 | 1 | 0.012 | 0.012 |
| 0.012 | 0.012 | 0.012 | 4 | 0.012 | 0.012 |
| 0.012 | 0.012 | 0.012 | 0.012 | 0.012 | 0.012 |
| 0.012 | 0.012 | 0.012 | 0.012 | 0.012 | 0.012 |
| 0.012 | 0.012 | 0.012 | 0.012 | 0.012 | 0.012 |
| 0.012 | 0.012 | 0.012 | 0.012 | 0.012 | 0.012 |
| 0.012 | 0.012 | 0.012 | 0.012 | 0.012 | 0.012 |
| 0.012 | 0.012 | 0.012 | 0.012 | 0.012 | 0.012 |
| 0.012 | 0.012 | 0.012 | 0.012 | 0.012 | 0.012 |
| 0.012 | 0.012 | 0.012 | 0.012 | 0.012 | 0.012 |
| 0.012 | 0.012 | 0.012 | 0.012 | 0.012 | 0.012 |
| 0.012 | 0.012 | 0.012 | 0.012 | 0.012 | 0.012 |
| 0.012 | 0.012 | 0.012 | 0.012 | 0.012 | 0.012 |
| 0.012 | 0.012 | 0.012 | 0.012 | 0.012 | 0.012 |
| 0.012 | 0.012 | 0.012 | 0.012 | 0.012 | 0.012 |
| 0.012 | 0.012 | 0.012 | 0.012 | 0.012 | 0.012 |
| 0.012 | 0.012 | 0.012 | 0.012 | 0.012 | 0.012 |
| 0.012 | 0.012 | 0.012 | 0.012 | 0.012 | 0.012 |

| 0.012 | 0.012 | 0.012 | 0.012 | 0.012 | 0.012 |
| --- | --- | --- | --- | --- | --- |
| 0.012 | 0.012 | 0.012 | 0.012 | 0.012 | 0.012 |
| 0.012 | 0.012 | 0.012 | 0.012 | 0.012 | 0.012 |
| 0.012 | 0.012 | 0.012 | 0.012 | 0.012 | 0.012 |
| 0.012 | 0.012 | 0.012 | 0.012 | 0.012 | 0.012 |
| 0.012 | 0.012 | 0.012 | 0.012 | 0.012 | 0.012 |
| 0.012 | 0.012 | 0.012 | 0.012 | 0.012 | 0.012 |
| 0.012 | 0.012 | 0.012 | 0.012 | 0.012 | 0.012 |
| 0.012 | 0.5 | 0.012 | 0.012 | 0.012 | 0.012 |
| 0.012 | 0.012 | 0.012 | 0.012 | 0.012 | 0.012 |
| 0.012 | 0.012 | 0.012 | 0.012 | 0.012 | 0.012 |
| 0.012 | 0.012 | 0.012 | 0.012 | 0.012 | 0.012 |
| 0.012 | 0.012 | 0.012 | 0.012 | 0.012 | 0.012 |
| 0.012 | 0.012 | 0.012 | 0.012 | 0.012 | 0.012 |
| 0.012 | 0.012 | 0.012 | 0.012 | 0.012 | 0.012 |
| 0.012 | 0.012 | 0.012 | 0.012 | 0.012 | 0.012 |
| 0.012 | 0.012 | 0.012 | 0.012 | 0.012 | 0.012 |
| 0.012 | 0.012 | 0.012 | 0.012 | 0.012 | 0.012 |

| EAC_13 | EAC_14 | EAC_15 | EAC_16 | EAC_17 | EAC_3 |
| --- | --- | --- | --- | --- | --- |
| 9615312.16 | 8847819.85 | 8817801.76 | 8687072.05 | 8393572.57 | 8362063.7 |
| 4371351.76 | 4238028.27 | 4092471.87 | 4065224.38 | 4008586.96 | 3932980.85 |
| 2536153.23 | 2181503.06 | 2062753.26 | 2030089.31 | 1883454.66 | 1888888.79 |
| 1762769.88 | 1804832.36 | 1661692.35 | 1749171.95 | 1596980.41 | 1614752.16 |
| 1421265.86 | 1348606.39 | 1347221.9 | 1269278.02 | 1237007.06 | 1211413.05 |
| 1181990.7 | 1206267.87 | 1293308.26 | 1224570.46 | 1204669.43 | 1199773.02 |
| 1456882.82 | 1188752.59 | 1092373.9 | 1094128.78 | 730961.02 | 849683.18 |
| 886847.58 | 833415.35 | 838377.12 | 805179.38 | 789994.52 | 792809.45 |
| 849764.4 | 688665.13 | 633221.72 | 679407.05 | 533365.52 | 595465.58 |
| 615547.39 | 591438.75 | 646515.64 | 602346.55 | 629623.54 | 608559.69 |
| 544217.47 | 549711.45 | 582970.11 | 564756.09 | 537710.32 | 545579.53 |
| 526586.41 | 514094.53 | 557240.71 | 505596.94 | 560980.66 | 515977.46 |
| 527872.21 | 519219.23 | 539437.3 | 499381.02 | 563911.89 | 510233.61 |
| 516946.28 | 493678.07 | 492631.48 | 518236.66 | 522306.77 | 517245.22 |
| 523245.07 | 512956.34 | 462684.12 | 452311.88 | 520745.15 | 481165.06 |
| 871897.82 | 602994.65 | 481312.46 | 493646.17 | 380227.91 | 455301.49 |
| 559109.68 | 516918.49 | 504389.55 | 487418.18 | 451042.51 | 452283.06 |
| 453043.4 | 468371.74 | 499846.64 | 472533.07 | 463962.6 | 457555.84 |
| 468716.83 | 436450.75 | 436488.18 | 414959.35 | 438313.28 | 409852.45 |
| 403021.11 | 397366.22 | 404288.18 | 371349.64 | 474926.95 | 389736.15 |
| 416578.96 | 413338.02 | 425579.97 | 412528.54 | 416471.07 | 421833.65 |
| 436648.68 | 421952.93 | 437649.7 | 412156.2 | 429196.83 | 421607.27 |
| 397827.64 | 399652.48 | 416569.81 | 403560.66 | 396906.1 | 389071.6 |
| 388296.33 | 387654.66 | 468915.26 | 510597.87 | 317514.49 | 402303.81 |
| 390249.55 | 377618.3 | 372683.92 | 369872.72 | 401154.46 | 378394.14 |
| 360033.45 | 380768.77 | 342955.61 | 365110.83 | 342427.04 | 349234.47 |
| 355583.33 | 361135.63 | 355938.51 | 353555.04 | 309642.28 | 324413.77 |
| 329046.56 | 334401.65 | 347282.77 | 335156.69 | 320924.52 | 319965.63 |
| 356813.91 | 346678.4 | 350928.67 | 318195.73 | 330230.06 | 306526.1 |
| 334168.43 | 324306.29 | 320758.77 | 290903.08 | 329181.36 | 308039 |
| 392259.08 | 369199.53 | 329000.31 | 327169.74 | 273327.85 | 271880.75 |
| 299606.59 | 308240.33 | 317902.29 | 306190.55 | 275218.28 | 281785.88 |
| 421664.04 | 337585.39 | 287914.17 | 294678.99 | 288545.81 | 299399.65 |
| 270277.12 | 261261.6 | 291200.61 | 270036.43 | 327365.55 | 294862.34 |
| 336663.41 | 341791.27 | 287261.63 | 316644.13 | 231157.03 | 254331.99 |
| 263868.76 | 278270.1 | 289902.68 | 279319.09 | 270768.77 | 264446.33 |
| 298465.99 | 282751.97 | 284446 | 283589.33 | 247097.97 | 276857.98 |
| 261929.43 | 262026.85 | 301607.58 | 306833.88 | 286788.6 | 274959.06 |
| 270186.04 | 269737.24 | 290795.64 | 278278.37 | 252960.64 | 262109.09 |
| 269474.37 | 269438.14 | 266787.23 | 267104.63 | 266752.61 | 257259.55 |
| 247316.92 | 250030.84 | 263798.6 | 254387.89 | 289076.96 | 266848.35 |
| 380860.29 | 306647.72 | 300085.3 | 280475.42 | 241520.26 | 225213.22 |
| 276102.72 | 277845.01 | 257970.09 | 266242.21 | 243362.7 | 256007.86 |
| 231038.98 | 233516.22 | 242246.61 | 233251.96 | 294079.35 | 254773.36 |
| 337264.08 | 270012.47 | 275284.73 | 276793.01 | 242161.22 | 262251.07 |
| 342409.51 | 293032.93 | 269758.93 | 285212.56 | 212204.35 | 241953.92 |
| 313399.78 | 285454.92 | 282504.07 | 270201.33 | 240445.05 | 222936.16 |
| 254786.8 | 235855.81 | 255164.83 | 248597.71 | 242221.82 | 248088.27 |
| 243407.8 | 240566.97 | 255532.68 | 243217.23 | 244881.12 | 234770.68 |
| 286074.74 | 249361.16 | 244596.49 | 231103.9 | 219320.32 | 215853.45 |
| 264445.32 | 231146.02 | 237439.77 | 209010.17 | 251720.33 | 225116.78 |
| 244080.2 | 232933.46 | 210110.58 | 220639.58 | 220858.6 | 219604 |
| 210830.36 | 198182.08 | 223544.47 | 200203.99 | 210193.67 | 205290.9 |
| 213710.67 | 205509.74 | 207378.31 | 194368.38 | 216238.06 | 205516.33 |
| 183636.81 | 185533.47 | 196723.11 | 187851.2 | 197358.78 | 193351.68 |
| 210639.65 | 213683.23 | 196067.44 | 200218.68 | 156872.82 | 166124.46 |
| 223569.55 | 181899.58 | 170844.03 | 182437.43 | 184398.98 | 194274.71 |

| 212707.63 | 215388.98 | 172875.84 | 202121.69 | 120472.79 | 153661.4 |
| --- | --- | --- | --- | --- | --- |
| 160134.32 | 167784.26 | 156704.06 | 171785.76 | 180694.71 | 180262.1 |
| 190860.07 | 168638.84 | 180763.82 | 160738.28 | 180894.43 | 172315.19 |
| 229715.36 | 210894.18 | 217839.47 | 200848.57 | 126418.95 | 138589.53 |
| 189040.03 | 181603.39 | 148737.82 | 163735.74 | 159440.29 | 158126.79 |
| 166075.57 | 165988.08 | 178028.44 | 172295.42 | 148635.29 | 149976.14 |
| 228176.62 | 180663.48 | 158971.5 | 165614.82 | 134023.9 | 128222.46 |
| 215100.67 | 170761.26 | 145484.42 | 153823.69 | 145328.28 | 144492.4 |
| 129369.19 | 128692.99 | 148438.56 | 132885.37 | 131465.89 | 130345.62 |
| 117308.07 | 125404.5 | 119267.2 | 111893.8 | 146316.17 | 120181.78 |
| 214900.35 | 163033.45 | 131840.25 | 124745.53 | 108164.69 | 114471.62 |
| 154090.8 | 139847.43 | 106120.54 | 133469.21 | 133123.08 | 130198.28 |
| 147563.11 | 126580.1 | 112995.61 | 113585.33 | 121214.18 | 121563.39 |
| 112363.77 | 121413.81 | 131249.21 | 126669.8 | 108230.33 | 112652.36 |
| 161332.26 | 140612.18 | 125098.74 | 120239.67 | 110582.6 | 106794.91 |
| 114003.97 | 113541.91 | 127520.01 | 112217.19 | 142010.67 | 114317.03 |
| 163269.35 | 131391.94 | 131400.6 | 106468.34 | 109999.02 | 95184.88 |
| 131900.81 | 130929.87 | 102302 | 122029.51 | 87157.17 | 107611.22 |
| 120599.15 | 108735.47 | 96086.4 | 106831.66 | 114197.69 | 121971.35 |
| 123240.37 | 117047.11 | 117438.28 | 108548.48 | 112002.85 | 103223.56 |
| 102767.82 | 106797.09 | 109280.76 | 104006.12 | 120344.56 | 108578.87 |
| 175936.74 | 125248.83 | 111927.46 | 114225.99 | 106378.88 | 104857.77 |
| 108392.91 | 111529.48 | 120717.94 | 109824.47 | 96062.75 | 94139.02 |
| 139171.45 | 125154.3 | 128530.13 | 111178.7 | 65769.35 | 81584.4 |
| 215332.38 | 131600.87 | 113982.89 | 115994.19 | 70677.18 | 94639.91 |
| 64687.75 | 66448.45 | 108360.07 | 71086.86 | 157600.62 | 102036.6 |
| 134600.53 | 112528.97 | 110646.4 | 98365.23 | 82070.74 | 85006.75 |
| 95253.94 | 96543.8 | 102947.08 | 97121.24 | 72319.95 | 78853.53 |
| 86297.58 | 86177.19 | 95289.55 | 81288.99 | 93046.32 | 84401.3 |
| 87716.74 | 88714.19 | 91726.9 | 89886.16 | 81815.07 | 83812.77 |
| 118586.42 | 92610.74 | 90838.67 | 76781.53 | 88160.81 | 74629.96 |
| 69838.55 | 73398.16 | 73960.35 | 73073.99 | 84712.04 | 79214.36 |
| 78033.57 | 76507.7 | 81179.28 | 81909.81 | 71900.47 | 76818.09 |
| 132884.99 | 85693.01 | 82534.46 | 82235.87 | 58090.1 | 63536.6 |
| 95607.82 | 75888.32 | 77098.92 | 72558.51 | 82760.96 | 72211.47 |
| 158072.81 | 90724.32 | 73273.36 | 67075.44 | 72526.77 | 64241.89 |
| 63023.32 | 68131.04 | 73517.43 | 63807.3 | 84886.2 | 63759.77 |
| 93817.1 | 66249.79 | 64419.45 | 55032.09 | 89926.03 | 69550.88 |
| 150781.35 | 93683.86 | 59531.42 | 65391.69 | 68492.82 | 60183.86 |
| 81024.52 | 72139.57 | 69211.38 | 70184.61 | 61302 | 66234.41 |
| 74808.24 | 70074.24 | 79669.91 | 73274.86 | 70245.4 | 66255.64 |
| 162950.79 | 87169.79 | 59051.67 | 70279.18 | 63000.3 | 65249.52 |
| 73846.91 | 69541.74 | 65225.26 | 66066.6 | 65831.09 | 65439.36 |
| 80708.66 | 67128.52 | 67983.64 | 68489.23 | 55829.29 | 59589.46 |
| 87307.03 | 76316.39 | 71641.61 | 64692.33 | 62092.05 | 57469.06 |
| 42477.19 | 41793.3 | 68859.26 | 43082.15 | 106176.15 | 67992.53 |
| 155997.77 | 94788.4 | 69391.23 | 62336.24 | 40451.83 | 46671.49 |
| 56716.12 | 57640.09 | 61439.86 | 60984.26 | 53037.69 | 59272.5 |
| 62570.74 | 60975.65 | 51812 | 60531.99 | 62671.42 | 59166.83 |
| 83329.08 | 66728.4 | 65402.92 | 60739.28 | 55342.3 | 53564.01 |
| 60343.58 | 57408.95 | 59591.49 | 54821.97 | 59801.19 | 57564.19 |
| 69098.5 | 53203.09 | 66360.44 | 61300.55 | 57501.68 | 54739.79 |
| 34664.16 | 33879.1 | 51836.76 | 37337.86 | 98000.46 | 63915.83 |
| 50994.48 | 42344.97 | 49991.56 | 41800.79 | 68773.15 | 53430.5 |
| 47582.6 | 51154.77 | 47601.46 | 45329.31 | 54183.33 | 48692.52 |
| 65587.23 | 51023.06 | 43153.11 | 39339.98 | 59566.83 | 46998.27 |
| 53074.69 | 51731.73 | 36552.92 | 39756.48 | 51367.43 | 50302.4 |
| 49643.04 | 44683.83 | 39320.62 | 45171.57 | 56284.87 | 48018.95 |

| 65203.66 | 50136.78 | 49010.33 | 40081.18 | 50782.43 | 41976.05 |
| --- | --- | --- | --- | --- | --- |
| 48417.3 | 37774.98 | 38814.61 | 34744.15 | 57923.83 | 46081.27 |
| 95087.04 | 72922.09 | 59855.29 | 48473.32 | 29645.95 | 36546.05 |
| 70202.81 | 56354.93 | 59567.88 | 46354.62 | 33781.03 | 32716.49 |
| 42931.03 | 39402.71 | 51122.6 | 46634.83 | 48912.2 | 41562.41 |
| 68907.71 | 49771.51 | 49436.41 | 50144.57 | 44866.04 | 46185.6 |
| 80808.56 | 47089.66 | 47392.85 | 41866.25 | 39254.31 | 41759.32 |
| 136048.59 | 57608.99 | 46494.45 | 47200.01 | 33584.65 | 36244.57 |
| 52766.26 | 53191.1 | 45693.46 | 42154.06 | 31745.43 | 32414.41 |
| 41947.83 | 36499.09 | 41068.26 | 39403.19 | 43184.71 | 37622.26 |
| 48310.82 | 46202.07 | 41770.34 | 40993.09 | 32022.93 | 30245.29 |
| 30531.09 | 40784.32 | 42877.32 | 43741.6 | 37710.69 | 36039.2 |
| 41057.38 | 43389.35 | 39440.97 | 41412.94 | 32333.93 | 34406.06 |
| 157157.66 | 63770.08 | 33852.46 | 50173.79 | 16311.22 | 43470.88 |
| 32422.39 | 35094.34 | 36137.71 | 31388.93 | 41299.1 | 32162.72 |
| 46787.85 | 47929.6 | 39267.68 | 43109.41 | 20084.92 | 32356.57 |
| 30440.46 | 29369.54 | 32654.84 | 33150.48 | 39514.2 | 35825.34 |
| 48103.95 | 44911.28 | 34496.31 | 32649.03 | 28208.2 | 28105.23 |
| 124364.38 | 63625.02 | 25193.99 | 33670.75 | 21107.5 | 27887.31 |
| 29871.62 | 28972.37 | 31082.51 | 31094.48 | 39365.88 | 34877.34 |
| 30623.94 | 27968.57 | 42773.23 | 31342.68 | 50384.82 | 37653.36 |
| 34675.97 | 35485.1 | 34624.26 | 29361.76 | 32017.62 | 25853.97 |
| 30653.3 | 28504.2 | 28658.72 | 23951.08 | 38864.12 | 30852.69 |
| 113350.37 | 52411.59 | 26797.58 | 38597.54 | 22404.5 | 25668.95 |
| 17800.65 | 17397.84 | 34911.27 | 19441 | 55640.82 | 33169.57 |
| 25945.98 | 25608.43 | 31224.4 | 26719.09 | 34681.88 | 30573.93 |
| 35100.43 | 30998.78 | 32925.3 | 29960.85 | 26815.88 | 27083.52 |
| 37300.64 | 37596.11 | 37144.72 | 27422.1 | 25593.87 | 22218.26 |
| 34252.68 | 31819.22 | 28924.69 | 27387.65 | 26062.38 | 26420.03 |
| 23934.01 | 21754.69 | 29519.8 | 26039.34 | 34628.04 | 31548.33 |
| 26671.7 | 25590.48 | 27141.15 | 30225.21 | 33793.49 | 30137.3 |
| 24387.23 | 25797.74 | 39915.63 | 50865.97 | 18429.5 | 29908.8 |
| 32852.62 | 36137.1 | 25483.48 | 30168.78 | 19755.76 | 24696.9 |
| 31914.03 | 30979.53 | 28582.31 | 26933.03 | 24389.2 | 24810.23 |
| 29226.95 | 32862.9 | 26495.35 | 28915.75 | 25121.95 | 27423.73 |
| 39311.24 | 24537 | 26019.18 | 18261.07 | 38906.05 | 28100.65 |
| 59161.72 | 34118.32 | 29190.99 | 25607.41 | 21010.05 | 22175.78 |
| 22846.39 | 23269.8 | 25983.4 | 25310.89 | 22729.2 | 23564.03 |
| 26004.06 | 17106.53 | 20262.26 | 15743.82 | 38286.42 | 28864.82 |
| 52685.75 | 35923.46 | 23752.09 | 27025.12 | 14314.71 | 21183.88 |
| 38295.05 | 32390.08 | 34403.39 | 26367.81 | 12956.38 | 15958.4 |
| 30735.83 | 25972.23 | 34619.17 | 25213.69 | 20710.6 | 18355.49 |
| 21977.57 | 15571.81 | 19824.39 | 15802.27 | 38825.55 | 27321.82 |
| 21779.64 | 22718.4 | 22826.44 | 23317.58 | 22911.38 | 22694.94 |
| 22007.48 | 22536.96 | 24258.07 | 23074.39 | 22570.38 | 22517.03 |
| 21681.89 | 22656.4 | 22822.69 | 23072.58 | 22715.88 | 22563.19 |
| 21669.89 | 22632.4 | 22819.69 | 23036.58 | 22713.88 | 22552.19 |
| 102685.18 | 44585.84 | 18252.79 | 20657.66 | 7141.65 | 12570.15 |
| 18852.73 | 19725.99 | 18907.23 | 17300.01 | 24689.37 | 20317.76 |
| 34293.48 | 30058.41 | 32666.39 | 25073.81 | 10760.38 | 13562.4 |
| 13235.01 | 13499.26 | 29487.11 | 16008.09 | 34933 | 19766.63 |
| 13235.01 | 13499.26 | 29487.11 | 16008.09 | 34933 | 19765.63 |
| 21284.86 | 19938.17 | 20753.19 | 18621.09 | 20861.55 | 17337.11 |
| 47563.3 | 29094.76 | 14419.13 | 16044.32 | 13970.43 | 11234.53 |
| 38581.46 | 20340.59 | 16752.3 | 17938.22 | 19510.22 | 20011.45 |
| 22922.49 | 18352.97 | 23983.52 | 19337.71 | 18348.55 | 18009.03 |
| 20424.84 | 16117.29 | 19467.9 | 17650.66 | 20037.71 | 19583.94 |
| 14541.62 | 15211.55 | 14256.56 | 14843.36 | 20333.29 | 15869.75 |

| 16440.68 | 11496.64 | 16904.36 | 13093.95 | 26163.32 | 19988.8 |
| --- | --- | --- | --- | --- | --- |
| 21995.82 | 20864.22 | 24872.27 | 20596.96 | 9408.88 | 12071.4 |
| 28006.56 | 27197.27 | 20877.2 | 25398.95 | 6057.16 | 18089.5 |
| 9191.73 | 11775.72 | 16741.58 | 17644.95 | 17128.66 | 17582.57 |
| 15816.22 | 8710.97 | 15354.45 | 7085.77 | 31839 | 19489.8 |
| 12880.31 | 10560.8 | 10149.17 | 12241.61 | 16502 | 16482.4 |
| 35053.71 | 24067.11 | 10827.93 | 12382.94 | 12206.78 | 8811.63 |
| 19619.24 | 17068.9 | 13812.7 | 13109.63 | 5165.79 | 13243.6 |
| 7957.5 | 7244.6 | 17579.66 | 10621.9 | 24390 | 16180.8 |
| 46495.67 | 22577.01 | 12910.71 | 13403.16 | 5534.42 | 7325.57 |
| 12577.08 | 8337.33 | 15126.7 | 10651.09 | 15754 | 12990.23 |
| 45407.64 | 21206.74 | 13981.47 | 14332.95 | 6840.38 | 9964.38 |
| 40622.04 | 24427.49 | 8931.21 | 8899.89 | 7542.23 | 5170.33 |
| 40582.95 | 24320.57 | 8926.21 | 8877.89 | 7542.23 | 5132.33 |
| 5652.67 | 6247.32 | 7633.52 | 7189.55 | 20752.05 | 12735.95 |
| 5542.92 | 6161.32 | 7626.77 | 6908.55 | 20554.55 | 12593.2 |
| 13393.99 | 12015.55 | 6478 | 6088.53 | 14562.32 | 10374.9 |
| 9407.59 | 10873.83 | 6477.91 | 10390.4 | 4625.22 | 11070.05 |
| 13714.09 | 14157.94 | 8233.11 | 8766.26 | 9654.73 | 5067.33 |
| 14729.95 | 12718.15 | 9064.57 | 12711.14 | 5419.33 | 9810.25 |
| 8571.51 | 5981.66 | 13386.7 | 9324.09 | 13556 | 10584.23 |
| 8571.51 | 5981.66 | 13386.7 | 9324.09 | 13556 | 10584.23 |
| 8567.51 | 5981.66 | 13386.7 | 9324.09 | 13556 | 10584.23 |
| 8567.51 | 5981.66 | 13386.7 | 9324.09 | 13556 | 10584.23 |
| 8567.51 | 5981.66 | 13386.7 | 9324.09 | 13556 | 10584.23 |
| 8567.51 | 5981.66 | 13386.7 | 9324.09 | 13556 | 10584.23 |
| 12056.91 | 11791.86 | 9771.54 | 11856.27 | 5728.33 | 9320.25 |
| 12052.91 | 11791.86 | 9771.54 | 11856.27 | 5728.33 | 9320.25 |
| 11342.41 | 8601.33 | 5449.32 | 6239.02 | 16971.55 | 10600.3 |
| 27507.18 | 15584.46 | 8188.93 | 11721.7 | 4447.75 | 9462.4 |
| 7400.57 | 6376.3 | 4643.5 | 5065.95 | 13453 | 9244.4 |
| 21216.1 | 15452.25 | 6096 | 5891 | 3833 | 3323 |
| 21216.1 | 15451.25 | 6096 | 5891 | 3833 | 3323 |
| 10563.06 | 10005.91 | 12116.57 | 10237.44 | 2702.38 | 5206.63 |
| 16202.43 | 13914.75 | 5913 | 5700 | 3819 | 3295 |
| 19251.03 | 11095.56 | 3991.8 | 8546.97 | 3221.05 | 7828.63 |
| 7769.93 | 7381.77 | 10204.12 | 6513.61 | 4767.5 | 4970.83 |
| 15927.79 | 10159.46 | 6268.29 | 8672.62 | 2874.98 | 8768.25 |
| 32814.95 | 15821.57 | 4547.72 | 4033.9 | 2531 | 2187.2 |
| 18987.75 | 11992.49 | 7341.86 | 4868.07 | 1443 | 2311 |
| 33532.46 | 14825.3 | 5849.75 | 4236.05 | 1711.72 | 2243.3 |
| 2238.85 | 2507.75 | 809.5 | 1993.5 | 2368.5 | 3347 |
| 13400.26 | 10580.94 | 4277.12 | 5261.69 | 2725 | 2542 |
| 7112.93 | 7029.52 | 9672.62 | 5742.11 | 3955 | 2337.33 |
| 7102.18 | 7007.27 | 9672.62 | 5741.11 | 3955 | 2337.33 |
| 37338.89 | 12545.54 | 6082.01 | 4540.28 | 1533.85 | 2730.67 |
| 12301.66 | 9194.19 | 7794.12 | 4476.85 | 1351.5 | 1491 |
| 1502.48 | 785.62 | 967.62 | 537.12 | 11220.5 | 5709 |
| 11390.74 | 4031.34 | 2405.5 | 2614.08 | 2231.5 | 2432.5 |
| 12861.26 | 7129.24 | 5109.69 | 3836.65 | 1355.5 | 1139.5 |
| 7146.87 | 4277.2 | 4254.87 | 2733.29 | 3443.32 | 1570.78 |
| 2194.75 | 2490.5 | 2686.5 | 2739.95 | 2333 | 3213 |
| 5454.74 | 5004.69 | 3906.45 | 3623.94 | 1333.5 | 1126.5 |
| 1793.17 | 2529.17 | 2041 | 5108 | 1647 | 5253.67 |
| 1583.24 | 2173.69 | 2600.12 | 2888.44 | 2177 | 2144.7 |
| 1579.49 | 2168.44 | 2598.12 | 2888.44 | 2177 | 2143.7 |
| 5538.09 | 5041.43 | 3912.69 | 3621.18 | 1333.5 | 1148.5 |
| 26371.93 | 7759.87 | 3524.31 | 2067.18 | 748 | 884.83 |

| 5654.04 | 5083 | 2361.5 | 2486.65 | 1462 | 1169 |
| --- | --- | --- | --- | --- | --- |
| 1584.4 | 1834 | 638.5 | 1255 | 2196 | 2551 |
| 2889 | 3716 | 919 | 1799 | 1264 | 1070 |
| 41516.97 | 11126.72 | 3117.6 | 915.71 | 187.5 | 186 |
| 1300.41 | 1437.69 | 486.62 | 1073.19 | 2186.5 | 2281 |
| 11141.6 | 3625.78 | 923.47 | 2386.61 | 1077.5 | 1225.4 |
| 1163.85 | 1081.27 | 1627.07 | 1973.6 | 854.13 | 3076.53 |
| 14050.74 | 4208.12 | 2229.95 | 1673.18 | 745.5 | 833.83 |
| 24460.43 | 6686.37 | 1660.48 | 593.62 | 3 | 87.5 |
| 616 | 630.25 | 3245.5 | 3628.75 | 194 | 817.25 |
| 616 | 630.25 | 3245.5 | 3628.75 | 194 | 817.25 |
| 5452.51 | 3209.69 | 2121.12 | 1365.89 | 48.5 | 386.33 |
| 1813.84 | 1292.5 | 536.33 | 776.53 | 200.5 | 835.83 |
| 19388.43 | 4645.86 | 458.6 | 187.74 | 0.012 | 21 |
| 987.74 | 1062.14 | 222.54 | 535.8 | 347.74 | 1097.26 |
| 43 | 540 | 32 | 547 | 906 | 2319 |
| 1002.84 | 1111.69 | 366.45 | 853.36 | 292.33 | 648.67 |
| 16834.77 | 4551.24 | 478.24 | 165.24 | 0.012 | 21 |
| 12218.52 | 3412.05 | 1340.24 | 283.71 | 21 | 19 |
| 14646.75 | 3629.85 | 357.96 | 188.1 | 0.012 | 14 |
| 9819.57 | 2771.06 | 269.09 | 357.3 | 4.5 | 11 |
| 162.07 | 153 | 11 | 149 | 64 | 333 |
| 9649.17 | 2432.5 | 267 | 119 | 2 | 12 |
| 239.4 | 159 | 139 | 82 | 6 | 50 |
| 5013.84 | 1521.5 | 183 | 191 | 17 | 31 |
| 5017.84 | 1521.5 | 183 | 191 | 14 | 28 |
| 5017.84 | 1521.5 | 183 | 191 | 14 | 28 |
| 5017.84 | 1521.5 | 183 | 191 | 14 | 28 |
| 5017.84 | 1521.5 | 183 | 191 | 14 | 28 |
| 5013.84 | 1521.5 | 183 | 191 | 14 | 28 |
| 5013.84 | 1521.5 | 183 | 191 | 14 | 28 |
| 5013.84 | 1521.5 | 183 | 191 | 14 | 28 |
| 5013.84 | 1521.5 | 183 | 191 | 14 | 28 |
| 5013.84 | 1521.5 | 183 | 191 | 14 | 28 |
| 5013.84 | 1521.5 | 183 | 191 | 14 | 28 |
| 5013.84 | 1521.5 | 183 | 191 | 14 | 28 |
| 5013.84 | 1521.5 | 183 | 191 | 14 | 28 |
| 5013.84 | 1521.5 | 183 | 191 | 14 | 28 |
| 5013.84 | 1521.5 | 183 | 191 | 14 | 28 |
| 96.99 | 116 | 21 | 448 | 0.012 | 0.012 |
| 86 | 83 | 591 | 181 | 80 | 40 |
| 86 | 83 | 591 | 181 | 80 | 40 |
| 84.01 | 53 | 53 | 32.33 | 2 | 100 |
| 15.17 | 257 | 20 | 49 | 9 | 16 |
| 62 | 5 | 85 | 13 | 8 | 10 |
| 62 | 5 | 85 | 13 | 8 | 10 |
| 21 | 66 | 3 | 33 | 2 | 10 |
| 12 | 24 | 3 | 33 | 2 | 10 |
| 12 | 24 | 3 | 33 | 2 | 10 |
| 8 | 24 | 3 | 33 | 2 | 10 |
| 8 | 24 | 3 | 33 | 2 | 10 |
| 8 | 24 | 3 | 33 | 2 | 10 |
| 8 | 24 | 3 | 33 | 2 | 10 |
| 23.42 | 76.5 | 6 | 15.2 | 1 | 36 |
| 52.84 | 132.17 | 6 | 23 | 0.012 | 38 |
| 53.84 | 132.17 | 5 | 23 | 0.012 | 38 |
| 49.84 | 132.17 | 5 | 23 | 0.012 | 38 |
| 49.84 | 132.17 | 5 | 23 | 0.012 | 38 |

| 49.84 | 131.17 | 5 | 23 | 0.012 | 38 |
| --- | --- | --- | --- | --- | --- |
| 10.4 | 16 | 2 | 16 | 1 | 0.012 |
| 18 | 156 | 7 | 4 | 0.012 | 4 |
| 28.59 | 14 | 2 | 17 | 0.012 | 4 |
| 11.4 | 8 | 2 | 4 | 0.012 | 0.012 |
| 47.67 | 10 | 1 | 16.33 | 0.012 | 0.012 |
| 9 | 12 | 1 | 16 | 0.012 | 0.012 |
| 1 | 2 | 1 | 16 | 0.012 | 0.012 |
| 1 | 10 | 0.012 | 0.012 | 1 | 2 |
| 23 | 0.012 | 15 | 7 | 0.012 | 7 |
| 33.02 | 16 | 1 | 0.012 | 0.012 | 0.012 |
| 0.012 | 12 | 0.012 | 0.012 | 0.012 | 0.012 |
| 8 | 18 | 0.012 | 0.012 | 0.012 | 0.012 |
| 13 | 42 | 0.012 | 0.012 | 0.012 | 0.012 |
| 11 | 38 | 0.012 | 0.012 | 1 | 0.012 |
| 14.75 | 22.25 | 0.012 | 1 | 0.012 | 0.012 |
| 30 | 0.012 | 0.012 | 0.012 | 2 | 0.012 |
| 4 | 15 | 0.012 | 0.012 | 0.012 | 0.012 |
| 4 | 0.012 | 0.012 | 3 | 0.012 | 1 |
| 5 | 18 | 0.012 | 0.012 | 0.012 | 0.012 |
| 0.012 | 0.012 | 0.012 | 3 | 0.012 | 1 |
| 4 | 15 | 0.5 | 0.5 | 0.012 | 0.012 |
| 0.012 | 0.012 | 0.012 | 3 | 0.012 | 1 |
| 0.012 | 0.012 | 0.012 | 3 | 0.012 | 1 |
| 0.012 | 0.012 | 0.012 | 3 | 0.012 | 1 |
| 1 | 12 | 1 | 0.012 | 0.012 | 0.012 |
| 13 | 0.012 | 1 | 0.012 | 0.012 | 0.012 |
| 7 | 4 | 1 | 0.012 | 0.012 | 0.012 |
| 8 | 0.012 | 0.012 | 0.012 | 0.012 | 0.012 |
| 0.012 | 0.012 | 0.012 | 0.012 | 0.012 | 0.012 |
| 0.012 | 1 | 0.012 | 0.012 | 0.012 | 0.012 |
| 6 | 0.012 | 0.012 | 0.012 | 0.012 | 0.012 |
| 4.25 | 0.25 | 0.012 | 0.012 | 0.012 | 0.012 |
| 5 | 0.012 | 1 | 0.012 | 0.012 | 0.012 |
| 4 | 0.012 | 0.012 | 0.012 | 0.012 | 0.012 |
| 1 | 0.012 | 1 | 0.012 | 0.012 | 0.012 |
| 0.17 | 0.012 | 0.012 | 0.012 | 0.012 | 0.012 |
| 4 | 1 | 0.012 | 0.012 | 0.012 | 0.012 |
| 4 | 0.012 | 0.012 | 0.012 | 0.012 | 0.012 |
| 1 | 0.012 | 1 | 0.012 | 0.012 | 0.012 |
| 1 | 0.012 | 1 | 0.012 | 0.012 | 0.012 |
| 0.012 | 0.012 | 0.012 | 0.012 | 0.012 | 0.012 |
| 4 | 0.012 | 0.012 | 0.012 | 0.012 | 0.012 |
| 4 | 0.012 | 0.012 | 0.012 | 0.012 | 0.012 |
| 4 | 0.012 | 0.012 | 0.012 | 0.012 | 0.012 |
| 4 | 0.012 | 0.012 | 0.012 | 0.012 | 0.012 |
| 4 | 0.012 | 0.012 | 0.012 | 0.012 | 0.012 |
| 4 | 0.012 | 0.012 | 0.012 | 0.012 | 0.012 |
| 4 | 0.012 | 0.012 | 0.012 | 0.012 | 0.012 |
| 4 | 0.012 | 0.012 | 0.012 | 0.012 | 0.012 |
| 4 | 0.012 | 0.012 | 0.012 | 0.012 | 0.012 |
| 4 | 0.012 | 0.012 | 0.012 | 0.012 | 0.012 |
| 4 | 0.012 | 0.012 | 0.012 | 0.012 | 0.012 |
| 4 | 0.012 | 0.012 | 0.012 | 0.012 | 0.012 |
| 4 | 0.012 | 0.012 | 0.012 | 0.012 | 0.012 |
| 4 | 0.012 | 0.012 | 0.012 | 0.012 | 0.012 |
| 4 | 0.012 | 0.012 | 0.012 | 0.012 | 0.012 |
| 4 | 0.012 | 0.012 | 0.012 | 0.012 | 0.012 |

| 4 | 0.012 | 0.012 | 0.012 | 0.012 | 0.012 |
| --- | --- | --- | --- | --- | --- |
| 4 | 0.012 | 0.012 | 0.012 | 0.012 | 0.012 |
| 4 | 0.012 | 0.012 | 0.012 | 0.012 | 0.012 |
| 4 | 0.012 | 0.012 | 0.012 | 0.012 | 0.012 |
| 4 | 0.012 | 0.012 | 0.012 | 0.012 | 0.012 |
| 4 | 0.012 | 0.012 | 0.012 | 0.012 | 0.012 |
| 4 | 0.012 | 0.012 | 0.012 | 0.012 | 0.012 |
| 4 | 0.012 | 0.012 | 0.012 | 0.012 | 0.012 |
| 0.012 | 0.012 | 0.5 | 0.5 | 0.012 | 0.012 |
| 0.012 | 0.012 | 0.012 | 0.012 | 0.012 | 0.012 |
| 0.012 | 0.012 | 0.012 | 0.012 | 0.012 | 0.012 |
| 0.012 | 0.012 | 0.012 | 0.012 | 0.012 | 0.012 |
| 0.012 | 0.012 | 0.012 | 0.012 | 0.012 | 0.012 |
| 0.012 | 0.012 | 0.012 | 0.012 | 0.012 | 0.012 |
| 0.012 | 0.012 | 0.012 | 0.012 | 0.012 | 0.012 |
| 0.5 | 0.012 | 0.012 | 0.012 | 0.012 | 0.012 |
| 0.012 | 0.012 | 0.012 | 0.012 | 0.012 | 0.012 |
| 0.012 | 0.012 | 0.012 | 0.2 | 0.012 | 0.012 |

| EAC_18 | EAC_19 | EAC_22 | EAC_23 | EAC_4 | EAC_24 |
| --- | --- | --- | --- | --- | --- |
| 9000045.05 | 9523223.32 | 8483802.95 | 8943580.15 | 8293132.42 | 8535516.34 |
| 4218838.11 | 4610817.17 | 4069290.85 | 4108855.33 | 3774471.13 | 4051843.3 |
| 2087618.03 | 2251829.75 | 1988266.15 | 2061115.15 | 1813841.97 | 2008195.59 |
| 1770185.58 | 1896734.06 | 1843749.24 | 1733309.66 | 1408419.85 | 1853426.56 |
| 1327499.22 | 1443666.95 | 1197489.12 | 1288186.93 | 1239671.04 | 1225378.89 |
| 1272627.13 | 1325114.37 | 1188462.1 | 1272359.18 | 1249007.79 | 1186699.11 |
| 1016491.37 | 814964.69 | 1049857.99 | 1159042.36 | 704522.27 | 1133728.32 |
| 842810.94 | 919733.13 | 757732.39 | 849711.74 | 798842.63 | 767048.73 |
| 678240.05 | 643884.53 | 641593.73 | 721511.57 | 539269.17 | 696680.98 |
| 625656.88 | 639909.89 | 605797.07 | 632333.79 | 644545.18 | 580274.83 |
| 585371.9 | 593007.38 | 556121.15 | 591857.33 | 553362.73 | 560884.56 |
| 540039.99 | 577759.97 | 555230.95 | 551098.13 | 567769.03 | 520825.32 |
| 544738.52 | 589209.05 | 567036.28 | 532177.23 | 548540.11 | 518480.79 |
| 530738.99 | 558486.8 | 493654.84 | 531116.15 | 474228.14 | 515476.71 |
| 509757 | 553473.2 | 456276.05 | 507765.29 | 453896.33 | 472425.23 |
| 474193.74 | 466540.37 | 382954.43 | 473187.77 | 390127.37 | 505068.78 |
| 497846.31 | 517767.86 | 472662.27 | 502093.18 | 442257.87 | 465877.05 |
| 495376.59 | 530379.48 | 460172.86 | 501807.13 | 466427.63 | 455617.24 |
| 440860.32 | 496125.97 | 409543.06 | 440938.27 | 408922.46 | 417326.03 |
| 417261.59 | 402453.28 | 547157.64 | 441525.09 | 386048.76 | 451558.24 |
| 427155.82 | 441950.25 | 412082.98 | 430740.6 | 430016.88 | 400550.76 |
| 435831.22 | 471150.08 | 386854.97 | 407821.84 | 437140.55 | 387521.18 |
| 421375.41 | 443860.91 | 394369.49 | 429099.69 | 394426.49 | 391483.49 |
| 485710.28 | 456612.65 | 486362.31 | 474449.89 | 377267.21 | 510006.02 |
| 401238.69 | 437771.95 | 378181.17 | 388987.42 | 371786.74 | 382194.72 |
| 372573.22 | 408147.44 | 357627.88 | 355048.3 | 315526.67 | 376641.81 |
| 352035.98 | 348669.52 | 347502.37 | 354121.44 | 316061.66 | 351615.23 |
| 349069.49 | 385844.66 | 324566.47 | 348224.66 | 323745.85 | 320695.08 |
| 335464.93 | 370208.1 | 327281.84 | 331817.5 | 319839.56 | 334310.5 |
| 317070.87 | 383467.89 | 268668.52 | 279088.59 | 324904.58 | 258238.01 |
| 308392.69 | 307080.71 | 311549.96 | 335861.55 | 248415.47 | 298912.26 |
| 308697 | 330114.93 | 296104.75 | 307848.65 | 278634.56 | 298631.07 |
| 300560.97 | 370731.02 | 259236.68 | 276811.35 | 273708.11 | 278491.07 |
| 295673.74 | 323629.77 | 330397.16 | 300039.37 | 319791.85 | 293379.9 |
| 304665.91 | 326603.89 | 310451 | 296962.35 | 188559.9 | 330495.91 |
| 286045.82 | 287796.59 | 279718.29 | 294471.8 | 269457.64 | 270610.58 |
| 275239.86 | 290517.78 | 231778.37 | 259678.1 | 280159.12 | 234235.14 |
| 287835.7 | 248329.4 | 299318.95 | 330024.25 | 283736.55 | 270719.82 |
| 278323.77 | 297217.1 | 250655.61 | 281599.92 | 271088.43 | 261152.49 |
| 273753.96 | 290107.12 | 294755.04 | 273685.95 | 248206.04 | 276174.7 |
| 278848.58 | 307090.23 | 263136.12 | 279534.43 | 279238.49 | 252958.04 |
| 266477.52 | 266100.3 | 250977.73 | 278240.09 | 227626.55 | 250265.02 |
| 271267.07 | 294425.6 | 244751.93 | 256115.13 | 235419.65 | 261262.28 |
| 255004.83 | 257999.88 | 294589.99 | 259056.81 | 286320.93 | 269905.67 |
| 291242.94 | 317377.09 | 241756.91 | 282171.07 | 271200.98 | 277844.17 |
| 286344.17 | 319214.64 | 236248.35 | 262484.5 | 214354.62 | 245694.54 |
| 251630.39 | 240841.06 | 249414.48 | 266264.38 | 219985.77 | 239662.17 |
| 264141.85 | 281371.29 | 236037.81 | 262424.15 | 252314.63 | 245241.76 |
| 251904.04 | 264262.1 | 253843.13 | 257390.01 | 243745.29 | 243611 |
| 244675.59 | 244205.63 | 197264.09 | 255729.07 | 221148.85 | 222179.07 |
| 242765.23 | 276902.63 | 217946.6 | 227389.75 | 255553.01 | 204651.31 |
| 219227.63 | 213905.9 | 224759.3 | 226224.18 | 196098.88 | 198886.3 |
| 226282.62 | 251615.08 | 178638.76 | 222532.6 | 233411.17 | 207878.97 |
| 214911.68 | 245391.82 | 205008.02 | 207242.55 | 210646.08 | 193597.93 |
| 204822.49 | 223356.81 | 191755.63 | 195239.68 | 202692.45 | 188997.18 |
| 185947.87 | 129842.13 | 320601.76 | 249067.18 | 129319.88 | 217014.88 |
| 182006.21 | 172313.75 | 164420.67 | 174764.36 | 172924.45 | 172788.43 |

| 187848.43 | 187140.3 | 172653.69 | 189287.41 | 107110.13 | 207097.85 |
| --- | --- | --- | --- | --- | --- |
| 178294.65 | 189275.06 | 182611.55 | 187311.65 | 151977.96 | 180794.94 |
| 174215.17 | 194916.77 | 161533.32 | 170728.2 | 184808.86 | 156794.01 |
| 174861.09 | 177770.12 | 141273.67 | 187805.22 | 151356.37 | 149787.47 |
| 169217.65 | 188828.39 | 173424.56 | 153187.89 | 122116.15 | 175967.72 |
| 169406.74 | 158747.55 | 168684.74 | 188354.85 | 139414.83 | 169306.41 |
| 167143.09 | 176536.96 | 176211.42 | 184013.57 | 109684.42 | 145648.1 |
| 156643.23 | 158575.53 | 149704.01 | 149016.71 | 135130.06 | 140046.56 |
| 140755.01 | 141781.72 | 133209.9 | 139824.24 | 149340.71 | 129402.23 |
| 134270.87 | 146804.83 | 127326.48 | 137398.93 | 136023.46 | 121939.58 |
| 130176.74 | 153500.93 | 82160.46 | 127560.71 | 114359.55 | 94483.06 |
| 145446.5 | 202502.29 | 164995.96 | 125594.48 | 98121.31 | 152436.45 |
| 123127.99 | 118494.67 | 119716.4 | 120630.75 | 104172.94 | 130463.69 |
| 123922.98 | 136991.12 | 126148.86 | 123213.21 | 118155.57 | 126159.82 |
| 115084.53 | 112003.24 | 112041.94 | 122939.8 | 98445.92 | 108017.02 |
| 123257.83 | 116349.22 | 127125.78 | 124805.8 | 130527.35 | 113718.66 |
| 112209.75 | 118137.18 | 110342.01 | 123054.94 | 102719.22 | 108987.64 |
| 114920.04 | 115171.18 | 104890.39 | 113391.29 | 70943.32 | 123374.63 |
| 108488.76 | 146231.9 | 87161.9 | 93184.59 | 100582.19 | 83936.96 |
| 111777.01 | 123510.9 | 118800.14 | 114377.49 | 105062.75 | 101466.19 |
| 113747.92 | 125235.58 | 108851 | 111843.47 | 115824.36 | 101167.41 |
| 108968.12 | 102686.27 | 105685.32 | 122792.33 | 92484.48 | 114005.92 |
| 106640.05 | 115588.43 | 115086.43 | 109971.36 | 104607.34 | 112819.69 |
| 104879.11 | 124756.81 | 90436.04 | 103464.01 | 70730.72 | 110028.75 |
| 119028.42 | 178348.35 | 81650.2 | 96667.68 | 93752.05 | 91330.57 |
| 97035.21 | 107356.21 | 65819.26 | 77907.7 | 186921.04 | 71204.88 |
| 100611.65 | 117611.47 | 84942.33 | 96354.15 | 94614.88 | 84433.99 |
| 89875.34 | 96626.8 | 87947.53 | 91685.48 | 90335.38 | 92547.51 |
| 93273.66 | 110971.8 | 72214.56 | 89120.8 | 100654.53 | 77559.26 |
| 91677.34 | 90557.44 | 92825.75 | 94655.55 | 81858.31 | 90570.02 |
| 86616.72 | 110244.78 | 72341.21 | 76182.78 | 89076.92 | 68200.94 |
| 81299.22 | 93370.92 | 77164 | 75391.67 | 81437.74 | 80250.6 |
| 81869.23 | 80463.83 | 73807.03 | 83862.6 | 76794.16 | 78576.52 |
| 97993.46 | 138863.39 | 56816.45 | 87406.72 | 64104.13 | 72151.87 |
| 76700.55 | 83781.01 | 68669.13 | 77337.51 | 74673.81 | 76746.86 |
| 84897.25 | 118875.52 | 64396.02 | 70773.12 | 68808.18 | 66581.52 |
| 76742.97 | 83990.66 | 77302.32 | 81423.5 | 79255.17 | 74074.93 |
| 75151.34 | 90181.26 | 60849.66 | 64425.48 | 78811.3 | 58344.52 |
| 75873.08 | 106701.71 | 65230.05 | 71117.69 | 51990.82 | 61009.44 |
| 71007.7 | 73211.88 | 78884.57 | 68101.19 | 59111.82 | 94216.28 |
| 76980.82 | 86354.46 | 76750.33 | 77560.12 | 70830.46 | 83314.9 |
| 75720.11 | 106133.43 | 57198.95 | 67064.44 | 53478.94 | 69385.41 |
| 68729.07 | 78748.47 | 60432.68 | 65725.21 | 65752.37 | 66975.72 |
| 70948.47 | 67206.4 | 60460.79 | 74029.6 | 57060.31 | 60949.17 |
| 61533.47 | 61842.62 | 60824.93 | 64367.03 | 60732.27 | 59048.39 |
| 62425.9 | 70207.58 | 44563.76 | 41270.39 | 127627.12 | 39611.97 |
| 69825.02 | 123865.03 | 32892.81 | 55244.37 | 44584.75 | 42851.35 |
| 64403.12 | 58413.7 | 60999.67 | 62820.49 | 58560.7 | 60557.57 |
| 62393 | 72416.22 | 59387.76 | 59279.46 | 51388.21 | 59932.12 |
| 63160.62 | 74020.45 | 64745.7 | 61977.69 | 51093.91 | 65205.36 |
| 61395.85 | 73257.6 | 55187.9 | 60037.66 | 60060.69 | 56874.36 |
| 64468.43 | 67068.1 | 54094.82 | 64128.47 | 63205.9 | 68125.33 |
| 55917.31 | 61668.94 | 37023.74 | 44641.56 | 99365.07 | 47125.11 |
| 56258.14 | 69905.14 | 45737.22 | 47711.42 | 68573.43 | 38237.61 |
| 53874.09 | 64896.11 | 55814.07 | 50797.69 | 45375.9 | 52896.81 |
| 52753.3 | 65661.25 | 39257.31 | 43902.42 | 51642.78 | 37399.22 |
| 48814.5 | 55052.35 | 37418.54 | 41176.7 | 43968.71 | 39634.69 |
| 50837.78 | 58737.63 | 52551.08 | 47628.9 | 46386.75 | 48409.32 |

| 43677.49 | 48328.16 | 35094.38 | 43512.37 | 51460.03 | 35462.52 |
| --- | --- | --- | --- | --- | --- |
| 48596.15 | 61153.25 | 39608.64 | 37485.59 | 53391.45 | 35061.39 |
| 40738.83 | 53354.37 | 32073.72 | 37594.63 | 26109.88 | 47419.19 |
| 46385.71 | 59224.64 | 35859.58 | 43804.07 | 45603.76 | 35664.03 |
| 48973.87 | 52191.97 | 60379.91 | 50449.14 | 46076.1 | 60976.42 |
| 47521.87 | 37642.82 | 69408.95 | 57704.31 | 40258.65 | 53005.22 |
| 52468.02 | 74643.42 | 27841.38 | 42174.92 | 48584.76 | 38654.32 |
| 41217.87 | 34246.13 | 48875.82 | 40453.16 | 25679.49 | 112918.73 |
| 42148.1 | 45051.69 | 34324.79 | 45472.62 | 28703.3 | 35099.46 |
| 41413.7 | 32579.73 | 46818.67 | 45831.22 | 41090.51 | 40966.43 |
| 36068.68 | 35829.74 | 30942.21 | 40837.37 | 28024.11 | 33475.55 |
| 41689.19 | 33454.99 | 54122.76 | 46029.3 | 36388.88 | 50489.08 |
| 39307.94 | 40394.83 | 37749.54 | 39813.13 | 30691.37 | 40395.29 |
| 45713.06 | 61488.38 | 28344.84 | 40147.29 | 21946.34 | 44223.18 |
| 37666.78 | 44430 | 37819.45 | 38379.46 | 38717.96 | 34719.56 |
| 37915.75 | 41679.37 | 25638.37 | 38203.37 | 22818.75 | 31617.02 |
| 37490.55 | 41104.89 | 31037.5 | 36253.4 | 38214.26 | 35930.88 |
| 35020.94 | 40473.66 | 25084.42 | 34621.98 | 25000.36 | 29865.42 |
| 32612.94 | 30659.09 | 30043.43 | 33329.49 | 12983.22 | 27226.86 |
| 35268.89 | 40138.23 | 30788.84 | 32575.65 | 36825.26 | 33882.02 |
| 34043.99 | 41170.27 | 24948.66 | 26051.91 | 65070.86 | 21543.1 |
| 31566.79 | 35688.35 | 37727.39 | 32122.21 | 26654.61 | 32047.04 |
| 33460.54 | 38830.37 | 32192.64 | 30772.71 | 33117.71 | 28781.24 |
| 28738.53 | 25692.4 | 41442 | 21256.5 | 15278.4 | 118099.38 |
| 28701.66 | 34540.61 | 20410.41 | 20269.32 | 65944.63 | 19090.77 |
| 30921.3 | 34092.23 | 28795.93 | 30016.06 | 37599.59 | 26366 |
| 28974.38 | 33676.92 | 26597.01 | 28830.23 | 25676.28 | 33269.45 |
| 28559.55 | 37749.9 | 19089.26 | 26323.15 | 30389.08 | 17450.04 |
| 29616.3 | 35094.33 | 24382.26 | 28059.73 | 25404.02 | 24808.28 |
| 29602.56 | 32134.19 | 29290.76 | 30826.24 | 37329.03 | 30570.24 |
| 30805.23 | 26175.04 | 27287.56 | 34298.58 | 29641.23 | 29410.63 |
| 39716.49 | 21819.16 | 25293 | 57884.5 | 27073.8 | 42600.97 |
| 27458.26 | 28543.16 | 29923.54 | 25832.78 | 13041.88 | 31162.76 |
| 28725.95 | 34403.66 | 23974.42 | 27399.98 | 24853.36 | 25719.08 |
| 28315.28 | 33967.76 | 23836.89 | 25512.71 | 22883.71 | 27336.89 |
| 24778.53 | 33745.92 | 19404.36 | 22516.14 | 38039.09 | 18938.88 |
| 26183.01 | 38739.54 | 14568.09 | 24945.82 | 21545.28 | 24253.17 |
| 27413.29 | 31771.99 | 23098.42 | 27812.23 | 25014.36 | 24687.83 |
| 21500.76 | 25209.84 | 19430.55 | 20382.2 | 35161.29 | 17263.76 |
| 24521 | 20472.46 | 19484.6 | 25754.81 | 13062.37 | 23321.41 |
| 25049.47 | 34475.92 | 16480.35 | 21780.4 | 23987.61 | 15701.25 |
| 22833.61 | 20787.53 | 19474.89 | 28783.12 | 26082.63 | 23077.54 |
| 21778.88 | 25807.08 | 19090.61 | 21015.64 | 33705.84 | 17718.83 |
| 23359.88 | 24648.91 | 23134.1 | 24255.98 | 24172.1 | 23875.09 |
| 23941.8 | 24903.83 | 22747.43 | 24070.81 | 23416.69 | 22551 |
| 23256.88 | 24451.16 | 22807.6 | 24104.98 | 23794.35 | 22822.64 |
| 23254.88 | 24446.16 | 22806.6 | 24103.98 | 23794.35 | 22820.64 |
| 25213.3 | 38807.86 | 4930.5 | 24520.36 | 7824.1 | 17041.31 |
| 22973.38 | 24965.64 | 23760.77 | 21430.56 | 24713.41 | 22826.5 |
| 21867.97 | 27034.92 | 15005.02 | 20944.23 | 22077.94 | 13857.08 |
| 19402.5 | 23519.04 | 13671 | 15086.16 | 48723.8 | 10258.3 |
| 19402.5 | 23519.04 | 13671 | 15086.16 | 48723.8 | 10257.97 |
| 21222.39 | 23518.5 | 19051.6 | 23390.98 | 20795.27 | 19196.43 |
| 16563.83 | 19961.03 | 21574.05 | 17673.28 | 6307.63 | 19887.71 |
| 16560.96 | 21222.01 | 21195.6 | 18678.39 | 14622.44 | 16367.07 |
| 19233.62 | 21524.84 | 20770.51 | 20572.65 | 20303.44 | 19089.47 |
| 16533.29 | 12818.17 | 21837.35 | 16274.64 | 17255.85 | 22339.99 |
| 16344.97 | 17140.83 | 13141.73 | 13675.83 | 15461.5 | 18270.75 |

| 18374.56 | 26509.96 | 16391.74 | 18276.83 | 26162.39 | 18295.06 |
| --- | --- | --- | --- | --- | --- |
| 17261.47 | 17798.08 | 14792.52 | 17361.23 | 17675.94 | 13116.36 |
| 15476.49 | 11428.16 | 12345.57 | 14242.25 | 10404.59 | 14843.19 |
| 17585.66 | 21018.89 | 12002.99 | 16217 | 21080.58 | 14701.1 |
| 16152.82 | 21353.97 | 11642 | 12625 | 34807.8 | 9446 |
| 14865.35 | 24385.41 | 16582.9 | 11016.35 | 14732 | 9035.94 |
| 12989.78 | 12147.89 | 19028.98 | 14482.95 | 5053.48 | 12892.58 |
| 13347 | 6726.37 | 6497.92 | 13180.7 | 10144.95 | 10766.15 |
| 14252 | 19126.8 | 12115.5 | 13241.5 | 28486.63 | 7966.75 |
| 14562.58 | 14621.06 | 2867.8 | 18213.63 | 6514.15 | 11317.59 |
| 15387.58 | 24663.9 | 9508.83 | 13015.17 | 18469.07 | 13827.07 |
| 14811.84 | 24028.38 | 13416.69 | 12789.56 | 6805.62 | 15187.71 |
| 11358.36 | 12765.98 | 8744.95 | 11379.14 | 2542.45 | 5913.67 |
| 11288.03 | 12684.98 | 8743.95 | 11375.14 | 2541.45 | 5903.67 |
| 10607.64 | 10984.25 | 18017.78 | 10981.06 | 14259.93 | 11181.69 |
| 10502.64 | 10781.5 | 17690.28 | 10829.06 | 13882.18 | 10127.24 |
| 10024.16 | 17356.23 | 6323.99 | 7755.17 | 8506.07 | 3797.2 |
| 16123.48 | 52528.51 | 7611.51 | 5853.9 | 5364.29 | 8014.97 |
| 9756.89 | 12107.95 | 13236.7 | 9796.39 | 3267.7 | 6731.54 |
| 11416.08 | 11953.5 | 5810.07 | 10486.84 | 5454.51 | 9436.54 |
| 12204.08 | 17216.9 | 8032.5 | 12178 | 16559.4 | 11980.9 |
| 12204.08 | 17216.9 | 8032.5 | 12178 | 16559.4 | 11980.9 |
| 12204.08 | 17216.9 | 8032.5 | 12178 | 16559.4 | 11980.9 |
| 12204.08 | 17216.9 | 8032.5 | 12178 | 16559.4 | 11980.9 |
| 12204.08 | 17216.9 | 8032.5 | 12178 | 16559.4 | 11980.9 |
| 12204.08 | 17216.9 | 8032.5 | 12178 | 16559.4 | 11980.9 |
| 9549.91 | 12298.91 | 5604.15 | 8536 | 4512.76 | 9354.52 |
| 9549.91 | 12298.91 | 5604.15 | 8536 | 4512.76 | 9354.52 |
| 7243.72 | 6305.77 | 13475.28 | 7263.31 | 9903.78 | 9534.12 |
| 9448.39 | 4574.39 | 5559.22 | 10879.79 | 5510.57 | 8244.57 |
| 6435 | 9217.57 | 6138.75 | 5817.75 | 8220.15 | 3471.5 |
| 6310.31 | 6167 | 1321 | 7373 | 170 | 5155 |
| 6310.31 | 6167 | 1321 | 7373 | 170 | 5155 |
| 7325.97 | 5025.1 | 3809.78 | 11336.39 | 4838.78 | 9030.14 |
| 6243.99 | 6021 | 1110 | 7218 | 147 | 4146 |
| 6653.7 | 3369.77 | 4305.28 | 6495.06 | 3253.95 | 6143.85 |
| 6690.58 | 5235.67 | 12462.08 | 8265 | 3324.58 | 7158.83 |
| 6460.9 | 4201.15 | 6874.49 | 7572.41 | 5273.42 | 5919.98 |
| 4615.97 | 3591.87 | 365 | 4771 | 141 | 739.52 |
| 4842.16 | 10083.18 | 601.33 | 4330.92 | 351 | 1778.08 |
| 4362.7 | 9078.73 | 721.44 | 4076.82 | 450.12 | 1910.25 |
| 13093.67 | 60453.83 | 1526.66 | 1090.84 | 2127.34 | 1864.34 |
| 2992.33 | 3035.17 | 414 | 5614 | 506 | 1178.12 |
| 5890.83 | 4004.92 | 9714.83 | 6954 | 2265.33 | 6542.58 |
| 5890.83 | 4004.92 | 9714.83 | 6954 | 2265.33 | 6539.58 |
| 3757.24 | 2056.02 | 1761.1 | 5681.81 | 2544.69 | 2486.63 |
| 4606.5 | 9236.84 | 212.5 | 3583 | 4402 | 741.72 |
| 3509 | 4897.17 | 3161 | 1402.75 | 7299 | 1915.33 |
| 3457.17 | 7920.33 | 1420.58 | 1317.42 | 1975.42 | 1985.5 |
| 2701.16 | 2786.34 | 395.25 | 3281 | 151.75 | 1648.16 |
| 3412.66 | 2315.58 | 8488.24 | 4234.5 | 1989.83 | 6190.13 |
| 3137.42 | 2202.83 | 1814.58 | 2006.42 | 2411.25 | 2057.34 |
| 3382.83 | 8280.34 | 380.25 | 3210.5 | 130.75 | 1618.22 |
| 3409.66 | 1654 | 3927.33 | 3045 | 1964.67 | 5533.85 |
| 3201.67 | 7685 | 1377.58 | 1539.67 | 1980.75 | 1919.14 |
| 3188.67 | 7656.83 | 1375.58 | 1539.67 | 1980.75 | 1917.14 |
| 2370.49 | 2466.51 | 441.25 | 3213 | 144.75 | 1626.72 |
| 5316.73 | 19207.68 | 1146.33 | 1568.25 | 224.66 | 3128.52 |

| 2811.83 | 2235.5 | 153 | 2988 | 104 | 953.25 |
| --- | --- | --- | --- | --- | --- |
| 3252.17 | 7535.33 | 1450.33 | 821.92 | 1905 | 1741.92 |
| 1993 | 1902 | 143 | 2298 | 28 | 392 |
| 3532.46 | 13307.71 | 117 | 370.5 | 466 | 524.88 |
| 1961.66 | 1601.83 | 1346 | 774 | 1694 | 1724.92 |
| 1255.73 | 1023.84 | 2973.25 | 737.5 | 614.5 | 10537.87 |
| 845.13 | 1664.39 | 2231.4 | 1246.79 | 461.07 | 1476.8 |
| 2255.91 | 6571.5 | 1130.33 | 1459.25 | 195.66 | 3077.08 |
| 3556.8 | 13206.04 | 26.5 | 182 | 61 | 96.44 |
| 2138.75 | 1151.5 | 62.25 | 4261.25 | 1397.5 | 1890.65 |
| 2138.75 | 1151.5 | 62.25 | 4261.25 | 1397.5 | 1890.65 |
| 553.33 | 895.17 | 269.5 | 961 | 39 | 1083.05 |
| 814.75 | 542.5 | 479 | 999.25 | 175.83 | 308 |
| 1456.28 | 6266.87 | 22.25 | 61 | 41.75 | 36.69 |
| 224.74 | 224.86 | 421.2 | 316.06 | 26.86 | 923.46 |
| 64 | 383 | 1605 | 123 | 956 | 1283 |
| 898.33 | 456 | 126.67 | 783 | 1182 | 309.06 |
| 179.66 | 206.34 | 5 | 85 | 42 | 28 |
| 423.66 | 410.17 | 10 | 100.5 | 33 | 37.44 |
| 463.64 | 496.19 | 17 | 61 | 32 | 51.69 |
| 642.49 | 673.01 | 9 | 89.5 | 28 | 53.42 |
| 1166.5 | 6007.5 | 27 | 106.5 | 8 | 65.5 |
| 278.66 | 233 | 8 | 38 | 20 | 95.4 |
| 237.67 | 83 | 96.33 | 42.67 | 206.67 | 13.67 |
| 70.32 | 153 | 211 | 155 | 23 | 1009 |
| 66.32 | 146 | 211 | 155 | 23 | 1008 |
| 66.32 | 146 | 211 | 155 | 23 | 1008 |
| 66.32 | 146 | 211 | 155 | 23 | 1008 |
| 66.32 | 146 | 211 | 155 | 23 | 1008 |
| 66.32 | 146 | 211 | 155 | 23 | 1008 |
| 66.32 | 146 | 211 | 155 | 23 | 1008 |
| 66.32 | 146 | 211 | 155 | 23 | 1008 |
| 66.32 | 146 | 211 | 155 | 23 | 1008 |
| 66.32 | 146 | 211 | 155 | 23 | 1008 |
| 66.32 | 146 | 211 | 155 | 23 | 1008 |
| 66.32 | 146 | 211 | 155 | 23 | 1008 |
| 66.32 | 146 | 211 | 155 | 23 | 1008 |
| 66.32 | 146 | 211 | 155 | 23 | 1008 |
| 66.32 | 146 | 211 | 155 | 23 | 1008 |
| 1568 | 1485.17 | 0.012 | 112 | 0.012 | 22 |
| 308 | 69 | 4 | 293 | 27 | 243 |
| 308 | 69 | 4 | 293 | 27 | 243 |
| 127 | 108 | 173 | 54 | 25 | 261 |
| 18.33 | 25 | 17 | 25 | 6 | 57 |
| 18 | 7 | 20 | 25 | 25 | 60 |
| 18 | 7 | 20 | 25 | 25 | 60 |
| 2 | 5.17 | 1 | 1 | 0.012 | 2 |
| 2 | 5 | 1 | 1 | 0.012 | 2 |
| 2 | 5 | 1 | 1 | 0.012 | 2 |
| 2 | 5.17 | 1 | 1 | 0.012 | 2 |
| 2 | 5 | 1 | 1 | 0.012 | 2 |
| 2 | 5 | 1 | 1 | 0.012 | 2 |
| 2 | 5 | 1 | 1 | 0.012 | 2 |
| 15 | 1 | 14 | 3.25 | 3.33 | 44.83 |
| 70.33 | 81 | 1 | 4 | 1 | 14 |
| 70.33 | 81 | 1 | 4 | 1 | 13 |
| 70.33 | 81 | 1 | 4 | 1 | 13 |
| 70.33 | 81 | 1 | 4 | 1 | 13 |

| 70.33 | 81 | 1 | 4 | 1 | 13 |
| --- | --- | --- | --- | --- | --- |
| 63 | 53 | 6 | 4 | 0.012 | 4 |
| 79.32 | 162 | 0.012 | 0.012 | 0.012 | 6 |
| 76.99 | 69.34 | 2 | 4 | 0.012 | 2 |
| 0.012 | 0.012 | 0.012 | 1 | 5 | 2 |
| 56 | 53 | 0.012 | 4 | 0.012 | 6 |
| 56 | 53 | 0.012 | 4 | 0.012 | 2.5 |
| 56 | 53 | 0.012 | 4 | 0.012 | 1 |
| 4 | 5 | 2 | 1 | 1 | 0.5 |
| 8 | 0.012 | 2 | 0.012 | 0.012 | 0.012 |
| 0.012 | 0.012 | 0.012 | 0.012 | 0.012 | 7.23 |
| 0.012 | 0.012 | 0.012 | 0.012 | 0.012 | 36 |
| 0.012 | 0.012 | 0.012 | 0.012 | 0.012 | 0.012 |
| 0.012 | 0.012 | 0.012 | 0.012 | 0.012 | 0.012 |
| 0.012 | 0.012 | 0.012 | 0.012 | 0.012 | 2 |
| 0.012 | 0.012 | 0.012 | 0.012 | 0.012 | 3 |
| 0.012 | 0.012 | 0.012 | 0.012 | 0.012 | 0.012 |
| 0.012 | 0.012 | 0.012 | 0.012 | 0.012 | 3 |
| 0.012 | 0.012 | 0.012 | 0.012 | 0.012 | 0.012 |
| 0.012 | 0.012 | 0.012 | 0.012 | 0.012 | 0.012 |
| 0.012 | 0.012 | 0.012 | 0.012 | 0.012 | 0.2 |
| 0.012 | 0.012 | 0.012 | 0.012 | 0.012 | 1 |
| 0.012 | 0.012 | 0.012 | 0.012 | 0.012 | 1 |
| 0.012 | 0.012 | 0.012 | 0.012 | 0.012 | 0.012 |
| 0.012 | 0.012 | 0.012 | 0.012 | 0.012 | 0.012 |
| 0.012 | 0.012 | 0.012 | 0.012 | 0.012 | 1 |
| 0.012 | 0.012 | 0.012 | 0.012 | 0.012 | 1 |
| 0.012 | 0.012 | 0.012 | 0.012 | 0.012 | 1 |
| 0.012 | 0.012 | 0.012 | 0.012 | 0.012 | 1 |
| 0.012 | 0.012 | 0.012 | 0.012 | 0.012 | 1 |
| 0.012 | 1 | 0.012 | 0.012 | 0.012 | 0.012 |
| 0.012 | 0.012 | 0.012 | 0.012 | 0.012 | 1 |
| 0.012 | 0.012 | 1 | 0.012 | 0.012 | 0.012 |
| 0.012 | 0.012 | 0.012 | 0.012 | 0.012 | 1 |
| 0.012 | 0.012 | 0.012 | 0.012 | 0.012 | 0.5 |
| 0.012 | 0.012 | 0.012 | 0.012 | 0.012 | 2 |
| 0.012 | 0.012 | 0.012 | 0.012 | 0.012 | 0.012 |
| 0.012 | 0.012 | 0.012 | 0.012 | 0.012 | 0.012 |
| 0.012 | 0.012 | 0.012 | 0.012 | 0.012 | 1 |
| 0.012 | 0.012 | 0.012 | 0.012 | 0.012 | 1 |
| 0.012 | 0.012 | 0.012 | 0.012 | 0.012 | 1 |
| 0.012 | 0.012 | 0.012 | 0.012 | 0.012 | 0.012 |
| 0.012 | 0.012 | 0.012 | 0.012 | 0.012 | 0.012 |
| 0.012 | 0.012 | 0.012 | 0.012 | 0.012 | 0.012 |
| 0.012 | 0.012 | 0.012 | 0.012 | 0.012 | 0.012 |
| 0.012 | 0.012 | 0.012 | 0.012 | 0.012 | 0.012 |
| 0.012 | 0.012 | 0.012 | 0.012 | 0.012 | 0.012 |
| 0.012 | 0.012 | 0.012 | 0.012 | 0.012 | 0.012 |
| 0.012 | 0.012 | 0.012 | 0.012 | 0.012 | 0.012 |
| 0.012 | 0.012 | 0.012 | 0.012 | 0.012 | 0.012 |
| 0.012 | 0.012 | 0.012 | 0.012 | 0.012 | 0.012 |
| 0.012 | 0.012 | 0.012 | 0.012 | 0.012 | 0.012 |
| 0.012 | 0.012 | 0.012 | 0.012 | 0.012 | 0.012 |
| 0.012 | 0.012 | 0.012 | 0.012 | 0.012 | 0.012 |
| 0.012 | 0.012 | 0.012 | 0.012 | 0.012 | 0.012 |
| 0.012 | 0.012 | 0.012 | 0.012 | 0.012 | 0.012 |
| 0.012 | 0.012 | 0.012 | 0.012 | 0.012 | 0.012 |
| 0.012 | 0.012 | 0.012 | 0.012 | 0.012 | 0.012 |

| 0.012 | 0.012 | 0.012 | 0.012 | 0.012 | 0.012 |
| --- | --- | --- | --- | --- | --- |
| 0.012 | 0.012 | 0.012 | 0.012 | 0.012 | 0.012 |
| 0.012 | 0.012 | 0.012 | 0.012 | 0.012 | 0.012 |
| 0.012 | 0.012 | 0.012 | 0.012 | 0.012 | 0.012 |
| 0.012 | 0.012 | 0.012 | 0.012 | 0.012 | 0.012 |
| 0.012 | 0.012 | 0.012 | 0.012 | 0.012 | 0.012 |
| 0.012 | 0.012 | 0.012 | 0.012 | 0.012 | 0.012 |
| 0.012 | 0.012 | 0.012 | 0.012 | 0.012 | 0.012 |
| 0.012 | 0.012 | 0.012 | 0.012 | 0.012 | 0.012 |
| 0.012 | 0.012 | 0.012 | 0.012 | 0.012 | 0.5 |
| 0.012 | 0.012 | 0.012 | 0.012 | 0.012 | 0.5 |
| 0.012 | 0.012 | 0.012 | 0.012 | 0.012 | 0.2 |
| 0.012 | 0.012 | 0.012 | 0.012 | 0.012 | 0.012 |
| 0.012 | 0.012 | 0.012 | 0.012 | 0.012 | 0.012 |
| 0.012 | 0.012 | 0.012 | 0.012 | 0.012 | 0.012 |
| 0.012 | 0.012 | 0.012 | 0.012 | 0.012 | 0.012 |
| 0.012 | 0.012 | 0.012 | 0.012 | 0.012 | 0.012 |
| 0.012 | 0.012 | 0.012 | 0.012 | 0.012 | 0.012 |

| EAC_25 | EAC_26 | EAC_27 | EAC_28 | EAC_5 | EAC_6 |
| --- | --- | --- | --- | --- | --- |
| 8939371.95 | 8469687.76 | 9110812.2 | 8727809.14 | 9068161.1 | 8684605.36 |
| 4119172.56 | 4033706.84 | 4369399.25 | 3823650.22 | 4278200.52 | 4199766.4 |
| 2093989.61 | 2008069.28 | 2123730.51 | 1964230.46 | 2122244.44 | 2104879.34 |
| 1716836.65 | 1827343.1 | 1936168.15 | 1600753.25 | 1780760.6 | 1771189.63 |
| 1341971.45 | 1202232.5 | 1322036.27 | 1254997.92 | 1353117.38 | 1308724.55 |
| 1268057.64 | 1121912.07 | 1295126.54 | 1266795.31 | 1295042.95 | 1207403.9 |
| 1085954.75 | 1073233.52 | 1079390.93 | 1146673.05 | 1057471.4 | 1063649.08 |
| 817822.32 | 737022.62 | 823401.44 | 794304.86 | 862535.97 | 824965.34 |
| 664583.07 | 673781.76 | 689126.87 | 797966.38 | 656808.72 | 647909.03 |
| 617728.48 | 552902.67 | 654618.65 | 600190.21 | 639108.87 | 585511.36 |
| 582918.37 | 532332.4 | 597733.16 | 592317.7 | 588974.47 | 553126.75 |
| 532416.51 | 488184.49 | 601005.67 | 550331.83 | 548929.95 | 528255.74 |
| 525641.13 | 491936.21 | 617934.3 | 520736.18 | 554450.19 | 535980.09 |
| 519638.38 | 513576.33 | 533698.56 | 494438.35 | 539690.54 | 507694.12 |
| 482149.42 | 465169.2 | 518581.9 | 444358.11 | 531032.41 | 526224.29 |
| 496153.29 | 491672.27 | 422263.48 | 455709.62 | 487522.07 | 484675.71 |
| 515292.48 | 471571.66 | 507399.86 | 470631.98 | 514610.74 | 497612.07 |
| 487959.2 | 445654.96 | 503841 | 493738.19 | 494891.45 | 467707.64 |
| 427028.93 | 401022.45 | 465544.39 | 431857.13 | 441542.55 | 426950.12 |
| 399224.48 | 406900.14 | 583937.04 | 411782.37 | 438440.09 | 445260.17 |
| 426027.71 | 385716.22 | 441082.31 | 413863.93 | 435171.4 | 405057.77 |
| 442239.31 | 392229.83 | 418189.54 | 382845.96 | 439245.84 | 393574.75 |
| 419016.52 | 376359.61 | 437268.86 | 433983.54 | 425424.7 | 394364.08 |
| 509099.93 | 523539.53 | 473683.55 | 546420.35 | 460723.13 | 412362.29 |
| 393486.84 | 367159.54 | 423969.72 | 386450.99 | 406256.8 | 379182.55 |
| 349843.22 | 374352.66 | 381473.69 | 319211.69 | 374133.67 | 374148.73 |
| 355225.72 | 325634.86 | 361539.56 | 337796.13 | 367882 | 353387.71 |
| 344272.37 | 312531.29 | 352960.5 | 339697.11 | 348248.5 | 328477.11 |
| 336572.95 | 309875.64 | 366240 | 315648.57 | 340443.22 | 333657.26 |
| 316818.01 | 281669.3 | 298572.59 | 222704.1 | 327273.5 | 321148.59 |
| 343040.36 | 325279.91 | 319109.9 | 302108.42 | 340441.56 | 349540.87 |
| 308265.61 | 292255.5 | 309064.35 | 300226.14 | 309917.87 | 300157.19 |
| 290664.36 | 292717.85 | 273331.12 | 225538.46 | 304353.64 | 294064.28 |
| 273652.39 | 263610.89 | 355144.78 | 285978.67 | 297418.75 | 274727 |
| 302539.49 | 344461.35 | 319629.41 | 281144.63 | 299975.65 | 314420.55 |
| 287659.42 | 258395.53 | 295605.86 | 285283.14 | 291997.12 | 272587.36 |
| 271559.34 | 262749.2 | 224039.96 | 231256.17 | 279733.6 | 274950.13 |
| 316668.66 | 309516.91 | 288668.5 | 293811.1 | 289412.1 | 261452 |
| 279159.24 | 250695.4 | 271322.67 | 277604.56 | 284207.66 | 268643.99 |
| 269535.54 | 268138.48 | 301867.47 | 238006.69 | 281695.99 | 270667.39 |
| 264635.69 | 244516.06 | 288237.84 | 271557.78 | 276533.31 | 253470.5 |
| 301834.83 | 267095.56 | 270848.42 | 294049.81 | 270890.56 | 266901.7 |
| 258615.37 | 258728.02 | 255736.57 | 251703.27 | 272987.69 | 269303.38 |
| 237440.41 | 231751.38 | 323455.88 | 262685.37 | 261180.07 | 255357.7 |
| 281487.63 | 266223.01 | 264795.16 | 333122.07 | 275126.2 | 241953.45 |
| 283031.37 | 261344.99 | 243994.36 | 249012.77 | 280514.15 | 271199.18 |
| 289457.26 | 254887.93 | 263266.54 | 259419.02 | 263199.28 | 261390.84 |
| 256298.23 | 231425.74 | 259121.04 | 284280.25 | 261043.22 | 236731.02 |
| 253493.44 | 235693.18 | 274013.83 | 261698.91 | 254435.74 | 237333.82 |
| 247523.55 | 210892.54 | 242332.7 | 283736.95 | 253478.63 | 265845.27 |
| 234263.14 | 200554.14 | 252328.7 | 233522.1 | 245651.35 | 233984.44 |
| 229059.53 | 214335.58 | 221600.31 | 161883.35 | 231356.5 | 212111.02 |
| 235787.05 | 177776.99 | 228964.75 | 259756 | 224820.09 | 197102.04 |
| 208675.8 | 189473.3 | 228034.31 | 191031.09 | 217004.11 | 204662.35 |
| 193305.18 | 177492.19 | 211043.69 | 198251.09 | 204461.46 | 193817.53 |
| 189276.93 | 208164.66 | 282727.78 | 186186.04 | 194686.06 | 206355.65 |
| 173267.45 | 184749.35 | 164418.14 | 137916.25 | 190855.6 | 184087.75 |

| 181067.07 | 207593.91 | 174136.13 | 192953.59 | 183154.96 | 205579.82 |
| --- | --- | --- | --- | --- | --- |
| 172174.51 | 170801.11 | 196130.72 | 168600.52 | 183587.13 | 173254.78 |
| 166893.9 | 149980.14 | 178852.06 | 156441.28 | 178338.97 | 161494.06 |
| 211569.61 | 169955.07 | 137565.14 | 197582.09 | 178181.67 | 176867.73 |
| 161175.87 | 185512.85 | 182875.14 | 128584.65 | 171394.74 | 170230.32 |
| 172702.62 | 164246.33 | 171018.78 | 166827.65 | 172203.21 | 163135.11 |
| 185185.05 | 188793.12 | 169259 | 194155.51 | 165231.22 | 178784.47 |
| 158624.63 | 160227.98 | 153469.57 | 124029.24 | 161922.27 | 151805.9 |
| 141670.95 | 120731.22 | 147125.3 | 146165.31 | 140469.39 | 128997.58 |
| 127926.96 | 114138.02 | 149515.27 | 146680.85 | 136788.41 | 137920.36 |
| 129056.66 | 111526.83 | 96181.08 | 125826.38 | 131829.68 | 140187.39 |
| 132748.24 | 171808.43 | 171518.25 | 111186.83 | 130004.86 | 127190.21 |
| 117607.13 | 117155.31 | 140529.01 | 113449.38 | 129962.88 | 128929.14 |
| 128202.37 | 121118.04 | 132003.66 | 127720.86 | 124311.41 | 119537.16 |
| 131036.28 | 118084.4 | 118340.89 | 94423.94 | 123222.53 | 117112.07 |
| 128306.97 | 113292.28 | 142079.08 | 117802.6 | 125733.7 | 109839.68 |
| 111201.76 | 98239.98 | 130903.61 | 125620.99 | 113862.66 | 118824 |
| 111709.64 | 122786.44 | 112013.95 | 112096.52 | 115786.9 | 128596.37 |
| 95512.38 | 99154.94 | 83831.09 | 53871.09 | 113240.27 | 113676.6 |
| 112226.54 | 107418.29 | 119090.65 | 91616.77 | 116320.25 | 114344.22 |
| 107521.63 | 100174.5 | 119780.83 | 102886.94 | 114736.22 | 108081.71 |
| 116623.52 | 120839.08 | 117798.11 | 137347.75 | 109144.34 | 104582.42 |
| 110684.48 | 103655.11 | 124236.68 | 109796.68 | 110347.31 | 113464.22 |
| 111532.29 | 96882.17 | 103318.4 | 114969.44 | 105260.73 | 116587.63 |
| 105536.68 | 116480.38 | 78227.66 | 108605.95 | 101109.25 | 98090.48 |
| 79430.72 | 53753.21 | 98717.83 | 74354.29 | 102133.13 | 88133.77 |
| 102853.12 | 93250.31 | 88805.08 | 90283.47 | 102464.62 | 103810.79 |
| 96375.13 | 86237.78 | 94524.48 | 98929.88 | 92384.33 | 94432.26 |
| 89513.45 | 73368.88 | 86588.18 | 97044.51 | 92489.7 | 87996.59 |
| 92620.43 | 87196.93 | 97623 | 91334.1 | 93924.2 | 89889.95 |
| 79892.06 | 73964.35 | 82121.63 | 71689.57 | 85107.78 | 93067.15 |
| 73460.52 | 71797.67 | 87771.9 | 75314.04 | 81279.76 | 78785.29 |
| 82122.58 | 75215.31 | 79914.3 | 93030 | 81783.15 | 77675.64 |
| 91557.98 | 77733.89 | 72226.54 | 129958.89 | 80439.94 | 80062.9 |
| 76246.52 | 67523.43 | 82280.21 | 84950.8 | 77873.33 | 76022.22 |
| 72739.93 | 71885.99 | 76253.54 | 88515.87 | 74581.45 | 76898.16 |
| 74147.07 | 67103.93 | 94853.97 | 95122.17 | 74983.01 | 76892.22 |
| 59579.11 | 56036.93 | 79286.6 | 69515.46 | 72108.02 | 74793.26 |
| 69789.8 | 78205.16 | 68112.55 | 59835.61 | 73001.77 | 73486.98 |
| 68835.8 | 70602.98 | 84099.51 | 94129.07 | 70811.77 | 67248.34 |
| 80991.15 | 73347.3 | 89465.28 | 79452.97 | 74737.69 | 67263.08 |
| 66581.79 | 80696.11 | 57748.71 | 67451.03 | 67158.04 | 60137.47 |
| 69502.59 | 63423.81 | 67834.7 | 59197.22 | 71091.21 | 69213.32 |
| 73847.03 | 64736.85 | 65281.12 | 81889.14 | 68992.9 | 67669.14 |
| 70079.43 | 58868.47 | 64748.61 | 61443.94 | 64308.85 | 57815.33 |
| 50313.53 | 33301.8 | 62363.67 | 45655.84 | 65805.73 | 57892.26 |
| 66031.4 | 59906.3 | 39126.38 | 51543.46 | 63625.39 | 72953.88 |
| 64013.51 | 58536.2 | 64188.74 | 64495.49 | 62645.51 | 57049.38 |
| 56630.96 | 65915.21 | 61371.03 | 49038.88 | 62088.95 | 58118.4 |
| 64366.03 | 66502.46 | 71630.21 | 60192.34 | 61897.83 | 59349.73 |
| 57343.84 | 50927.03 | 62153.31 | 59081.02 | 59583.45 | 52165.29 |
| 65581.85 | 55039 | 70697.18 | 72897.19 | 59615.01 | 50980 |
| 42052.63 | 32627.21 | 64978.16 | 50664.42 | 57382.96 | 47770.52 |
| 45089.97 | 38948.95 | 53536.67 | 52231.15 | 52675.25 | 52511.27 |
| 44522.33 | 46872.39 | 63650.05 | 49278.85 | 54713.16 | 58909.13 |
| 45420.05 | 43109.61 | 46115.25 | 43899.73 | 52280.01 | 58908.51 |
| 37659.19 | 39488.48 | 45534.7 | 33521.9 | 53729.72 | 61254.48 |
| 45214.83 | 53552.45 | 55468.35 | 42741.95 | 49543.42 | 43540.24 |

| 49864.61 | 37747.7 | 41133.45 | 37935.32 | 46755.55 | 44975.9 |
| --- | --- | --- | --- | --- | --- |
| 35716.88 | 36426.11 | 46912.25 | 44264.21 | 45975.51 | 51195.18 |
| 37926.17 | 42580.17 | 37857.62 | 24618.31 | 41665.55 | 51276.06 |
| 50073.44 | 39935.38 | 39022.93 | 40521.31 | 47826.56 | 52782.25 |
| 52959.87 | 51455.56 | 70079.85 | 53382.62 | 46205.77 | 40644 |
| 45794.16 | 50207.87 | 61791.96 | 38471.32 | 46560.73 | 38105.77 |
| 45892.63 | 39952.37 | 35869.25 | 58443.9 | 46343.32 | 40859.59 |
| 49968.27 | 61480.78 | 82028.92 | 65080.11 | 35483.06 | 39221.59 |
| 46481.75 | 40291.37 | 36799.45 | 41474.75 | 43087.96 | 47193.06 |
| 46386.96 | 43668.18 | 50066.1 | 44540.87 | 43532.69 | 36680.4 |
| 45772.85 | 35934.19 | 31139.95 | 40514.3 | 39132.38 | 40363.48 |
| 42378.13 | 43054.92 | 53814.01 | 43686.26 | 41176.19 | 38598.57 |
| 38476.97 | 40225.73 | 39283.62 | 39673.41 | 38544.54 | 40448.32 |
| 40004.75 | 54899.74 | 20096.28 | 46824.76 | 36667.35 | 25114.49 |
| 35712.17 | 32288.13 | 44944.86 | 43206.24 | 36938.38 | 38397.32 |
| 36739.77 | 39686.34 | 21511.61 | 25454.79 | 39963.48 | 43579.56 |
| 34489.15 | 30360.19 | 38983.18 | 46726.04 | 36992.77 | 37677.25 |
| 39310.28 | 33413.49 | 27676.18 | 26812.49 | 38338.92 | 40906.08 |
| 38738.54 | 41216.86 | 24667.89 | 12526.94 | 36690.9 | 37748.72 |
| 31458.45 | 29001.66 | 37636.1 | 37846.97 | 35451.94 | 36487.58 |
| 34191.01 | 24664.8 | 25626.33 | 20976.2 | 35022.66 | 23793.34 |
| 31221.23 | 32849.04 | 40532.12 | 29016.94 | 34199.55 | 41025.31 |
| 31136.53 | 26833.13 | 41864.12 | 29445.3 | 36045.45 | 36658.31 |
| 41145.15 | 61776.89 | 79435.75 | 48207.6 | 17578.5 | 22048 |
| 26021.67 | 15837 | 31274.08 | 23798.51 | 29301.89 | 16193.01 |
| 29223.17 | 24070.3 | 32600.85 | 30294.23 | 30911.69 | 26208.74 |
| 28889.53 | 29301.95 | 32548.85 | 30609.99 | 30297.69 | 31525.16 |
| 27457.04 | 20352.46 | 23098.43 | 20911.74 | 31274.18 | 36340.41 |
| 27976.25 | 25492.63 | 26877.85 | 25149.6 | 30833.69 | 33013.41 |
| 33149.59 | 24767.15 | 40717.35 | 35341.89 | 30973.2 | 26670.5 |
| 31347.1 | 30655.43 | 28857 | 33921.13 | 31329.08 | 25070 |
| 46027.92 | 36368.27 | 31258 | 73747.34 | 35378.5 | 26469.5 |
| 25924.26 | 32250.02 | 28317.7 | 15314.22 | 29919.72 | 32269.48 |
| 29095.28 | 25600.74 | 26344.43 | 26247.35 | 29262.92 | 28450.08 |
| 25232.38 | 28536.55 | 25600.37 | 16278.74 | 28668.55 | 28244.14 |
| 24867.73 | 15927.93 | 27375.85 | 25489.33 | 24447.63 | 20054.24 |
| 27907.5 | 26668.72 | 19338.43 | 27993.83 | 25643.53 | 27317.24 |
| 27254.67 | 22820.8 | 26049.18 | 33068.84 | 25981.52 | 24489.08 |
| 19719.85 | 12625.61 | 26887.28 | 24626.98 | 21329.05 | 14656.15 |
| 24566.33 | 31404.96 | 17688.93 | 20460.47 | 26449.85 | 28288.49 |
| 27177.84 | 22363.12 | 14911.1 | 16158.6 | 26006.42 | 30495.33 |
| 29207.4 | 18661.29 | 25644.45 | 31662.78 | 24571.71 | 21576.06 |
| 22368.12 | 14503.8 | 26898.1 | 24734.85 | 21801.88 | 16482.99 |
| 23729.5 | 22716.89 | 24554.6 | 22763.59 | 24371.85 | 23288.74 |
| 23853.8 | 21386.35 | 24610.1 | 24142.53 | 24283.19 | 22705.24 |
| 23638.5 | 21562.39 | 24377.85 | 22485.34 | 24250.85 | 22946.74 |
| 23628.5 | 21559.39 | 24377.85 | 22476.34 | 24237.85 | 22937.74 |
| 24436.39 | 25520.95 | 9295.71 | 37236.52 | 20365.33 | 22075.3 |
| 18234.36 | 18073.98 | 26223.68 | 24506.37 | 23488.85 | 24435.49 |
| 26593.84 | 20334.12 | 12760.85 | 14997.6 | 23193.92 | 25731.66 |
| 20813.45 | 10520.85 | 17653 | 15619.13 | 20126.33 | 11880.67 |
| 20811.78 | 10520.85 | 17653 | 15619.13 | 20126.33 | 11880.67 |
| 20813 | 17487.58 | 23714.18 | 30301.37 | 20415.19 | 21216.66 |
| 20255.57 | 21493.08 | 22837.45 | 11267.59 | 19467.11 | 21576.07 |
| 18767.88 | 19217.94 | 21407.68 | 10627.26 | 17411.2 | 14618.07 |
| 19964.28 | 18178.91 | 20732.68 | 22692.23 | 18026.78 | 14695.57 |
| 19289.47 | 17308.45 | 24383.77 | 15083.03 | 18153.85 | 13147.65 |
| 14032.44 | 14718.16 | 16875.82 | 26548.52 | 16633.99 | 19259.26 |

| 15901.23 | 12993.7 | 22040.83 | 24575.47 | 16124.17 | 10430.17 |
| --- | --- | --- | --- | --- | --- |
| 20685.86 | 17169.07 | 12592.85 | 13439.19 | 18212.92 | 19098.66 |
| 17138.15 | 22583.15 | 4230.75 | 7172.26 | 16429.91 | 14866.25 |
| 15727.07 | 13993.78 | 13734.42 | 21537.2 | 15833 | 13727.17 |
| 11133.62 | 6620.05 | 18380 | 10406.47 | 15455 | 7690 |
| 10434.12 | 13217.19 | 13654.5 | 6179.12 | 13434.25 | 14012.1 |
| 16834.86 | 17857.53 | 18681.87 | 6375.1 | 16274.28 | 19172.65 |
| 18109.67 | 9103.7 | 6710.51 | 11737.72 | 15045.41 | 8870.44 |
| 13975.11 | 6952.9 | 16059.75 | 13552.4 | 13432.5 | 7910.5 |
| 16656.94 | 12234.61 | 5527.19 | 23363.63 | 14727.07 | 14132.31 |
| 13295.45 | 9535.95 | 15723.25 | 21106.1 | 13239 | 11504.67 |
| 12031.11 | 17799.98 | 12994.1 | 15876.8 | 10568.52 | 8190.99 |
| 13176.85 | 15889.8 | 7964.52 | 2936.77 | 12161.09 | 16006.91 |
| 13173.65 | 15884.35 | 7962.52 | 2936.77 | 12158.09 | 16006.91 |
| 8459.01 | 11018.47 | 18649.35 | 5748.82 | 10930.44 | 10273.49 |
| 8358.01 | 9860.97 | 18472.6 | 5461.57 | 10796.44 | 9922.49 |
| 9602.21 | 7128.87 | 7904.58 | 2709.11 | 11076.33 | 12126.84 |
| 5968.74 | 9312.96 | 4244.85 | 3083.42 | 8832.03 | 10362.08 |
| 11893.34 | 12112.04 | 9667.02 | 4749.7 | 9430.84 | 12770.66 |
| 11232.09 | 11603.92 | 5259.25 | 12426.41 | 11081.58 | 12945.34 |
| 12701.45 | 7504.2 | 13573 | 19936.1 | 10413.5 | 6732 |
| 12701.45 | 7503.95 | 13573 | 19936.1 | 10413.5 | 6732 |
| 12701.45 | 7503.95 | 13573 | 19936.1 | 10413.5 | 6732 |
| 12701.45 | 7503.95 | 13573 | 19936.1 | 10413.5 | 6732 |
| 12701.45 | 7503.95 | 13573 | 19936.1 | 10413.5 | 6732 |
| 12701.45 | 7503.95 | 13573 | 19936.1 | 10413.5 | 6732 |
| 9981.39 | 11056.89 | 4532.75 | 5519.32 | 10748.25 | 13403.25 |
| 9982.39 | 11056.89 | 4532.75 | 5519.32 | 10748.25 | 13403.25 |
| 8175.92 | 7204.6 | 16130.35 | 5430.67 | 10409.19 | 9093.24 |
| 11089.1 | 9543.93 | 2646.86 | 3421.5 | 10450.98 | 8318.98 |
| 7612.25 | 5332.5 | 7947.5 | 4542.34 | 7463.75 | 6983.25 |
| 10832 | 8832.25 | 1728 | 813.28 | 9097 | 12526 |
| 10832 | 8832.25 | 1728 | 813.28 | 9097 | 12526 |
| 10165.27 | 5637.48 | 5448.43 | 14100.83 | 7812.69 | 7794.24 |
| 10219 | 7815.75 | 1332.75 | 565.21 | 9076 | 12456 |
| 8036.41 | 8855.37 | 1913.1 | 1610.43 | 7345.19 | 6507.74 |
| 8875.29 | 8730.29 | 11547.25 | 6101.97 | 5692.08 | 7183.08 |
| 6437.04 | 7268.32 | 3801.5 | 3053.33 | 6426.32 | 4046.09 |
| 6108.45 | 8231.7 | 242.75 | 1123.01 | 6105.9 | 8339.5 |
| 5048.92 | 3923.25 | 863.75 | 612.78 | 4969.5 | 7771.67 |
| 4610.66 | 7090.88 | 837.43 | 2290.34 | 4460.78 | 7111.99 |
| 975.84 | 1774.59 | 2619 | 940.67 | 3498 | 5535.84 |
| 5786.14 | 3132.89 | 276.25 | 902 | 6904 | 9318 |
| 7584.92 | 8215.04 | 9428.5 | 5338.72 | 4571.33 | 6128.08 |
| 7584.92 | 8214.79 | 9427.5 | 5338.72 | 4571.33 | 6128.08 |
| 4114.81 | 2614.42 | 1294.17 | 3190.14 | 4291.12 | 2427.41 |
| 5907.98 | 3165.05 | 168 | 1558.41 | 4981 | 6633 |
| 1459.7 | 1365.28 | 5249 | 1231.71 | 3193.5 | 1463 |
| 2082.76 | 2764.92 | 2284.25 | 2380.67 | 2847.75 | 5054.42 |
| 5081.21 | 4388.5 | 541.75 | 1480.38 | 3150.25 | 4685.75 |
| 4896.43 | 6066.17 | 8454.83 | 2567.85 | 3775.06 | 3268 |
| 3026.92 | 2250.42 | 3134.5 | 2036 | 3738.25 | 5300.42 |
| 5021.26 | 3484.64 | 518.5 | 1260.57 | 3124.25 | 4652.75 |
| 4560.87 | 5565.95 | 1563.67 | 1290 | 3852.67 | 1939.67 |
| 2108.06 | 2288.14 | 2004.5 | 1826.34 | 2619.65 | 5131.92 |
| 2109.06 | 2287.89 | 2005.5 | 1826.34 | 2619.15 | 5131.92 |
| 5055.59 | 3527.39 | 530 | 1262.57 | 3129.25 | 4672.75 |
| 1964.82 | 3609.15 | 2942 | 2641.55 | 1352.33 | 768.33 |

| 3654.13 | 2723.53 | 359 | 1786.85 | 3676 | 4330 |
| --- | --- | --- | --- | --- | --- |
| 559.17 | 1501.17 | 2144 | 779.5 | 2779.5 | 4754.67 |
| 2961 | 2028 | 71 | 50 | 3007 | 3903 |
| 192.23 | 3766.39 | 211.5 | 1001.59 | 220.5 | 395.5 |
| 372.14 | 1427.64 | 2078 | 742.4 | 2597 | 4152.5 |
| 3019.78 | 5844.88 | 6717 | 1901.63 | 561.25 | 1422 |
| 3658.95 | 2156.13 | 2130.53 | 759.79 | 1255.93 | 1012.47 |
| 1910.54 | 2259.37 | 2881.75 | 2320.91 | 1316.83 | 743.83 |
| 85.28 | 2344.78 | 92.75 | 1163.21 | 62.5 | 44 |
| 4096 | 2102.25 | 1208.75 | 9469.5 | 1367.75 | 1584.5 |
| 4096 | 2102.25 | 1208.75 | 9469.5 | 1367.75 | 1584.5 |
| 998.89 | 715.97 | 425 | 207.57 | 768 | 1521 |
| 624.07 | 170.9 | 523 | 566.17 | 711.5 | 537.5 |
| 45.78 | 897.28 | 42.25 | 217.07 | 37 | 30 |
| 917.26 | 1078.74 | 208.94 | 252.06 | 321.46 | 413.74 |
| 366 | 3486 | 208 | 139 | 117 | 423 |
| 494.72 | 204.22 | 211.17 | 311 | 576.33 | 507 |
| 66 | 2992 | 30.25 | 701.07 | 49 | 23 |
| 73.95 | 1765.86 | 34.25 | 413.81 | 40 | 38 |
| 41.78 | 896.28 | 39 | 207.07 | 26 | 23 |
| 75.03 | 1338.77 | 31.5 | 334.01 | 40.5 | 21.5 |
| 119 | 99.25 | 179.25 | 86 | 266.5 | 101 |
| 117 | 889.75 | 51.5 | 217 | 50.5 | 21 |
| 171.67 | 55.67 | 59 | 3.67 | 134 | 580.67 |
| 620 | 1017.5 | 395.25 | 249.07 | 23 | 72 |
| 613 | 1016.5 | 395.25 | 248.07 | 21 | 70 |
| 613 | 1016.5 | 395.25 | 248.07 | 21 | 70 |
| 613 | 1016.5 | 395.25 | 248.07 | 21 | 70 |
| 613 | 1016.5 | 395.25 | 248.07 | 21 | 70 |
| 613 | 1016.5 | 395.25 | 248.07 | 21 | 70 |
| 613 | 1016.5 | 395.25 | 248.07 | 21 | 70 |
| 613 | 1016.5 | 395.25 | 248.07 | 21 | 70 |
| 613 | 1016.5 | 395.25 | 248.07 | 21 | 70 |
| 613 | 1016.5 | 395.25 | 248.07 | 21 | 70 |
| 613 | 1016.5 | 395.25 | 248.07 | 21 | 70 |
| 613 | 1016.5 | 395.25 | 248.07 | 21 | 70 |
| 613 | 1016.5 | 395.25 | 248.07 | 21 | 70 |
| 613 | 1016.5 | 395.25 | 248.07 | 21 | 70 |
| 613 | 1016.5 | 395.25 | 248.07 | 21 | 70 |
| 1 | 70 | 39 | 0.012 | 0.012 | 0.012 |
| 307 | 42 | 129 | 503 | 317 | 110 |
| 307 | 42 | 129 | 503 | 317 | 110 |
| 511.83 | 74.66 | 99 | 42.67 | 242 | 45 |
| 27 | 432 | 16 | 113.14 | 18 | 15 |
| 45 | 21 | 69 | 318 | 12 | 13 |
| 45 | 21 | 69 | 318 | 12 | 13 |
| 11 | 4 | 2 | 9 | 13 | 9 |
| 10 | 3 | 0.012 | 9 | 13 | 9 |
| 10 | 3 | 0.012 | 9 | 13 | 9 |
| 10 | 3 | 0.012 | 9 | 13 | 9 |
| 10 | 3 | 0.012 | 9 | 13 | 9 |
| 10 | 3 | 0.012 | 9 | 13 | 9 |
| 10 | 3 | 0.012 | 9 | 13 | 9 |
| 11.25 | 13.25 | 5.75 | 2 | 3 | 1 |
| 3.2 | 5.7 | 3 | 0.012 | 3 | 0.012 |
| 3.2 | 5.7 | 3 | 0.012 | 3 | 0.012 |
| 3.2 | 5.7 | 3 | 0.012 | 3 | 0.012 |
| 3.2 | 5.7 | 3 | 0.012 | 3 | 0.012 |

| 3.2 | 5.7 | 3 | 0.012 | 3 | 0.012 |
| --- | --- | --- | --- | --- | --- |
| 5 | 2.25 | 3 | 7 | 0.012 | 1 |
| 2 | 1 | 0.012 | 0.012 | 0.012 | 0.012 |
| 12.33 | 7.75 | 14.25 | 0.012 | 0.012 | 0.012 |
| 1 | 4.25 | 0.012 | 8.07 | 2 | 0.012 |
| 0.5 | 4.33 | 2 | 0.67 | 0.012 | 0.012 |
| 1 | 2.5 | 1 | 0.012 | 0.012 | 0.012 |
| 0.012 | 2 | 1 | 0.012 | 0.012 | 0.012 |
| 20 | 2.5 | 2 | 0.012 | 0.012 | 8 |
| 0.012 | 0.012 | 2 | 47 | 1 | 0.012 |
| 6 | 12 | 0.012 | 0.012 | 0.012 | 0.012 |
| 0.012 | 12 | 0.012 | 0.012 | 0.012 | 0.012 |
| 0.012 | 0.012 | 5 | 0.012 | 0.012 | 0.012 |
| 1 | 1 | 2 | 0.012 | 0.012 | 0.012 |
| 0.012 | 0.2 | 0.25 | 0.012 | 0.012 | 0.012 |
| 0.012 | 0.25 | 1 | 0.012 | 0.012 | 0.012 |
| 0.012 | 0.012 | 0.012 | 0.012 | 0.012 | 0.012 |
| 1 | 0.012 | 0.012 | 0.012 | 0.012 | 0.012 |
| 0.012 | 0.012 | 0.012 | 0.012 | 0.012 | 0.012 |
| 0.012 | 1 | 0.012 | 0.012 | 0.012 | 0.012 |
| 0.012 | 0.012 | 0.012 | 0.012 | 0.012 | 0.012 |
| 0.012 | 0.012 | 0.012 | 0.5 | 0.012 | 0.012 |
| 0.012 | 0.012 | 0.012 | 0.012 | 0.012 | 0.012 |
| 0.012 | 0.012 | 0.012 | 0.012 | 0.012 | 0.012 |
| 0.012 | 0.012 | 0.012 | 0.012 | 0.012 | 0.012 |
| 0.012 | 0.012 | 0.012 | 0.012 | 0.012 | 0.012 |
| 0.012 | 1 | 0.012 | 0.012 | 0.012 | 0.012 |
| 0.012 | 0.012 | 1 | 0.012 | 0.012 | 0.012 |
| 0.012 | 0.25 | 0.012 | 0.012 | 0.012 | 0.012 |
| 0.012 | 0.012 | 0.012 | 0.012 | 0.012 | 0.012 |
| 0.012 | 0.012 | 1 | 7.07 | 0.012 | 0.012 |
| 0.012 | 0.012 | 0.012 | 0.012 | 0.012 | 0.012 |
| 0.012 | 0.25 | 0.012 | 0.012 | 0.012 | 0.012 |
| 0.012 | 0.012 | 0.012 | 0.012 | 0.012 | 0.012 |
| 1 | 0.5 | 0.012 | 0.012 | 0.012 | 0.012 |
| 0.012 | 0.012 | 0.012 | 0.012 | 0.012 | 0.012 |
| 0.012 | 1 | 0.012 | 0.012 | 0.012 | 0.012 |
| 0.012 | 0.012 | 0.012 | 0.012 | 0.012 | 0.012 |
| 0.012 | 0.012 | 0.012 | 0.012 | 0.012 | 0.012 |
| 0.012 | 0.012 | 0.012 | 0.012 | 0.012 | 0.012 |
| 0.012 | 0.012 | 0.012 | 0.012 | 0.012 | 0.012 |
| 0.012 | 0.012 | 0.012 | 0.012 | 0.012 | 0.012 |
| 0.012 | 0.012 | 0.012 | 0.012 | 0.012 | 0.012 |
| 0.012 | 0.012 | 0.012 | 0.012 | 0.012 | 0.012 |
| 0.012 | 0.012 | 0.012 | 0.012 | 0.012 | 0.012 |
| 0.012 | 0.012 | 0.012 | 0.012 | 0.012 | 0.012 |
| 0.012 | 0.012 | 0.012 | 0.012 | 0.012 | 0.012 |
| 0.012 | 0.012 | 0.012 | 0.012 | 0.012 | 0.012 |
| 0.012 | 0.012 | 0.012 | 0.012 | 0.012 | 0.012 |
| 0.012 | 0.012 | 0.012 | 0.012 | 0.012 | 0.012 |
| 0.012 | 0.012 | 0.012 | 0.012 | 0.012 | 0.012 |
| 0.012 | 0.012 | 0.012 | 0.012 | 0.012 | 0.012 |
| 0.012 | 0.012 | 0.012 | 0.012 | 0.012 | 0.012 |
| 0.012 | 0.012 | 0.012 | 0.012 | 0.012 | 0.012 |
| 0.012 | 0.012 | 0.012 | 0.012 | 0.012 | 0.012 |
| 0.012 | 0.012 | 0.012 | 0.012 | 0.012 | 0.012 |
| 0.012 | 0.012 | 0.012 | 0.012 | 0.012 | 0.012 |
| 0.012 | 0.012 | 0.012 | 0.012 | 0.012 | 0.012 |

| 0.012 | 0.012 | 0.012 | 0.012 | 0.012 | 0.012 |
| --- | --- | --- | --- | --- | --- |
| 0.012 | 0.012 | 0.012 | 0.012 | 0.012 | 0.012 |
| 0.012 | 0.012 | 0.012 | 0.012 | 0.012 | 0.012 |
| 0.012 | 0.012 | 0.012 | 0.012 | 0.012 | 0.012 |
| 0.012 | 0.012 | 0.012 | 0.012 | 0.012 | 0.012 |
| 0.012 | 0.012 | 0.012 | 0.012 | 0.012 | 0.012 |
| 0.012 | 0.012 | 0.012 | 0.012 | 0.012 | 0.012 |
| 0.012 | 0.012 | 0.012 | 0.012 | 0.012 | 0.012 |
| 0.012 | 0.012 | 0.012 | 0.5 | 0.012 | 0.012 |
| 1 | 0.5 | 0.012 | 0.012 | 0.012 | 0.012 |
| 1 | 0.5 | 0.012 | 0.012 | 0.012 | 0.012 |
| 0.012 | 0.012 | 0.012 | 0.012 | 0.012 | 0.012 |
| 1 | 0.012 | 0.012 | 0.012 | 0.012 | 0.012 |
| 0.012 | 0.012 | 1 | 0.012 | 0.012 | 0.012 |
| 0.012 | 0.012 | 1 | 0.012 | 0.012 | 0.012 |
| 0.012 | 0.012 | 0.012 | 0.012 | 0.012 | 0.012 |
| 0.012 | 0.2 | 0.012 | 0.012 | 0.012 | 0.012 |
| 0.012 | 0.012 | 0.012 | 0.012 | 0.012 | 0.012 |

| EAC_7 | Control_1 | Control_5 | Control_6 | Control_7 | Control_8 |
| --- | --- | --- | --- | --- | --- |
| 8671839.98 | 8021863.63 | 7832389.54 | 7957334.34 | 7958789.67 | 7760064.58 |
| 4170475.26 | 3804853.87 | 3734671.86 | 3837688.05 | 3738357.91 | 3571191.2 |
| 2037519.49 | 1895582.12 | 1831384.51 | 1905389.34 | 1750634.82 | 1786547.62 |
| 1830890.04 | 1584753.9 | 1620965.07 | 1608501.63 | 1497922.96 | 1422726.58 |
| 1239190.33 | 1197834.43 | 1121836.29 | 1186011.78 | 1161665.2 | 1130128.14 |
| 1201204.87 | 1133885.44 | 1126385.84 | 1148806.94 | 1156055.13 | 1124694.36 |
| 954198.4 | 1006480.96 | 971285.43 | 906503.34 | 792396.95 | 1101174.28 |
| 815432.71 | 771074.34 | 747598.44 | 799094.22 | 759393.52 | 762269.15 |
| 635073.16 | 581063.95 | 622115.47 | 594615.68 | 550483.37 | 609649.69 |
| 602539.1 | 557320.77 | 563566.48 | 564268.97 | 599634.87 | 566265.27 |
| 558511.1 | 513355.32 | 521138.25 | 519088.15 | 524309.92 | 509449.24 |
| 534228.66 | 486489.04 | 501730.6 | 539776.98 | 528982.5 | 527279.56 |
| 536557.42 | 480247.24 | 490538.46 | 521817.26 | 517045.25 | 473811.84 |
| 541530.6 | 469677.59 | 474783.14 | 472195.2 | 500534.81 | 425163.54 |
| 477891.83 | 477401.69 | 424230.66 | 457171.73 | 451310.94 | 392700.36 |
| 443835.21 | 499764.21 | 431257.77 | 465269.86 | 411466.98 | 484094.17 |
| 464390.01 | 469731.64 | 427296.66 | 450045.14 | 427224.65 | 444507.8 |
| 459550.81 | 433577.55 | 426820.54 | 443518.57 | 447129.43 | 435764.95 |
| 414754.61 | 384090.03 | 376100.02 | 404080.81 | 410150.18 | 365854.76 |
| 444523.95 | 386934.93 | 427720.22 | 454744.14 | 410350.47 | 382713.45 |
| 417681.93 | 396781.17 | 392665.3 | 405180.13 | 404093.63 | 387605.57 |
| 395678.65 | 379150.2 | 356899.53 | 357865.13 | 402179.05 | 347250.69 |
| 381964.5 | 372894.64 | 357993.76 | 372835.76 | 381313.33 | 371026.56 |
| 468292.72 | 338702.22 | 400745.45 | 297037.77 | 323027.35 | 340361.22 |
| 379533.24 | 349180.92 | 343072.56 | 355221.76 | 374300.04 | 313439.54 |
| 392755.55 | 332335.24 | 343546.23 | 333102.3 | 325906.69 | 301608.85 |
| 354207.23 | 327322.21 | 334742.11 | 334527.04 | 297995.38 | 308944.06 |
| 324253.98 | 311018.92 | 302500.92 | 326505.2 | 308381.37 | 307497.72 |
| 315895.73 | 298279.26 | 288552.38 | 300883.01 | 309979.11 | 305649.37 |
| 296368.06 | 292112.37 | 253946.56 | 279538.75 | 292235.77 | 249119.56 |
| 291643.31 | 329330.88 | 269928.18 | 261757.47 | 248621.53 | 299674.22 |
| 301734.58 | 276299.65 | 278554.42 | 280868.34 | 268454.89 | 278628.02 |
| 303041.3 | 278077.16 | 266513.55 | 276895.81 | 267852.99 | 242898.67 |
| 305178.18 | 271170.39 | 288621.56 | 330618.16 | 306483.53 | 284898.58 |
| 309310.93 | 276451.07 | 263769.72 | 253160.55 | 218640.56 | 235411.53 |
| 269382.65 | 253646.29 | 253710.13 | 244277.21 | 254574.14 | 244068.64 |
| 283322.64 | 257704.8 | 242382.67 | 214232.93 | 248966.25 | 257201.08 |
| 268254.12 | 249374.4 | 227665.9 | 151772.75 | 258055 | 260409.45 |
| 266515.4 | 251161.9 | 246117.65 | 252298.11 | 247687.13 | 255326.81 |
| 286079.78 | 252614.97 | 261699.09 | 261941.39 | 242228.88 | 229721.03 |
| 261197.95 | 236093.94 | 239768.49 | 249325.14 | 272562.14 | 228929.12 |
| 227665.63 | 255372.52 | 208743.9 | 220811.38 | 223193.23 | 258130.06 |
| 271499.22 | 245819.4 | 242213.98 | 244261.75 | 237262.35 | 234391.94 |
| 281266.02 | 226172.42 | 269317.99 | 278987.4 | 270589.8 | 244474.77 |
| 260440.71 | 231284.09 | 231108.99 | 229326.59 | 247340.75 | 239383.03 |
| 259583.59 | 256737.35 | 238924.12 | 267777.15 | 209473.61 | 230898.71 |
| 221470.24 | 254599.82 | 207467.73 | 212615.63 | 220303.16 | 250511.96 |
| 245460.98 | 224222.4 | 227298.22 | 238092.99 | 237277.04 | 229705.91 |
| 235123.49 | 221602.43 | 219481.65 | 218200.97 | 231061.8 | 219034.46 |
| 213283.39 | 228761.65 | 197194.11 | 211123.91 | 214296.54 | 249503.94 |
| 214828.74 | 212144.91 | 196908.46 | 218825.93 | 226366.94 | 206584.85 |
| 209454.26 | 230545.12 | 201160.47 | 196722.28 | 207112.89 | 194313.87 |
| 180368.26 | 195709.85 | 175149.38 | 207227.61 | 219899.95 | 205912.18 |
| 203151.02 | 194834.03 | 188232.47 | 212794.23 | 208762.66 | 187750.13 |
| 200237.2 | 172491.75 | 181192.71 | 186268.41 | 186666.79 | 173972.16 |
| 221378.24 | 230776.45 | 250730.44 | 292331.21 | 136135.17 | 242313.39 |
| 206853.35 | 163326.48 | 173781.01 | 142865.12 | 178755.6 | 144906.74 |

| 189799.38 | 173324.45 | 167499.7 | 151373.03 | 127416.77 | 166662.86 |
| --- | --- | --- | --- | --- | --- |
| 181264.94 | 165809.9 | 169933.89 | 169481.5 | 169583.73 | 151888.22 |
| 167571.58 | 153379.63 | 155585.79 | 163548.5 | 170431.56 | 155211.55 |
| 138148.88 | 174688.07 | 128514.54 | 132978.5 | 125496.36 | 186979.25 |
| 179006.9 | 153463.24 | 151162.68 | 148485.42 | 141298.2 | 122281.26 |
| 161316.91 | 144217.37 | 152425.79 | 142153.94 | 151292.95 | 146975.5 |
| 141442.78 | 155676.57 | 122430.52 | 132467.06 | 129200.83 | 159288.85 |
| 156466.9 | 150254.72 | 135099.73 | 130764.02 | 134417.97 | 124566.16 |
| 129202.8 | 118022.17 | 123143.95 | 124080.35 | 129004.66 | 129402.81 |
| 116536.79 | 115944.87 | 109445.25 | 119224.87 | 123998.4 | 103729.39 |
| 115836.47 | 131643.87 | 102418.95 | 131215.84 | 106771.42 | 133669.22 |
| 145522.44 | 116626.34 | 115188.77 | 129609 | 112325.14 | 82942.17 |
| 128033.38 | 121794.88 | 121679.02 | 138664.65 | 117946.79 | 119413.54 |
| 124349.47 | 112064.03 | 114467.53 | 126381.33 | 110902.02 | 114847.13 |
| 98390.69 | 128621.68 | 93482.28 | 98649.6 | 106359.78 | 126916 |
| 100839.93 | 106152.01 | 94650.68 | 81689.39 | 128316.17 | 105376.14 |
| 90047.72 | 100506.88 | 96098.33 | 95742.72 | 106472.66 | 129948.83 |
| 115981.46 | 110652.63 | 106139.52 | 95292.22 | 88691.09 | 104278.41 |
| 118830.35 | 114632.88 | 100175.41 | 119404.48 | 103429.42 | 102798.79 |
| 111276.71 | 102243.91 | 101596.53 | 102428.96 | 101418.5 | 96920.64 |
| 108098.8 | 100170.3 | 99026.27 | 103943.95 | 111004.56 | 97251.92 |
| 101854.43 | 101962.68 | 90144.59 | 88571.35 | 103168.63 | 109942.99 |
| 107691.78 | 98132.86 | 103512.52 | 113057.1 | 96136.64 | 110014.85 |
| 95840.59 | 94757.92 | 94359.27 | 100112.98 | 73581.95 | 119359.06 |
| 111594.98 | 86454.51 | 92916.95 | 126657.97 | 73687.95 | 84973.23 |
| 96113.99 | 61525.15 | 76562.33 | 87701.86 | 142116.86 | 82858.28 |
| 92915.34 | 94049.14 | 83156.52 | 94985.46 | 78850.06 | 92388.93 |
| 92032.77 | 85780.14 | 85961.52 | 100188.6 | 78645.29 | 98181.04 |
| 79470.4 | 80482.38 | 71442.76 | 82938.56 | 88618.85 | 79912.98 |
| 88802.18 | 84613.39 | 83854.86 | 84927.54 | 79271.98 | 82706.16 |
| 85127.78 | 72845.43 | 69109.18 | 80893.89 | 75492.77 | 76426.9 |
| 82643.36 | 64041.76 | 75800.43 | 75309.9 | 78136.23 | 62829.75 |
| 78083 | 69193.43 | 71964.69 | 68461.97 | 71811.19 | 70928.12 |
| 64241.14 | 70175.96 | 57668.43 | 92466.92 | 56751.66 | 70490.38 |
| 74344.72 | 68166.1 | 64681.09 | 69176.39 | 75366.61 | 61719.04 |
| 72440.96 | 63435.11 | 61979.19 | 84201.15 | 63278.15 | 63473.36 |
| 61495.65 | 56899.84 | 59613.75 | 61697.39 | 70828.63 | 56932.66 |
| 71384.37 | 56905.04 | 59998.76 | 71387.73 | 77619.66 | 59592.18 |
| 68565.78 | 76059.19 | 56928.6 | 80175.82 | 58922.93 | 59021.39 |
| 79030.31 | 62264.2 | 69118.66 | 65910.2 | 64660.43 | 59513.24 |
| 71734.97 | 60628.36 | 63306.35 | 64670.29 | 67869.72 | 58149.93 |
| 76541.23 | 60600.87 | 59341.35 | 71982.31 | 60902.59 | 49578.04 |
| 65283.36 | 65559.45 | 58498.6 | 62621.73 | 60186.13 | 55162.36 |
| 63796.47 | 60620.44 | 55923.58 | 56835.57 | 54402.2 | 57754.29 |
| 50246.94 | 64571.37 | 50013.34 | 55550.63 | 59303.73 | 63313.93 |
| 65316.15 | 40387.16 | 49157.67 | 58327.1 | 92812.26 | 54432.39 |
| 51915.26 | 65209.78 | 45339.42 | 85009.99 | 35741.54 | 53346.66 |
| 61870.73 | 54149.67 | 58815.27 | 59265.39 | 55800.84 | 59123.49 |
| 67677 | 55291.38 | 53355.27 | 54552.31 | 57545.12 | 48007.02 |
| 61126.72 | 50952.18 | 52678.26 | 53108.21 | 52397.01 | 48253.47 |
| 55036.61 | 52346.16 | 52969.18 | 60957.8 | 59068.16 | 51036.15 |
| 54635.84 | 44604.47 | 49849.76 | 51378.14 | 59577.33 | 49900.49 |
| 54633.96 | 33171.07 | 44747.16 | 52803.25 | 91045.1 | 44993.72 |
| 54823.35 | 38454.48 | 45268.68 | 49214.48 | 58833.43 | 41672.91 |
| 55896.31 | 42722.19 | 51495.34 | 53275.57 | 47259.84 | 41152.82 |
| 50160.9 | 44528.48 | 39463.01 | 46101.32 | 46406.84 | 37030.33 |
| 56716.94 | 47089.37 | 45862.01 | 52018.23 | 41981.79 | 37515.7 |
| 53755.43 | 41840.49 | 42155.35 | 41554.99 | 49710.98 | 36775.67 |

| 36328.73 | 45966.71 | 33619.09 | 41210.07 | 45458.12 | 41273.04 |
| --- | --- | --- | --- | --- | --- |
| 52252.9 | 32954.81 | 40276.51 | 43395.82 | 47271.17 | 34633.5 |
| 38436.95 | 41548.55 | 40828.51 | 38941.51 | 34135.28 | 60041.55 |
| 39871.73 | 44200.23 | 34554.67 | 44535.74 | 31312.41 | 49910.43 |
| 45935.45 | 33715.22 | 41141.68 | 37226.14 | 45071.9 | 34312.25 |
| 53851.65 | 42427.07 | 57834.13 | 51293.4 | 34473.21 | 34471.11 |
| 43855.32 | 32567.67 | 33946.26 | 44604.49 | 36497.53 | 31971.31 |
| 33728.49 | 21386 | 37262.51 | 22845.82 | 35888.51 | 31574.15 |
| 32463.69 | 44368.71 | 33197.59 | 39722.82 | 29785.71 | 43111.95 |
| 34800.94 | 37942.81 | 32425.18 | 25930.15 | 38309.82 | 33080.33 |
| 28264.91 | 44111.71 | 28711.09 | 31442.57 | 28152.12 | 43232.37 |
| 42868.18 | 32642.22 | 44170.59 | 34914.92 | 32980.82 | 30666.83 |
| 37030.18 | 34383.97 | 35148.51 | 33509.99 | 31161.12 | 33222.71 |
| 48920.2 | 34877.06 | 40627.18 | 46187.56 | 27613.9 | 29048.76 |
| 31116.95 | 29958.46 | 30229 | 34297.32 | 35193.94 | 30359.54 |
| 38823.58 | 39476.96 | 33130.17 | 36765.41 | 20969.63 | 34242.03 |
| 37261.8 | 28388.22 | 33378.92 | 35353.15 | 37513.4 | 30817.6 |
| 29916.02 | 40718.14 | 25892.67 | 32531.65 | 24102.82 | 27703.68 |
| 29339.63 | 49099.18 | 28171.91 | 36164.31 | 17539.17 | 25403.83 |
| 37154.8 | 27688.22 | 33037.59 | 35007.49 | 36426.73 | 28384.27 |
| 32871.4 | 27443.83 | 25351.33 | 30287.41 | 47496.17 | 36760.49 |
| 34193.57 | 27917.63 | 29810.42 | 29227.16 | 26685.61 | 28863.72 |
| 29575.29 | 28956.88 | 27489.67 | 33648.16 | 33554.95 | 24609.22 |
| 16297.82 | 9111.25 | 20465.25 | 12565.25 | 24734.5 | 15622 |
| 23534.85 | 19750.41 | 18863.83 | 28293.91 | 51913.17 | 32575.65 |
| 27659.02 | 25435.81 | 25670.34 | 26233.49 | 32270.98 | 26337.68 |
| 29782.95 | 25192.89 | 27754.09 | 28571.24 | 26644.98 | 27267.35 |
| 23134.11 | 29585.56 | 21426.58 | 33640.24 | 21592.33 | 29720.52 |
| 28778.42 | 28782.14 | 25808.84 | 29123.49 | 24044.48 | 27267.35 |
| 25008.55 | 24711.31 | 23638.5 | 21799.57 | 34879.23 | 22563.28 |
| 30115.23 | 25200.92 | 24388.68 | 18692.41 | 31605.25 | 22453.06 |
| 26774.28 | 24359 | 26307.5 | 8858 | 22209.5 | 28344 |
| 32408.38 | 29537.96 | 28903.18 | 29243.48 | 18290.28 | 23040.36 |
| 27557.52 | 27225.14 | 23866.67 | 27510.15 | 23379.82 | 23869.68 |
| 28829.93 | 26451.47 | 25851.01 | 25395.76 | 24619.28 | 21590.88 |
| 19406.35 | 21279.56 | 20030.42 | 27377.74 | 36110.15 | 25959.02 |
| 23905.82 | 23696.89 | 19433.17 | 25015.07 | 21244.81 | 23501.69 |
| 23294.02 | 22552.64 | 22194.67 | 25356.15 | 23217.15 | 24257.18 |
| 18944.25 | 20581.96 | 20222.25 | 28798.48 | 36796.79 | 23926.54 |
| 27706.25 | 24841.48 | 21505.75 | 19610.75 | 14113.98 | 18461.18 |
| 22261.78 | 25241.98 | 18167.17 | 25564.83 | 12655.15 | 26976.19 |
| 12992.54 | 22863.3 | 17413.5 | 20296.66 | 22420.29 | 28509.71 |
| 18503.9 | 18493.56 | 18213.92 | 22886.24 | 34632.56 | 16700.27 |
| 25992.33 | 21319.4 | 21720.92 | 21338.83 | 21632.48 | 20976.35 |
| 22734.02 | 21106.31 | 21278.84 | 21388.49 | 21692.98 | 21008.18 |
| 24313.83 | 21200.65 | 21480.17 | 21319.33 | 21595.98 | 20979.85 |
| 24280.83 | 21177.65 | 21450.17 | 21314.33 | 21584.98 | 20974.85 |
| 11101.07 | 27499.03 | 11046.41 | 32755.74 | 9923.17 | 24416.5 |
| 24904.66 | 19117.9 | 21680.83 | 22500.74 | 19883.65 | 17754.35 |
| 17140.28 | 23277.15 | 14989.67 | 20017.83 | 11038.65 | 25520.02 |
| 14625.23 | 14201.83 | 11577.5 | 17778.25 | 31935.5 | 25011.66 |
| 14624.73 | 14201.83 | 11577.5 | 17778.25 | 31935.5 | 25011.66 |
| 15799.85 | 17166.23 | 15719.25 | 18917.66 | 18936.47 | 20174.02 |
| 15667.24 | 25397.12 | 16055.5 | 24128.66 | 10631.97 | 14854.87 |
| 19450.62 | 17602.81 | 16179.34 | 16579.48 | 17193.14 | 13900.18 |
| 16588.44 | 13282.56 | 16912.67 | 14180.82 | 19304.06 | 19388.77 |
| 17811.55 | 17108.4 | 18926.51 | 17644.16 | 20999.99 | 17105.84 |
| 20434.35 | 13493.83 | 12391.57 | 13883.65 | 17236.51 | 9169.32 |

| 16790.4 | 9793.91 | 13863.5 | 15668.57 | 23906.51 | 10806.66 |
| --- | --- | --- | --- | --- | --- |
| 15852.08 | 17235.65 | 13276.17 | 14414.33 | 9831.81 | 18500.02 |
| 17683.29 | 15258.34 | 16829.34 | 7886.5 | 9381.75 | 15758.57 |
| 17661.67 | 11132.08 | 14358.51 | 13697.08 | 17219.17 | 12159.66 |
| 11885.7 | 8614 | 9725 | 13973 | 29665 | 11959 |
| 17534.45 | 14796.65 | 14705.5 | 19602 | 17048.92 | 14882.1 |
| 10978.85 | 21745.96 | 12588.17 | 16529 | 7730.45 | 11454.88 |
| 8759.85 | 21444.4 | 14067.66 | 26659.72 | 12905.86 | 21173.81 |
| 10116.5 | 8676 | 8792 | 9704 | 21227.5 | 10782.5 |
| 7622.95 | 17256.05 | 6315.49 | 14664.32 | 7683.87 | 13157.97 |
| 13151.34 | 8062.58 | 10194.5 | 14938.75 | 15145.5 | 10300.67 |
| 14458.04 | 10447.82 | 11550.92 | 19430.33 | 8425.66 | 10096.85 |
| 7126.45 | 16826.56 | 6354.17 | 11131.92 | 4251.81 | 6898.69 |
| 7120.31 | 16819.56 | 6354.17 | 11131.92 | 4249.81 | 6898.69 |
| 15199.25 | 8076.65 | 12008.75 | 9241.58 | 14397.39 | 5729.44 |
| 13487.75 | 7934.9 | 11738 | 9217.08 | 14349.89 | 5727.94 |
| 6625 | 13805.83 | 6626.83 | 12049.16 | 11453.34 | 8369 |
| 17296.33 | 9534.14 | 14174.09 | 35111.4 | 6324.64 | 10557.19 |
| 8766.16 | 13290.56 | 7391.17 | 9830.92 | 5155.06 | 5806.44 |
| 11639.11 | 10489.08 | 9403.42 | 9604.5 | 6593.25 | 7667.75 |
| 7996.84 | 6074.75 | 6987 | 9387.75 | 13518 | 8839.5 |
| 7996.84 | 6074.75 | 6987 | 9387.75 | 13518 | 8839.5 |
| 7996.84 | 6074.75 | 6987 | 9387.75 | 13518 | 8839.5 |
| 7996.84 | 6074.75 | 6987 | 9387.75 | 13518 | 8839.5 |
| 7996.84 | 6074.75 | 6987 | 9387.75 | 13518 | 8839.5 |
| 7996.84 | 6074.75 | 6987 | 9387.75 | 13518 | 8839.5 |
| 13222.03 | 10713.41 | 9820.84 | 9351.66 | 5857.09 | 8508.74 |
| 13222.03 | 10713.41 | 9820.84 | 9351.66 | 5857.09 | 8508.74 |
| 8342.69 | 10901.9 | 9884.5 | 11534.08 | 12974.48 | 7208.69 |
| 8227.57 | 14569.3 | 8879.66 | 11757.99 | 6621.96 | 9589.88 |
| 6536.95 | 7415 | 4821.5 | 6439.5 | 10411.25 | 2954.75 |
| 2609.5 | 13510 | 2053 | 5035.5 | 730 | 3851 |
| 2609.5 | 13510 | 2053 | 5035.5 | 730 | 3851 |
| 3444.69 | 7903.48 | 7133.83 | 7382.5 | 6230.31 | 14076.02 |
| 2358.5 | 13493 | 2026 | 5021.5 | 723 | 3834 |
| 7138.05 | 10555.9 | 6594.5 | 7306.58 | 4160.31 | 6260.69 |
| 6147.92 | 4450 | 7861.5 | 4684 | 3084.42 | 5979.58 |
| 9895.8 | 5894.92 | 8896.42 | 4572.33 | 4639.34 | 3570.15 |
| 2546.6 | 9011 | 1365 | 3359 | 481.17 | 2521 |
| 2740.43 | 7786.33 | 2837 | 6533.5 | 1850.5 | 9236.17 |
| 2435.95 | 6891.73 | 2598.33 | 6261.66 | 1862.72 | 6136.45 |
| 9695.5 | 2944.66 | 6322 | 34931.5 | 2215 | 2410.84 |
| 2858.53 | 10211 | 2163 | 2857 | 1032 | 5760 |
| 4532.17 | 3052.75 | 5329.5 | 3624.25 | 2648.17 | 5355.83 |
| 4532.17 | 3051.75 | 5329.5 | 3623.25 | 2648.17 | 5355.83 |
| 2678.72 | 6467.73 | 3108.82 | 7791.24 | 3173.99 | 5725.85 |
| 1288.2 | 6041.5 | 1713.5 | 5603.5 | 1206.84 | 7020 |
| 3181.5 | 1426 | 2953.5 | 4407 | 9265 | 1381 |
| 5586.25 | 1991.83 | 3221.5 | 5631 | 1742.92 | 1498.42 |
| 1263.35 | 4529.5 | 684.5 | 1690 | 297.42 | 1354.75 |
| 4384.28 | 3200.75 | 2649.58 | 3299.66 | 2081.34 | 2320.16 |
| 5388.25 | 2407.33 | 3375.5 | 3134 | 1991.25 | 2007.92 |
| 1124.15 | 4515.5 | 953.5 | 4843 | 284.25 | 1331.25 |
| 5044.17 | 2775.33 | 4838.67 | 1367.33 | 3064.17 | 1740 |
| 4314.95 | 1916.83 | 3030.5 | 5394 | 1537.75 | 1451.42 |
| 4314.95 | 1916.83 | 3030.5 | 5394 | 1538.75 | 1450.42 |
| 925.29 | 4527.5 | 673.5 | 1667 | 285.25 | 1331.25 |
| 2130.57 | 873 | 2206.5 | 10371.5 | 997.18 | 913.33 |

| 808.5 | 4766.5 | 746 | 1867 | 401.85 | 1682 |
| --- | --- | --- | --- | --- | --- |
| 5104.5 | 1986.33 | 3164 | 5547.5 | 1577.5 | 1456.17 |
| 643 | 4457 | 633 | 1633 | 232 | 1242 |
| 4068.5 | 199 | 869.5 | 6418.5 | 58.67 | 39.5 |
| 4479.7 | 1201 | 2582.5 | 1923.5 | 1147 | 643.5 |
| 2508.27 | 387.5 | 1219.25 | 208.75 | 1071.25 | 398.25 |
| 719.53 | 1789.46 | 1792.33 | 978.67 | 1592.6 | 1124.39 |
| 1030.97 | 835 | 1618 | 4005.5 | 981.68 | 908.33 |
| 1714.6 | 52.5 | 589.5 | 6367 | 20.5 | 5 |
| 155.25 | 635.75 | 139.25 | 204 | 974.75 | 2423.25 |
| 155.25 | 635.75 | 139.25 | 204 | 974.75 | 2423.25 |
| 274.2 | 1243 | 745.5 | 537 | 548 | 3553 |
| 606.64 | 2053.5 | 660 | 3118.5 | 348.17 | 1923 |
| 1175.95 | 18 | 305 | 3204 | 8 | 8 |
| 795.26 | 703.4 | 1102.34 | 958.66 | 1139.8 | 1367.86 |
| 2465.33 | 226 | 1347 | 84 | 407 | 64 |
| 557.67 | 1197.67 | 899.67 | 1282.67 | 596.67 | 779 |
| 812 | 21 | 4 | 4 | 14 | 4 |
| 584.4 | 20 | 13 | 25 | 8.17 | 21.5 |
| 680.7 | 15 | 19 | 22.5 | 2 | 6 |
| 749.9 | 28.5 | 44.5 | 13 | 19.01 | 4 |
| 1165.5 | 74.5 | 408.5 | 3321.5 | 85.5 | 59.5 |
| 454 | 22 | 4 | 1 | 1 | 1 |
| 100 | 710.33 | 254 | 434 | 419 | 807.67 |
| 251 | 30 | 29 | 21 | 7 | 21 |
| 251 | 17 | 27 | 14 | 7 | 17 |
| 251 | 17 | 27 | 14 | 7 | 17 |
| 251 | 17 | 27 | 14 | 7 | 17 |
| 251 | 17 | 27 | 14 | 7 | 17 |
| 251 | 17 | 27 | 14 | 7 | 17 |
| 251 | 17 | 27 | 14 | 7 | 17 |
| 251 | 17 | 27 | 14 | 7 | 17 |
| 251 | 17 | 27 | 14 | 7 | 17 |
| 251 | 17 | 27 | 14 | 7 | 17 |
| 251 | 17 | 27 | 14 | 7 | 17 |
| 251 | 17 | 27 | 14 | 7 | 17 |
| 251 | 17 | 27 | 14 | 7 | 17 |
| 251 | 17 | 27 | 14 | 7 | 17 |
| 251 | 17 | 27 | 14 | 7 | 17 |
| 0.012 | 0.012 | 0.012 | 0.012 | 1 | 0.012 |
| 9 | 137 | 47 | 98 | 80 | 205 |
| 9 | 137 | 47 | 98 | 80 | 205 |
| 0.012 | 72 | 145 | 75 | 9.17 | 33 |
| 11 | 45 | 21 | 26 | 4 | 15 |
| 1 | 13 | 64 | 67 | 141 | 31 |
| 1 | 13 | 64 | 67 | 141 | 31 |
| 33 | 23 | 30 | 4 | 12 | 5 |
| 33 | 23 | 30 | 4 | 11 | 5 |
| 33 | 23 | 30 | 4 | 11 | 5 |
| 33 | 23 | 30 | 4 | 11 | 5 |
| 33 | 23 | 30 | 4 | 11 | 5 |
| 33 | 23 | 30 | 4 | 11 | 5 |
| 33 | 23 | 30 | 4 | 11 | 5 |
| 44 | 24 | 8 | 5 | 10 | 7 |
| 6.14 | 8 | 0.012 | 1 | 2 | 0.012 |
| 6.14 | 8 | 0.012 | 1 | 2 | 0.012 |
| 6.14 | 8 | 0.012 | 1 | 2 | 0.012 |
| 6.14 | 8 | 0.012 | 1 | 2 | 0.012 |

| 6.14 | 8 | 0.012 | 1 | 2 | 0.012 |
| --- | --- | --- | --- | --- | --- |
| 0.012 | 0.012 | 2 | 2 | 5 | 2 |
| 0.012 | 0.012 | 0.012 | 0.012 | 0.012 | 0.012 |
| 0.34 | 0.012 | 1 | 0.012 | 0.012 | 0.012 |
| 8 | 6 | 1 | 0.012 | 3 | 0.012 |
| 0.012 | 0.012 | 0.012 | 0.012 | 0.17 | 0.012 |
| 0.012 | 0.012 | 0.012 | 0.012 | 0.012 | 0.012 |
| 0.012 | 0.012 | 0.012 | 0.012 | 0.012 | 0.012 |
| 6 | 1 | 1 | 11 | 1 | 0.012 |
| 0.012 | 6 | 1 | 3 | 2 | 0.012 |
| 0.012 | 0.012 | 0.012 | 0.012 | 0.012 | 0.012 |
| 0.012 | 0.012 | 0.012 | 0.012 | 0.012 | 0.012 |
| 0.012 | 0.012 | 0.012 | 0.012 | 6 | 0.012 |
| 0.012 | 0.012 | 0.012 | 0.012 | 1 | 0.012 |
| 0.012 | 0.012 | 0.012 | 0.012 | 0.012 | 0.012 |
| 0.012 | 1 | 0.012 | 1 | 0.012 | 0.012 |
| 0.012 | 0.012 | 0.012 | 0.012 | 0.012 | 0.012 |
| 0.012 | 0.012 | 0.012 | 0.012 | 0.012 | 0.012 |
| 0.012 | 0.012 | 0.012 | 1 | 0.012 | 0.012 |
| 0.012 | 0.012 | 0.012 | 0.012 | 0.012 | 0.012 |
| 0.012 | 1 | 0.012 | 1 | 0.012 | 0.012 |
| 0.012 | 0.5 | 0.012 | 0.012 | 0.012 | 0.012 |
| 0.012 | 0.012 | 0.012 | 1 | 0.012 | 0.012 |
| 0.012 | 0.012 | 0.012 | 1 | 0.012 | 0.012 |
| 0.012 | 0.012 | 0.012 | 1 | 0.012 | 0.012 |
| 0.012 | 0.012 | 0.012 | 0.012 | 0.012 | 0.012 |
| 0.012 | 0.012 | 0.012 | 0.012 | 1 | 0.012 |
| 0.012 | 0.012 | 0.012 | 0.012 | 0.012 | 0.012 |
| 0.012 | 1 | 0.012 | 0.012 | 0.012 | 0.012 |
| 0.012 | 0.012 | 0.012 | 0.012 | 0.012 | 0.012 |
| 0.012 | 0.012 | 0.012 | 0.012 | 0.012 | 0.012 |
| 0.012 | 0.012 | 0.012 | 0.012 | 0.012 | 0.012 |
| 0.012 | 0.012 | 0.012 | 0.012 | 1 | 0.012 |
| 0.012 | 0.012 | 0.012 | 0.012 | 0.012 | 0.012 |
| 0.012 | 0.012 | 0.012 | 0.012 | 0.012 | 0.012 |
| 0.012 | 0.012 | 0.012 | 0.012 | 0.012 | 0.012 |
| 0.012 | 0.012 | 0.012 | 0.012 | 1 | 0.012 |
| 0.012 | 0.012 | 0.012 | 0.012 | 0.012 | 0.012 |
| 0.012 | 0.012 | 0.012 | 0.012 | 0.012 | 0.012 |
| 0.012 | 0.012 | 0.012 | 0.012 | 0.012 | 0.012 |
| 0.012 | 0.012 | 0.012 | 0.012 | 0.012 | 0.012 |
| 0.012 | 0.012 | 0.012 | 0.012 | 0.012 | 0.012 |
| 0.012 | 0.012 | 0.012 | 0.012 | 0.012 | 0.012 |
| 0.012 | 0.012 | 0.012 | 0.012 | 0.012 | 0.012 |
| 0.012 | 0.012 | 0.012 | 0.012 | 0.012 | 0.012 |
| 0.012 | 0.012 | 0.012 | 0.012 | 0.012 | 0.012 |
| 0.012 | 0.012 | 0.012 | 0.012 | 0.012 | 0.012 |
| 0.012 | 0.012 | 0.012 | 0.012 | 0.012 | 0.012 |
| 0.012 | 0.012 | 0.012 | 0.012 | 0.012 | 0.012 |
| 0.012 | 0.012 | 0.012 | 0.012 | 0.012 | 0.012 |
| 0.012 | 0.012 | 0.012 | 0.012 | 0.012 | 0.012 |
| 0.012 | 0.012 | 0.012 | 0.012 | 0.012 | 0.012 |
| 0.012 | 0.012 | 0.012 | 0.012 | 0.012 | 0.012 |
| 0.012 | 0.012 | 0.012 | 0.012 | 0.012 | 0.012 |
| 0.012 | 0.012 | 0.012 | 0.012 | 0.012 | 0.012 |
| 0.012 | 0.012 | 0.012 | 0.012 | 0.012 | 0.012 |
| 0.012 | 0.012 | 0.012 | 0.012 | 0.012 | 0.012 |
| 0.012 | 0.012 | 0.012 | 0.012 | 0.012 | 0.012 |

| 0.012 | 0.012 | 0.012 | 0.012 | 0.012 | 0.012 |
| --- | --- | --- | --- | --- | --- |
| 0.012 | 0.012 | 0.012 | 0.012 | 0.012 | 0.012 |
| 0.012 | 0.012 | 0.012 | 0.012 | 0.012 | 0.012 |
| 0.012 | 0.012 | 0.012 | 0.012 | 0.012 | 0.012 |
| 0.012 | 0.012 | 0.012 | 0.012 | 0.012 | 0.012 |
| 0.012 | 0.012 | 0.012 | 0.012 | 0.012 | 0.012 |
| 0.012 | 0.012 | 0.012 | 0.012 | 0.012 | 0.012 |
| 0.012 | 0.012 | 0.012 | 0.012 | 0.012 | 0.012 |
| 0.012 | 0.5 | 0.012 | 0.012 | 0.012 | 0.012 |
| 0.012 | 0.012 | 0.012 | 0.012 | 0.012 | 0.012 |
| 0.012 | 0.012 | 0.012 | 0.012 | 0.012 | 0.012 |
| 0.012 | 1 | 0.012 | 0.012 | 0.012 | 0.012 |
| 0.012 | 0.012 | 0.012 | 0.012 | 0.012 | 0.012 |
| 0.012 | 0.012 | 0.012 | 0.012 | 0.012 | 0.012 |
| 0.012 | 0.012 | 0.012 | 0.012 | 0.012 | 0.012 |
| 0.012 | 0.012 | 0.012 | 0.012 | 0.012 | 0.012 |
| 0.012 | 0.012 | 0.012 | 0.012 | 0.012 | 0.012 |
| 0.012 | 0.012 | 0.012 | 0.012 | 0.012 | 0.012 |

| Control_9 | Control_10 | Control_11 | Control_12 | Control_13 | Control_14 |
| --- | --- | --- | --- | --- | --- |
| 7470315.97 | 8104970.92 | 5833533.61 | 8881665.07 | 7732356.93 | 8027220.18 |
| 3611400.87 | 3935310.36 | 2767717.53 | 4272762.28 | 3724877.2 | 3827831.06 |
| 1724438.38 | 1873383.94 | 1270444.54 | 2060132.33 | 1800729.89 | 1811931.13 |
| 1554824.4 | 1656949.49 | 1116477.37 | 1749079.92 | 1533275.23 | 1565167.33 |
| 1106079.65 | 1189129.65 | 841772.52 | 1356286.04 | 1128802.46 | 1196447.58 |
| 1090953.05 | 1188013.11 | 844324.3 | 1270361.34 | 1098061.41 | 1178979.48 |
| 904203.52 | 814191.54 | 662067.25 | 862821.4 | 1123453.69 | 836409.35 |
| 723054.45 | 787552.3 | 569855.32 | 840362.31 | 774862.22 | 777928.26 |
| 585117.7 | 575273.84 | 429771.65 | 589479.1 | 608724.79 | 564813.65 |
| 532693.17 | 593304.13 | 445217.35 | 624219.67 | 574081.08 | 595863.5 |
| 496208.49 | 538437.39 | 380180.93 | 569353.4 | 503351.75 | 529341.08 |
| 469846.31 | 514892.36 | 404203.84 | 534216.6 | 539708.25 | 517376.73 |
| 468111.71 | 526747.99 | 383972.98 | 549812.76 | 488918.67 | 510007.8 |
| 431177.4 | 480540.19 | 356285.77 | 530272.77 | 463138.74 | 474762.74 |
| 436746.6 | 489739.54 | 312388.48 | 581554.76 | 405592.96 | 480517.83 |
| 429947.42 | 413237.38 | 358578.71 | 483130.94 | 481131.09 | 435885.61 |
| 405215.01 | 434499.36 | 319673.82 | 496772.12 | 431684.2 | 437451.53 |
| 417141.72 | 452104.78 | 325568.34 | 489986.53 | 436705.27 | 455098.27 |
| 375953.18 | 405226.07 | 303797.88 | 451359.06 | 391140.34 | 407393.11 |
| 386505.23 | 422235.9 | 328749.72 | 411248.48 | 429651.48 | 388568.58 |
| 377243.07 | 409232.8 | 314916.36 | 434862.08 | 383928.18 | 409204.09 |
| 348237.88 | 393682.49 | 282248.93 | 456984.54 | 339573.52 | 405563.49 |
| 354045.42 | 380353.2 | 275725.7 | 419152.58 | 363784.74 | 387561.13 |
| 333868.44 | 368388.31 | 186779.16 | 352786.68 | 284863.81 | 313634.1 |
| 336692.84 | 369759.63 | 259845 | 421050.4 | 325023.94 | 370236.79 |
| 338646.26 | 359692.79 | 247404.92 | 377832.59 | 333750.39 | 342621.65 |
| 313142.11 | 332486.5 | 213617.36 | 346731.78 | 308680.04 | 316794.21 |
| 295834.87 | 319476.27 | 228819.09 | 342195.47 | 303642.68 | 319847.79 |
| 293687.94 | 308390.39 | 229290.72 | 348280.5 | 326676.59 | 317127.18 |
| 254114.43 | 298591.49 | 209283.86 | 358073.78 | 257448.74 | 300142.79 |
| 256071.99 | 263324.41 | 191972.13 | 333302.58 | 284077.85 | 269569.8 |
| 267150.66 | 283771.96 | 202473.23 | 300064.71 | 275470.35 | 278881.78 |
| 252399.81 | 279211.45 | 193439.27 | 324809.45 | 252648.2 | 276081.87 |
| 267197.66 | 302941.21 | 231556.64 | 291363.68 | 295349.6 | 290145.67 |
| 264294.63 | 261835.32 | 168904.93 | 297803.82 | 240899.15 | 251611.8 |
| 248684.07 | 267005.64 | 177577.48 | 292940.62 | 240765.76 | 268213.86 |
| 231136.06 | 260721.15 | 191280.92 | 280469.38 | 252399.63 | 254334.99 |
| 204179.83 | 228323.8 | 173236.6 | 284131.5 | 230536.48 | 247629 |
| 237368.33 | 257552.37 | 181717.84 | 272049.81 | 246067.03 | 252811.85 |
| 234065.04 | 262573.67 | 181144.71 | 269471.84 | 240329.3 | 245833.55 |
| 228879.32 | 258623.92 | 193308.27 | 284781.37 | 235988.09 | 263868.81 |
| 219567.13 | 221554.35 | 178120.31 | 264677.17 | 242099.08 | 233875.75 |
| 239085.37 | 258444.89 | 180934.87 | 273779.52 | 242664.4 | 248358.86 |
| 239703.15 | 267096.69 | 202600.73 | 255685.57 | 265734.08 | 263850.63 |
| 223017.06 | 235280.27 | 171308.32 | 260570.97 | 214980.08 | 236739.19 |
| 210463.67 | 226923.07 | 159016.56 | 251400.97 | 210887.74 | 221590.17 |
| 213544.66 | 219113.43 | 181930.74 | 259547.11 | 242174.06 | 230527.02 |
| 221951.61 | 241451.82 | 169598.67 | 251009.51 | 220029.69 | 234518.62 |
| 217236.97 | 235471.65 | 165994.48 | 255106.04 | 216214.66 | 234751.97 |
| 204713.97 | 207704.07 | 156155.58 | 250467.55 | 242293.99 | 220388.37 |
| 197646.58 | 218725.52 | 160858.02 | 257415.58 | 198929.56 | 226602.58 |
| 182483.13 | 193925.19 | 166153.53 | 237401.58 | 203818.54 | 210083.62 |
| 180171.5 | 180094.43 | 147771.4 | 225559.04 | 198931.43 | 207851.75 |
| 174909 | 196727.13 | 148361.12 | 219253.31 | 195934.64 | 200964.51 |
| 177259.98 | 198093.4 | 132131.22 | 202117.38 | 176269.9 | 189385.74 |
| 168302.28 | 186880.17 | 134999.92 | 131316.09 | 214395.42 | 152732.37 |
| 152951.8 | 181816.09 | 123173.84 | 190808.35 | 167276.48 | 167229.27 |

| 170291.09 | 161288.96 | 107077.9 | 172693.12 | 174826.13 | 149861.82 |
| --- | --- | --- | --- | --- | --- |
| 165131.33 | 178500.08 | 125153.83 | 189881.7 | 165591.43 | 172685.82 |
| 150424.67 | 167159.51 | 127894.61 | 175774.96 | 163821.95 | 169095.25 |
| 136992.13 | 127146.47 | 103838.55 | 168251.91 | 148043.05 | 143402.36 |
| 143269.73 | 152643.45 | 102885.24 | 178312.14 | 128141.25 | 149148.73 |
| 138248.62 | 148151.4 | 107056.05 | 154519.03 | 167489.67 | 149525.83 |
| 115822.56 | 122304.08 | 96597.87 | 158732.56 | 150583.86 | 130243.58 |
| 118061.06 | 134677.37 | 96227.23 | 157741.97 | 127020.98 | 138286.85 |
| 118977.34 | 129207.03 | 96476.93 | 132805.92 | 122401.09 | 130337.05 |
| 117866.79 | 124799.6 | 87688.26 | 153045.44 | 97798.56 | 128313.18 |
| 106917.4 | 106933.64 | 87926.76 | 135825.89 | 123021.08 | 117701.57 |
| 107903.4 | 119790.93 | 80465.19 | 143031.97 | 75089.78 | 112605.23 |
| 106909.37 | 112503.08 | 95721.26 | 119104.64 | 132232.58 | 112953.79 |
| 110180.61 | 117449.72 | 86441.44 | 115546.72 | 110913.08 | 111430.24 |
| 93684.36 | 92888.5 | 91747.36 | 124804.52 | 124835.72 | 111097.45 |
| 96046.49 | 107134.84 | 83320.01 | 134418.56 | 105027.4 | 120466.78 |
| 104984.06 | 94684.04 | 85099 | 115204.23 | 139713.31 | 109922.67 |
| 112915.48 | 107661.41 | 73524.33 | 116933.8 | 118270 | 100029.77 |
| 88838.82 | 115394.71 | 78910.81 | 116872.01 | 112158.11 | 101730.8 |
| 92398.74 | 106793.41 | 74014.85 | 115315.75 | 104477.64 | 105654.53 |
| 97350.95 | 108978.25 | 81043.56 | 118737.08 | 101047.83 | 111029.99 |
| 94643.68 | 97580.7 | 77550.94 | 114111.1 | 107610.18 | 102021.06 |
| 98513 | 99818.73 | 78275.83 | 96335.11 | 112250.94 | 95549.96 |
| 95646.21 | 86163.72 | 60282.36 | 88720.98 | 124953.83 | 84589.68 |
| 76682.49 | 90317.26 | 53741.08 | 84319.76 | 70434.73 | 77862.61 |
| 68397.92 | 104234.11 | 88510.23 | 108861.37 | 95100.18 | 120738.15 |
| 78201.31 | 85799.18 | 61124.05 | 95299.5 | 84481.17 | 85079.99 |
| 81911.37 | 83803.76 | 66179.62 | 76488.54 | 93036.05 | 78801.78 |
| 77320.84 | 81200.63 | 65579.94 | 99118.43 | 78994.87 | 88326.95 |
| 77577.4 | 83264 | 60220.62 | 87144.49 | 82046.5 | 80982.07 |
| 69888.96 | 81320.99 | 57511.89 | 87298.45 | 81542.35 | 78482.65 |
| 73895.9 | 80662.58 | 54726.84 | 83526.51 | 70696.55 | 77791.36 |
| 70515.01 | 76847.19 | 50836.77 | 78368.74 | 68104.94 | 73147.64 |
| 58558.86 | 58034.3 | 39871.41 | 73424.89 | 54596.42 | 60501.59 |
| 66164.37 | 71207.86 | 53559.15 | 83897.41 | 69827.35 | 73467.97 |
| 60455.23 | 65920.91 | 46770.92 | 74394.48 | 60730.07 | 63269.93 |
| 66868.23 | 70187.88 | 47272.87 | 84064.23 | 52783.81 | 71564.45 |
| 59086.99 | 73031.1 | 53742.63 | 76861.31 | 73395.61 | 70906.63 |
| 50249.79 | 54727.96 | 44848.4 | 78514.08 | 50008.04 | 60844.4 |
| 75457.69 | 77103.77 | 43876.54 | 72858.11 | 65314.72 | 65060.34 |
| 63962.14 | 65474.34 | 44421.44 | 74415.6 | 58895.81 | 66293.18 |
| 52099.65 | 55859.54 | 41623.72 | 68472.74 | 47777.53 | 58247.23 |
| 58570.07 | 63753.69 | 42724.35 | 76443.69 | 53090.74 | 62848.26 |
| 53730.69 | 57215.92 | 39351.9 | 66152.73 | 50283.28 | 56524.3 |
| 57483.22 | 57636.37 | 46554.42 | 69956.31 | 58004.15 | 63935.18 |
| 43570.33 | 70474.43 | 59505.74 | 71478.35 | 58147.09 | 77665.66 |
| 43828.59 | 43834.69 | 30041.17 | 63760.99 | 44237.19 | 45351.31 |
| 53896.53 | 58059.83 | 43702.4 | 53801.2 | 59691.63 | 57264.61 |
| 49750.82 | 57234.14 | 39451.68 | 64730.46 | 48913.66 | 57303.35 |
| 50898.38 | 52402.09 | 36074.61 | 58673.07 | 51036.76 | 51424.73 |
| 53082.82 | 57250.57 | 44166.25 | 61072.1 | 53542.47 | 58697.77 |
| 48117.44 | 50771.86 | 36985.11 | 55604.66 | 50724.61 | 55348.21 |
| 40567.09 | 58578.68 | 54591.67 | 66390.02 | 59216.67 | 71505.41 |
| 44861.02 | 57732.04 | 40307.92 | 57793.36 | 48547.66 | 53993.17 |
| 51466 | 55955.36 | 33738.5 | 55097.17 | 52470.51 | 50038.56 |
| 39904.02 | 49505.97 | 32341.34 | 60593.79 | 40661.66 | 46924 |
| 46121.64 | 54899.16 | 32031.8 | 55479.01 | 47724.23 | 46586.68 |
| 37876.32 | 45497.3 | 32154.17 | 52431.49 | 35918.13 | 47584.55 |

| 32022.97 | 36085.02 | 34911.52 | 52516.04 | 38492.4 | 43661.35 |
| --- | --- | --- | --- | --- | --- |
| 40029.35 | 52568.49 | 32092.51 | 49952.81 | 42693.16 | 44246.5 |
| 47850.76 | 39282.74 | 32623.63 | 42798.66 | 75105.08 | 43895.18 |
| 34043.7 | 36540.2 | 24196.89 | 42447.24 | 43239.37 | 37587.13 |
| 40010.6 | 41252.72 | 27411.7 | 44603.18 | 36834.11 | 40695.3 |
| 36293.05 | 45527.43 | 20955.67 | 35955.38 | 29092.01 | 37601.89 |
| 34986.32 | 39702.7 | 22224.27 | 44601.1 | 25530 | 39156.33 |
| 56630.88 | 35912.62 | 19699.64 | 50928.7 | 42581.91 | 39415.67 |
| 34662.01 | 31269.26 | 25170.68 | 44081.19 | 39447.4 | 37332.01 |
| 29098.21 | 31635.5 | 25092.33 | 43245.46 | 27931.2 | 35440.71 |
| 34405.56 | 28271.05 | 28426.1 | 42559.32 | 35977.57 | 34125.18 |
| 39438.3 | 41425.35 | 20054.09 | 38390.49 | 32061.72 | 39500.95 |
| 35670.33 | 37098.36 | 23656.32 | 38728.11 | 34471.57 | 35636.77 |
| 31756.34 | 31279.16 | 19832.05 | 25467.58 | 26021.49 | 24841.68 |
| 33475.34 | 35830.09 | 24500.02 | 41829.68 | 28555.74 | 35811.1 |
| 29614.01 | 32516 | 18903.6 | 33693.35 | 33482.49 | 29493.59 |
| 32666.77 | 36205.92 | 24901.26 | 37005.05 | 33026.7 | 34000.13 |
| 26717.54 | 26623.98 | 18317.74 | 43440.78 | 23620.7 | 30344.88 |
| 21175.49 | 20140.33 | 21119.9 | 36687.04 | 20172.97 | 23151.28 |
| 31422.9 | 35956.8 | 24406.93 | 35994.26 | 32733.37 | 33169.13 |
| 21062 | 31288.21 | 31877.66 | 36274.79 | 26429.91 | 39608.5 |
| 30224.03 | 30663.68 | 20149.19 | 34310.61 | 34920.06 | 29062.6 |
| 25499.43 | 29860.54 | 22709.64 | 36926.47 | 30146.81 | 30448.85 |
| 54985.25 | 31846.85 | 10799.25 | 52740.35 | 20703 | 38193.75 |
| 18709.75 | 27752.92 | 32699.84 | 35173.92 | 24716.41 | 40583.75 |
| 25550.17 | 29618.26 | 22495.84 | 32251.89 | 25444.2 | 30737.88 |
| 29208.95 | 28550.07 | 18745.01 | 29566.87 | 31309.2 | 27038.13 |
| 22563.39 | 27613.63 | 18088.79 | 30378.6 | 28308.03 | 27348.46 |
| 25135.5 | 27078.72 | 18388.01 | 30605.51 | 27777.2 | 26057.38 |
| 25476.42 | 27261.3 | 20994.03 | 33412.43 | 24399.2 | 27478.05 |
| 21965.4 | 25385.75 | 19257.24 | 32680.48 | 20126.84 | 29259.75 |
| 21688 | 19903.08 | 11170 | 20425.08 | 13067 | 20666 |
| 26366 | 27156.02 | 15830.68 | 26892.78 | 28155.06 | 23591.1 |
| 24131.54 | 25628.62 | 17039.74 | 30392.42 | 22660.7 | 25994.88 |
| 26228.83 | 26281.94 | 19399.46 | 29673.11 | 26753.83 | 26958.93 |
| 20620.97 | 22705.01 | 26524.76 | 31358.4 | 27619.03 | 29575.8 |
| 20075.25 | 18620.23 | 15652.73 | 26537.03 | 23970.53 | 20761.13 |
| 22004.34 | 23135.38 | 16705.84 | 24921.18 | 21650.7 | 23013.88 |
| 20749.61 | 24080.63 | 28805.85 | 27364.91 | 26023.06 | 28846.6 |
| 18899.34 | 20808.99 | 11189.09 | 22469.87 | 18992.54 | 17926.21 |
| 15147.33 | 17903.49 | 11702.33 | 19951.62 | 21406.87 | 17124.05 |
| 18845.31 | 16001.05 | 18779.1 | 21823.49 | 25585.4 | 22540.43 |
| 18581.47 | 22037.53 | 24021.31 | 30461.42 | 19116.53 | 27328.3 |
| 20957.87 | 22542.55 | 15742.09 | 24035.01 | 20759.87 | 22120.63 |
| 20520.17 | 22402.26 | 15770.84 | 23746.89 | 20599.7 | 22098.38 |
| 20705.17 | 22420.05 | 15735.84 | 23740.51 | 20689.87 | 22045.38 |
| 20700.17 | 22418.05 | 15734.84 | 23737.51 | 20666.87 | 22042.38 |
| 16111.78 | 9156.91 | 12636.54 | 20801.9 | 14634.52 | 15228.73 |
| 22029.17 | 24026.88 | 13734.17 | 25094.1 | 17443.19 | 22206.13 |
| 12407 | 13817.37 | 10663.16 | 17529 | 18355.37 | 15427.55 |
| 11817.33 | 18346.25 | 20943.83 | 21897 | 14885.08 | 26488 |
| 11817.33 | 18346.25 | 20943.83 | 21897 | 14885.08 | 26488 |
| 18565.17 | 18541.13 | 13483.51 | 22023.09 | 16874.37 | 19239.3 |
| 12495.75 | 10734.87 | 11374.8 | 20221.64 | 13179.07 | 12172.26 |
| 15409.38 | 16831.09 | 13350.34 | 19951.3 | 14642.61 | 16301.22 |
| 15830.64 | 17283.58 | 13518.26 | 16564.71 | 20164.2 | 18059.63 |
| 17187.96 | 17097.3 | 15606.2 | 16894.18 | 21345.37 | 18110.46 |
| 15827.63 | 18703.13 | 10353.94 | 21354.04 | 13984.66 | 16377.33 |

| 16565.4 | 18751.82 | 14375.58 | 22043.41 | 12466.49 | 21254.75 |
| --- | --- | --- | --- | --- | --- |
| 10611.5 | 13101.01 | 8885.96 | 12819.14 | 14134.37 | 12581.55 |
| 15243.7 | 17448.58 | 7568.41 | 14895.9 | 12757.84 | 15380.5 |
| 14974.53 | 17724.42 | 10846.75 | 15984 | 12785.67 | 16839.75 |
| 8365 | 15668.36 | 17121 | 20113.36 | 13514 | 22092 |
| 12138.33 | 18706 | 12572.8 | 13659.5 | 17201.18 | 13324 |
| 9045.97 | 8277.26 | 9870.69 | 16845.19 | 9013.24 | 9982.6 |
| 12221.49 | 9354.9 | 20799.16 | 7994.64 | 18156.11 | 14551.8 |
| 10144 | 14461.79 | 13463.5 | 17331.12 | 9964 | 17740 |
| 8830.28 | 3954.53 | 6848.24 | 12891.14 | 8698.48 | 8546.18 |
| 11863.36 | 12725.49 | 8807.42 | 14176.74 | 10577.33 | 12902.25 |
| 10457.87 | 10136.23 | 6682.49 | 7650.36 | 8810.12 | 8850.05 |
| 5055.3 | 3954.19 | 5781.93 | 14636.07 | 4425.54 | 7087.3 |
| 5052.3 | 3953.07 | 5779.93 | 14635.95 | 4425.54 | 7087.3 |
| 11699 | 14625.51 | 12023.56 | 13378.56 | 10648.03 | 12959.05 |
| 11441.3 | 14501.01 | 12016.31 | 13081.06 | 10555.03 | 12880.8 |
| 7370.87 | 8022.12 | 8908 | 16778.79 | 9573.5 | 10642.5 |
| 10440.66 | 12644.39 | 7074.18 | 7016.93 | 10810.36 | 7742.22 |
| 7245.8 | 7371.87 | 7637.18 | 12682.25 | 4902.04 | 6728.8 |
| 9373.83 | 10666.66 | 4020.42 | 10888.32 | 6925.34 | 7837.75 |
| 9118.03 | 8637.37 | 7767.25 | 11751.12 | 7502.83 | 11202.75 |
| 9118.03 | 8637.37 | 7767.25 | 11751.12 | 7502.83 | 11202.75 |
| 9118.03 | 8637.37 | 7767.25 | 11751.12 | 7502.83 | 11202.75 |
| 9118.03 | 8637.37 | 7767.25 | 11751.12 | 7502.83 | 11202.75 |
| 9118.03 | 8637.37 | 7767.25 | 11751.12 | 7502.83 | 11202.75 |
| 9118.03 | 8637.37 | 7767.25 | 11751.12 | 7502.83 | 11202.75 |
| 8015.75 | 8601.62 | 4539.83 | 9547.2 | 10607 | 6609.58 |
| 8015.75 | 8601.62 | 4539.83 | 9547.2 | 10607 | 6609.58 |
| 6776.47 | 8578.89 | 10344.26 | 11826.94 | 8751.7 | 9987.8 |
| 6490.48 | 6449.56 | 8377.82 | 8127.34 | 8738.4 | 7954.43 |
| 5839.5 | 7276.24 | 6939.75 | 12324.24 | 4969.5 | 8537 |
| 2628 | 1000.6 | 1281 | 13222.6 | 1278 | 4452 |
| 2628 | 1000.6 | 1281 | 13222.6 | 1278 | 4452 |
| 8263.64 | 3787.43 | 6859.51 | 4679.73 | 14086.87 | 6982.88 |
| 2586 | 995.48 | 1278 | 13048.48 | 960 | 4350 |
| 4412.67 | 5089.34 | 5479.26 | 6680.06 | 4600.7 | 5130.3 |
| 6156.87 | 5254.74 | 2161.58 | 5378.74 | 6940.5 | 5175.83 |
| 8113.17 | 8287.71 | 3824.19 | 6029.04 | 4328.67 | 6553.75 |
| 1677 | 670.44 | 853.2 | 8414.44 | 81 | 2812 |
| 3747.33 | 1708.15 | 3206.17 | 5372.98 | 9407.5 | 3629.5 |
| 2899.47 | 1376 | 3342.65 | 5064.47 | 5542.7 | 2847.8 |
| 4032.83 | 5151.5 | 1573.84 | 2542 | 3986.67 | 2138 |
| 2694 | 1004 | 1987 | 8481 | 4263 | 2965 |
| 3638.37 | 3178.24 | 1645.33 | 3455.49 | 6362 | 4189.08 |
| 3638.37 | 3177.24 | 1645.33 | 3455.49 | 6362 | 4189.08 |
| 2708.65 | 2430.2 | 3748.59 | 2180.1 | 5646.7 | 3623.46 |
| 1795.5 | 716.36 | 1777.2 | 4709.86 | 4221 | 2846 |
| 1991 | 3970.5 | 5807.5 | 5431.5 | 4090 | 5575.5 |
| 2794.33 | 4127.12 | 1088.92 | 2375.62 | 3149 | 1858.5 |
| 913 | 359.36 | 435.75 | 4527.36 | 495.5 | 1712.5 |
| 1648.84 | 2435.62 | 866.11 | 2079.79 | 2388.33 | 2007.08 |
| 3065.53 | 4479.67 | 1233.42 | 2706.5 | 3329.5 | 2355.5 |
| 888 | 333.24 | 427.75 | 4510.24 | 447.5 | 1683 |
| 2659.67 | 3645.33 | 1745.2 | 2023 | 2864.33 | 2161 |
| 2243.83 | 3761.29 | 1008.62 | 2341.62 | 3091 | 1671 |
| 2242.83 | 3761.29 | 1008.62 | 2341.62 | 3089 | 1669 |
| 892 | 333.48 | 427.75 | 4512.48 | 445.5 | 1684 |
| 1646.67 | 809.41 | 409.83 | 1440.24 | 1402.5 | 1002.83 |

| 1138.5 | 464.12 | 474 | 4371.12 | 502 | 1768 |
| --- | --- | --- | --- | --- | --- |
| 2734.33 | 4071.67 | 1060.17 | 2266.5 | 3060.5 | 1637.5 |
| 781 | 318 | 419 | 4189 | 19 | 1369 |
| 276.5 | 156.48 | 22 | 220.98 | 189 | 96 |
| 2506.03 | 3591.12 | 591.5 | 2133.12 | 2308.83 | 1575 |
| 3346.4 | 816.58 | 163.5 | 2829.33 | 1424 | 1130 |
| 2783.27 | 1748.6 | 3817.73 | 1954.87 | 1376.07 | 1356.93 |
| 1610.17 | 777.29 | 400.83 | 1431.62 | 1352.5 | 985.33 |
| 44.5 | 46.48 | 13 | 14.48 | 56.5 | 29.5 |
| 1162 | 149.75 | 394.25 | 949.25 | 358.5 | 910.25 |
| 1162 | 149.75 | 394.25 | 949.25 | 358.5 | 910.25 |
| 1090 | 278 | 1151 | 523 | 4407 | 981.5 |
| 523 | 1018.67 | 591.2 | 540.5 | 689.5 | 616 |
| 23 | 23.36 | 3 | 6.36 | 31 | 38 |
| 837.46 | 599.8 | 3121.86 | 421.26 | 912.86 | 519.14 |
| 477 | 1266 | 402 | 718 | 346 | 19 |
| 766 | 388.67 | 798 | 334.67 | 1457.67 | 1235 |
| 29 | 30 | 4 | 4 | 47 | 17 |
| 18 | 12.24 | 6 | 13.24 | 32 | 34.5 |
| 13 | 9.36 | 1 | 4.36 | 11 | 34 |
| 37.5 | 33.36 | 7 | 11.86 | 49.75 | 35.5 |
| 498 | 277 | 24.5 | 68.5 | 65.5 | 211.5 |
| 14.4 | 7.24 | 1 | 10.24 | 10 | 11 |
| 221 | 469 | 433.67 | 124 | 708.67 | 58 |
| 49 | 8.24 | 5 | 195.24 | 318 | 117 |
| 42 | 5.36 | 3 | 174.36 | 318 | 102 |
| 42 | 5.24 | 3 | 174.24 | 318 | 102 |
| 42 | 5.24 | 3 | 174.24 | 318 | 102 |
| 42 | 5.24 | 3 | 174.24 | 318 | 102 |
| 42 | 5.36 | 3 | 174.36 | 318 | 102 |
| 42 | 5.24 | 3 | 174.24 | 318 | 102 |
| 42 | 5.24 | 3 | 174.24 | 318 | 102 |
| 42 | 5.24 | 3 | 174.24 | 318 | 102 |
| 42 | 5.24 | 3 | 174.24 | 318 | 102 |
| 42 | 5.24 | 3 | 174.24 | 318 | 102 |
| 42 | 5.24 | 3 | 174.24 | 318 | 102 |
| 42 | 5.24 | 3 | 174.24 | 318 | 102 |
| 42 | 5.24 | 3 | 174.24 | 318 | 102 |
| 42 | 5.24 | 3 | 174.24 | 318 | 102 |
| 1 | 1 | 0.012 | 0.012 | 2 | 0.012 |
| 155 | 66 | 25 | 73 | 227 | 170 |
| 155 | 66 | 25 | 73 | 227 | 170 |
| 86 | 50 | 41 | 138 | 53 | 106 |
| 51 | 10.12 | 7 | 67.12 | 38 | 53 |
| 24 | 56 | 41 | 74 | 4 | 31 |
| 24 | 56 | 41 | 74 | 4 | 31 |
| 5 | 2 | 1 | 3 | 23 | 3 |
| 5 | 2 | 1 | 3 | 23 | 3 |
| 5 | 2 | 1 | 3 | 23 | 3 |
| 5 | 2 | 1 | 3 | 23 | 3 |
| 5 | 2 | 1 | 3 | 23 | 3 |
| 5 | 2 | 1 | 3 | 23 | 3 |
| 5 | 2 | 1 | 3 | 23 | 3 |
| 24 | 9.67 | 13 | 5 | 6 | 3 |
| 3 | 2.12 | 2 | 0.12 | 0.012 | 0.012 |
| 3 | 2.12 | 2 | 0.12 | 0.012 | 0.012 |
| 3 | 2.12 | 2 | 0.12 | 0.012 | 0.012 |
| 3 | 2.12 | 2 | 0.12 | 0.012 | 0.012 |

| 3 | 2.12 | 2 | 0.12 | 0.012 | 0.012 |
| --- | --- | --- | --- | --- | --- |
| 9 | 15 | 2 | 4 | 22 | 0.012 |
| 0.012 | 0.012 | 0.012 | 0.012 | 0.012 | 0.012 |
| 1 | 0.24 | 0.012 | 0.24 | 2 | 2 |
| 4 | 0.012 | 0.012 | 1 | 0.012 | 0.012 |
| 0.012 | 0.012 | 0.012 | 0.012 | 0.012 | 0.012 |
| 0.012 | 1 | 0.012 | 0.012 | 0.012 | 0.012 |
| 0.012 | 0.012 | 0.012 | 0.012 | 0.012 | 0.012 |
| 8 | 4 | 0.012 | 0.012 | 0.012 | 1 |
| 0.012 | 0.012 | 0.012 | 0.012 | 0.012 | 1 |
| 0.012 | 0.012 | 0.012 | 0.012 | 0.012 | 0.012 |
| 0.012 | 0.012 | 0.012 | 0.012 | 0.012 | 0.012 |
| 8 | 0.012 | 0.012 | 0.012 | 0.012 | 0.012 |
| 0.012 | 0.012 | 0.012 | 0.012 | 0.012 | 0.012 |
| 0.012 | 0.012 | 0.012 | 0.012 | 0.012 | 0.012 |
| 0.012 | 1 | 0.012 | 0.012 | 0.012 | 0.012 |
| 0.012 | 0.012 | 0.012 | 0.012 | 0.012 | 0.012 |
| 1 | 0.012 | 0.012 | 0.012 | 0.012 | 0.012 |
| 0.012 | 0.012 | 0.012 | 0.012 | 0.012 | 0.012 |
| 0.012 | 0.012 | 0.012 | 0.012 | 0.012 | 0.012 |
| 0.2 | 0.012 | 0.012 | 0.012 | 0.012 | 0.012 |
| 1 | 0.012 | 0.012 | 0.012 | 0.012 | 0.012 |
| 0.012 | 0.012 | 0.012 | 0.012 | 0.012 | 0.012 |
| 0.012 | 0.012 | 0.012 | 0.012 | 0.012 | 0.012 |
| 0.012 | 0.012 | 0.012 | 0.012 | 0.012 | 0.012 |
| 0.012 | 2 | 0.012 | 0.012 | 0.012 | 0.012 |
| 0.012 | 0.012 | 0.012 | 0.012 | 0.012 | 0.012 |
| 0.012 | 0.012 | 0.012 | 0.012 | 0.012 | 0.012 |
| 0.012 | 0.012 | 0.012 | 0.012 | 0.012 | 0.012 |
| 0.012 | 1 | 0.012 | 1 | 0.012 | 0.012 |
| 0.012 | 0.012 | 0.012 | 0.012 | 0.012 | 0.012 |
| 0.012 | 0.012 | 0.012 | 0.012 | 0.012 | 0.012 |
| 0.012 | 0.012 | 0.012 | 0.012 | 0.012 | 1 |
| 0.012 | 0.012 | 0.012 | 0.012 | 0.012 | 0.012 |
| 0.012 | 0.012 | 0.012 | 0.012 | 0.012 | 0.012 |
| 0.012 | 0.012 | 0.012 | 0.012 | 0.012 | 0.012 |
| 0.012 | 0.12 | 0.012 | 0.12 | 0.012 | 0.012 |
| 0.012 | 0.012 | 0.012 | 0.012 | 0.012 | 0.012 |
| 0.012 | 0.012 | 0.012 | 0.012 | 0.012 | 0.012 |
| 0.012 | 0.012 | 0.012 | 0.012 | 0.012 | 0.012 |
| 0.012 | 0.012 | 0.012 | 0.012 | 0.012 | 0.012 |
| 0.012 | 0.012 | 0.012 | 0.012 | 0.012 | 0.012 |
| 0.012 | 0.012 | 0.012 | 0.012 | 0.012 | 0.012 |
| 0.012 | 0.012 | 0.012 | 0.012 | 0.012 | 0.012 |
| 0.012 | 0.012 | 0.012 | 0.012 | 0.012 | 0.012 |
| 0.012 | 0.012 | 0.012 | 0.012 | 0.012 | 0.012 |
| 0.012 | 0.012 | 0.012 | 0.012 | 0.012 | 0.012 |
| 0.012 | 0.012 | 0.012 | 0.012 | 0.012 | 0.012 |
| 0.012 | 0.012 | 0.012 | 0.012 | 0.012 | 0.012 |
| 0.012 | 0.012 | 0.012 | 0.012 | 0.012 | 0.012 |
| 0.012 | 0.012 | 0.012 | 0.012 | 0.012 | 0.012 |
| 0.012 | 0.012 | 0.012 | 0.012 | 0.012 | 0.012 |
| 0.012 | 0.012 | 0.012 | 0.012 | 0.012 | 0.012 |
| 0.012 | 0.012 | 0.012 | 0.012 | 0.012 | 0.012 |
| 0.012 | 0.012 | 0.012 | 0.012 | 0.012 | 0.012 |
| 0.012 | 0.012 | 0.012 | 0.012 | 0.012 | 0.012 |
| 0.012 | 0.012 | 0.012 | 0.012 | 0.012 | 0.012 |
| 0.012 | 0.012 | 0.012 | 0.012 | 0.012 | 0.012 |

| 0.012 | 0.012 | 0.012 | 0.012 | 0.012 | 0.012 |
| --- | --- | --- | --- | --- | --- |
| 0.012 | 0.012 | 0.012 | 0.012 | 0.012 | 0.012 |
| 0.012 | 0.012 | 0.012 | 0.012 | 0.012 | 0.012 |
| 0.012 | 0.012 | 0.012 | 0.012 | 0.012 | 0.012 |
| 0.012 | 0.012 | 0.012 | 0.012 | 0.012 | 0.012 |
| 0.012 | 0.012 | 0.012 | 0.012 | 0.012 | 0.012 |
| 0.012 | 0.012 | 0.012 | 0.012 | 0.012 | 0.012 |
| 0.012 | 0.012 | 0.012 | 0.012 | 0.012 | 0.012 |
| 0.012 | 0.012 | 0.012 | 0.012 | 0.012 | 0.012 |
| 0.012 | 0.012 | 0.012 | 0.012 | 0.012 | 0.012 |
| 0.012 | 0.012 | 0.012 | 0.012 | 0.012 | 0.012 |
| 0.2 | 0.012 | 0.012 | 0.012 | 0.012 | 0.012 |
| 0.012 | 0.012 | 0.012 | 0.012 | 0.012 | 0.012 |
| 0.012 | 0.012 | 0.012 | 0.012 | 0.012 | 0.012 |
| 0.012 | 0.012 | 0.012 | 0.012 | 0.012 | 0.012 |
| 0.012 | 0.012 | 0.012 | 0.012 | 0.012 | 0.012 |
| 0.012 | 0.012 | 0.012 | 0.012 | 0.012 | 0.012 |
| 0.012 | 0.012 | 0.012 | 0.012 | 0.012 | 0.012 |

| Control_15 | Control_16 | Control_17 | Control_18 | Control_19 | Control_20 |
| --- | --- | --- | --- | --- | --- |
| 9227861.75 | 8683224.01 | 9099320.08 | 8077856.19 | 7476269.53 | 8880062 |
| 4328129.81 | 4209020.68 | 4308778.09 | 3896901.76 | 3528516.46 | 4263745.9 |
| 2078207.79 | 2029039.66 | 2126987.37 | 1942067.91 | 1639240.25 | 2133635.95 |
| 1612719.06 | 1785432.71 | 1766275.69 | 1647114.62 | 1409455.08 | 1787503.46 |
| 1399372.29 | 1256853.75 | 1373154.75 | 1226127.48 | 1073950.47 | 1366886.34 |
| 1317319.37 | 1229269.93 | 1259718.4 | 1091951.28 | 1082094 | 1224277.94 |
| 856267.39 | 898750.36 | 1015137.91 | 1016361.39 | 647905.71 | 999252.9 |
| 881074.03 | 829566.43 | 850719.3 | 759299.14 | 717959.47 | 829467.91 |
| 547134.48 | 627534.54 | 638658.36 | 585420.22 | 497588.55 | 623982.91 |
| 671981.45 | 621186.04 | 620317.02 | 526015.29 | 566220.53 | 581395.25 |
| 578556.42 | 564548.22 | 581956 | 495921.31 | 487742.35 | 551175.92 |
| 587190.66 | 556188.5 | 552068.68 | 459110.23 | 477022.73 | 499303.34 |
| 558299.89 | 561800.25 | 549061.81 | 462823.24 | 472823.35 | 515584.04 |
| 537806.36 | 525928.39 | 555124.45 | 481165.46 | 472128.09 | 525775.48 |
| 591351.56 | 522655.44 | 570523.95 | 510500.41 | 433664.81 | 592336.25 |
| 504499.08 | 445329.84 | 502978.81 | 540261.37 | 381272.75 | 547838.09 |
| 514888.94 | 461813.8 | 517276.44 | 482806.73 | 388693.92 | 522426.07 |
| 516173.25 | 471955.45 | 501181.01 | 420751.24 | 410909.21 | 466882.83 |
| 453919.28 | 425995.41 | 452689.6 | 392282.63 | 377807.36 | 435463.79 |
| 391314.1 | 460421.93 | 431966.63 | 363236.56 | 353833.34 | 399142.7 |
| 448711.39 | 427491.74 | 430507.09 | 387610.34 | 385446.07 | 422410.76 |
| 488102.66 | 403426.56 | 445576.4 | 381590.07 | 384187.28 | 436053.98 |
| 437807.9 | 394199.51 | 418979.99 | 362811.9 | 345070.41 | 405842.34 |
| 292862.42 | 450563.52 | 377226.92 | 309215.95 | 324596.14 | 356778.92 |
| 428618 | 380834.34 | 413202.85 | 357804.02 | 344459.46 | 413863.46 |
| 363230.47 | 388055.76 | 376186.56 | 350585.24 | 319702.02 | 376892.8 |
| 330159.29 | 351783.51 | 350559.66 | 323341.05 | 277670.86 | 355501.38 |
| 357722 | 330849.99 | 348977.62 | 303357.98 | 286187.18 | 332346.32 |
| 371507.74 | 327251.15 | 350278.75 | 295854.37 | 279580.12 | 330704.59 |
| 392033.12 | 313857.1 | 347926.17 | 309559.48 | 285374.17 | 351421.73 |
| 342442.36 | 271238.36 | 319256.14 | 362884.54 | 218619.74 | 397694.97 |
| 311097.8 | 293267.81 | 307322.61 | 273932.49 | 248295.38 | 294935 |
| 338359.88 | 281596.56 | 317823.88 | 299862.52 | 267616.96 | 325989.78 |
| 308487.92 | 316945.42 | 294456.58 | 232278.22 | 280884.28 | 255149.94 |
| 260947.14 | 284731.18 | 304968.06 | 312221.82 | 201782.71 | 333974.7 |
| 298298.36 | 268113.98 | 287086.2 | 244780.12 | 234872.7 | 282221.92 |
| 318691.04 | 279242.05 | 276340.68 | 264382.55 | 257002.09 | 279780.83 |
| 350062.98 | 252863.22 | 305147.65 | 241220.4 | 253097 | 270215.49 |
| 285918.48 | 270246.83 | 272474.4 | 242096.21 | 234517.44 | 265804.2 |
| 260061.79 | 280124.77 | 272535.71 | 251493.89 | 236666.53 | 274168.95 |
| 301424.51 | 260649.74 | 284376.66 | 232611.29 | 251017.62 | 264103.69 |
| 293519.82 | 238930.8 | 278317.24 | 267554.66 | 202781.67 | 281811.54 |
| 272531.07 | 276186.92 | 270063.39 | 255017.94 | 228639.51 | 275062.15 |
| 271288.16 | 284562.27 | 255912.3 | 215388.96 | 243682.32 | 235947.1 |
| 265107.78 | 251256 | 265671.79 | 224036.13 | 232356.05 | 252681 |
| 252167.78 | 240281.58 | 280991.64 | 275665.98 | 196032.63 | 286199.72 |
| 287836.2 | 235147.8 | 270057.68 | 269024.28 | 202819.68 | 278770.45 |
| 257291.36 | 253735.25 | 254578.2 | 208671.95 | 224944.08 | 235019.64 |
| 263625.67 | 241658.45 | 248661.77 | 212317.19 | 213417.83 | 241265.22 |
| 286811 | 233012.64 | 267435.43 | 238210.12 | 181569.93 | 264076.64 |
| 283765.78 | 220658.31 | 253086.24 | 214465.81 | 207316.65 | 249077.66 |
| 258167.66 | 202001.97 | 245624.16 | 247270.74 | 198624.22 | 256763.56 |
| 252863 | 179423.6 | 235103.24 | 189804.83 | 179465.82 | 219112.49 |
| 236551.92 | 200070.1 | 230271.1 | 195731.39 | 182310.61 | 211533.42 |
| 209126.71 | 209556.81 | 200668.98 | 161007.75 | 177708.63 | 184187.56 |
| 103445.7 | 177309.14 | 187754.42 | 198364.24 | 108702.88 | 176904.83 |
| 200713.98 | 200110.14 | 192660.93 | 166636.6 | 182020.5 | 181103.33 |

| 146022 | 190074.19 | 183053.37 | 196878.01 | 119017.17 | 199865.29 |
| --- | --- | --- | --- | --- | --- |
| 185906.01 | 185231.63 | 183790.4 | 160923.4 | 162180.98 | 179567.33 |
| 192898.02 | 177154.52 | 172592.29 | 143640.08 | 163252.32 | 159213.18 |
| 196371.37 | 130886.48 | 180878.66 | 189377.82 | 116504.14 | 196575.91 |
| 173093 | 154394.73 | 178717.99 | 174848.75 | 132062.77 | 191485.32 |
| 155473.48 | 156950.6 | 171509.56 | 140198.6 | 130430.98 | 149190.68 |
| 184988.57 | 126456.28 | 186375.85 | 178450.25 | 101753.97 | 189113.97 |
| 170004.94 | 147265.49 | 165171.52 | 164399.53 | 129883 | 175190.94 |
| 143526.12 | 135186.51 | 135764.97 | 108661.13 | 119301.24 | 120964.02 |
| 158028.95 | 127680.05 | 147263.54 | 123846.26 | 109110.78 | 151928.94 |
| 158926.25 | 109988.85 | 152279.78 | 149154.93 | 97155.03 | 154297.77 |
| 129892.34 | 116677.68 | 142925.34 | 135318.02 | 106613.05 | 151727.3 |
| 123746.99 | 124743.12 | 135345.94 | 127039.92 | 101429.13 | 128625.63 |
| 110948.9 | 121321.27 | 117518.66 | 106206.1 | 99978.82 | 111722.49 |
| 154782.37 | 98972.02 | 135586.8 | 136077.69 | 93759.28 | 137371.97 |
| 162417.71 | 100675.79 | 132710.64 | 103176.6 | 110178.4 | 121029.8 |
| 138415.05 | 97325.58 | 122105.8 | 99203.43 | 82366.55 | 107434.43 |
| 100499.94 | 120599.56 | 115606.96 | 120707.76 | 84610 | 124414.51 |
| 140391.94 | 126992.26 | 116345.11 | 114150.64 | 113353.19 | 116389.06 |
| 123859.43 | 109270.55 | 115718.16 | 104338.32 | 94800.87 | 114781.06 |
| 129113.99 | 111499.34 | 117726.53 | 98535.77 | 103375.57 | 109938.85 |
| 121306.28 | 103432.48 | 114750.3 | 99009.63 | 91554.51 | 108650.31 |
| 97688.71 | 108309.16 | 102649.47 | 93299.64 | 81462.76 | 95321.51 |
| 89355.44 | 92701.6 | 97989.88 | 94654.3 | 60279.82 | 93799.42 |
| 80772.27 | 102767.93 | 97870.74 | 90117.49 | 77919.19 | 91357.91 |
| 161407.55 | 121198.18 | 104157.11 | 54377.4 | 129363.86 | 76225.45 |
| 104257.57 | 93665.11 | 101287.37 | 98636.59 | 73916.67 | 104717.26 |
| 76728.66 | 90826.73 | 84014.75 | 81522.82 | 67604.66 | 78343.28 |
| 111707.98 | 88467.95 | 98441.2 | 81476.87 | 81357.13 | 95635.47 |
| 86009.75 | 88796.22 | 90720.21 | 83315.44 | 72743.26 | 89345.95 |
| 102295.51 | 94565.54 | 92521.34 | 75468 | 75128.17 | 83229.04 |
| 81905.84 | 84358.56 | 79646.15 | 62905.56 | 73819.73 | 75307.58 |
| 77168.2 | 80832.37 | 77259.18 | 65919.27 | 67718.25 | 74749.98 |
| 76170.13 | 65017.31 | 87677.03 | 75754.61 | 50890.7 | 86841.69 |
| 87120.45 | 84622.37 | 81694.58 | 69192.61 | 70193.62 | 81123.29 |
| 79611.95 | 75809.72 | 82315.62 | 68665.36 | 60335.25 | 77487.43 |
| 85225.34 | 70963.29 | 79615.05 | 56751.98 | 58389.96 | 75626.64 |
| 89066.12 | 83973.56 | 82381.79 | 56437.6 | 72175.29 | 65561.17 |
| 91784.78 | 54564.76 | 86983.09 | 91120.84 | 52168.17 | 98419.54 |
| 55509.62 | 97872.59 | 61554.82 | 55003.83 | 62928.25 | 66427.9 |
| 71510.46 | 69600.9 | 74156.44 | 56629.07 | 60140.21 | 67596.66 |
| 78821.63 | 60772.31 | 76770.78 | 67825.98 | 59710.36 | 74619.38 |
| 76336.21 | 67141.73 | 70211.7 | 69105.15 | 58586.59 | 80425.37 |
| 67588.11 | 64373.09 | 74455.73 | 62407.87 | 50220.27 | 70433.87 |
| 78441.22 | 52582.94 | 66284.01 | 65802.49 | 52634.37 | 70021.34 |
| 107002.9 | 82850.61 | 66342.39 | 35293.87 | 88437.02 | 50114.23 |
| 69531.28 | 49432.05 | 76253.26 | 82494.96 | 33608.91 | 85149.64 |
| 56569.85 | 62300.67 | 63898.23 | 49747.66 | 52226.93 | 50838.26 |
| 71831.89 | 59876.9 | 66502.97 | 58809.15 | 55914.08 | 65172.2 |
| 57916.86 | 56806.86 | 60915.26 | 50168.6 | 46293.86 | 56531.71 |
| 62717.35 | 57669.66 | 60622.82 | 48716.23 | 54197.13 | 54672.67 |
| 59957.98 | 57172.33 | 60538.13 | 39184.2 | 50787.89 | 46305.48 |
| 97089.48 | 62204.55 | 64822.79 | 31834.86 | 75203.15 | 44300.37 |
| 67358.98 | 65215.68 | 58976.41 | 34850.32 | 60203.18 | 43881.82 |
| 47628.44 | 61657.96 | 53110.26 | 43072.27 | 43582.35 | 51083.07 |
| 68110.48 | 57853.62 | 64131.34 | 51680.98 | 48512.85 | 61691.25 |
| 50800.05 | 64639.74 | 53924.37 | 54626.57 | 44900.17 | 59484.04 |
| 61725.08 | 47004.76 | 53418.41 | 42822.45 | 47917.69 | 50736.26 |

| 66017.13 | 36687.63 | 54381.03 | 52297.91 | 40982.01 | 58125.53 |
| --- | --- | --- | --- | --- | --- |
| 54093.64 | 62798.12 | 50823.17 | 31234.48 | 50742.68 | 39534.25 |
| 51293.04 | 36295.15 | 41299.79 | 45244.66 | 28153.07 | 40550.1 |
| 53753.39 | 42541.35 | 47383.21 | 46253.61 | 27718.13 | 48374.66 |
| 41460.23 | 45641.95 | 43501.64 | 27814.37 | 38377.98 | 35943.34 |
| 27197.99 | 45363.42 | 41393.6 | 31997.94 | 36060.89 | 36946.88 |
| 46257.42 | 45948.35 | 44611.42 | 32187.73 | 38555.8 | 42131.54 |
| 25798.92 | 44821.91 | 29135 | 16084.33 | 26353.03 | 35663.6 |
| 51347.97 | 29623.42 | 52726.03 | 51927.32 | 23398.62 | 53307.95 |
| 48211.58 | 33985.37 | 44354.99 | 37934.54 | 33771.67 | 44846.94 |
| 50816.72 | 29666.63 | 43587.03 | 49116.58 | 28083.84 | 51618.21 |
| 28511.65 | 40421.78 | 36331.73 | 27195.05 | 30157.09 | 34760.68 |
| 35301.13 | 38282.73 | 37816.11 | 36379.91 | 28669.46 | 39316.62 |
| 17722.65 | 36392.6 | 32907.12 | 34242.71 | 31936.66 | 30551.44 |
| 43634.46 | 35217.56 | 39576.94 | 29649.9 | 29337.62 | 37963.19 |
| 30845.13 | 37760.35 | 38662.7 | 46193.49 | 23549.97 | 44807.87 |
| 37262.64 | 38563.6 | 37118.13 | 27124.86 | 33949.31 | 33497.42 |
| 44986.23 | 26672.95 | 46133.8 | 51275.2 | 22048.23 | 56514.01 |
| 35101.43 | 22215.08 | 42257.78 | 66211.9 | 16355.45 | 64069.97 |
| 36309.64 | 38150.27 | 35599.47 | 26816.86 | 33647.45 | 32363.85 |
| 64610.41 | 29889.95 | 36777.82 | 24251.5 | 45003.33 | 27452.56 |
| 34611.46 | 35058.07 | 34644.53 | 30424.82 | 22106.08 | 35185.62 |
| 38783.46 | 34143.42 | 38365.03 | 30914.41 | 26723.38 | 37290.46 |
| 18744.75 | 31960.9 | 15684.25 | 4938 | 16103.25 | 31924.41 |
| 69196.74 | 23281.3 | 33966.48 | 14103.76 | 42297.9 | 19845.31 |
| 36852.4 | 30116.19 | 30452.31 | 22530.87 | 30490.14 | 27192.69 |
| 28618.06 | 31685.29 | 28063.64 | 24396.95 | 23730.17 | 27117.6 |
| 32565.31 | 27412.18 | 31346.54 | 33117.03 | 19236.68 | 34167.17 |
| 32334.56 | 29445.79 | 31978.64 | 31077.2 | 22789.97 | 33326.6 |
| 32550.81 | 31810.29 | 31012.79 | 21055.78 | 28646.98 | 26925.26 |
| 38730.01 | 25470.15 | 33702.6 | 25461.75 | 29810.73 | 31560.16 |
| 14559 | 27810.8 | 25267 | 21464.33 | 20624 | 24089.63 |
| 19552.3 | 30958.08 | 28239.46 | 34029.58 | 18580.46 | 32422.04 |
| 30067.23 | 25644.95 | 30815.8 | 28840.7 | 21898.23 | 32000.67 |
| 27924.38 | 25757.65 | 29367.46 | 31063.58 | 24126.14 | 31477.39 |
| 43178.15 | 18855.35 | 30509.81 | 20833.95 | 31030.38 | 25200.18 |
| 32157.31 | 21534.46 | 28674.48 | 25845.44 | 19723.13 | 28751.34 |
| 25718.23 | 23856.95 | 26083.3 | 21217.7 | 20809.13 | 24140.51 |
| 36250.8 | 19548.89 | 25626.19 | 16910 | 32434.14 | 19548.95 |
| 18190.73 | 25520.13 | 25677.3 | 28935.04 | 15223.31 | 29535.67 |
| 25322.14 | 23758.78 | 24724.05 | 29349.45 | 12576.46 | 27944.01 |
| 28403.22 | 14643.99 | 23413.69 | 22377.83 | 16079.18 | 23018.78 |
| 38125.65 | 18632.35 | 27910.56 | 18058.29 | 30850.88 | 24209.02 |
| 24335.4 | 24562.7 | 24026.47 | 20250.29 | 21386.51 | 22746.6 |
| 24378.4 | 23252.79 | 23526.81 | 20280.54 | 20318.64 | 22777.44 |
| 24333.4 | 23790.95 | 23657.47 | 20365.54 | 20848.81 | 22645.6 |
| 24331.4 | 23758.95 | 23622.47 | 20228.54 | 20827.81 | 22640.6 |
| 24208.28 | 7480.46 | 29282.5 | 35157.01 | 5022.37 | 35834.14 |
| 24030.73 | 28612.85 | 23178.63 | 18311.29 | 19765.64 | 23906.18 |
| 24317.98 | 17742.28 | 22066.22 | 27152.12 | 10158.63 | 25513.01 |
| 46969.41 | 16169.22 | 21316.66 | 8844.84 | 27940.67 | 12684.66 |
| 46969.41 | 16167.72 | 21316.66 | 8844.84 | 27940.67 | 12684.66 |
| 23965.98 | 18068.03 | 21882.22 | 16290.87 | 14860.56 | 20569.18 |
| 16845.71 | 11803.82 | 21156.68 | 33302.17 | 6913.01 | 33247.28 |
| 21602.07 | 17128.13 | 18353.57 | 16191.11 | 17378.86 | 17397.41 |
| 20106.32 | 16057.04 | 16269.9 | 8930.78 | 16804.1 | 10415.61 |
| 17842.83 | 17677.54 | 15853.23 | 13334.88 | 18576.26 | 13890.11 |
| 17776.58 | 28556.92 | 18027.56 | 15531.91 | 17232.43 | 20554.37 |

| 23692.08 | 18155.14 | 17548.75 | 5460.99 | 22907.14 | 12185.74 |
| --- | --- | --- | --- | --- | --- |
| 16136.48 | 17290.78 | 15330.72 | 18109.79 | 9980.13 | 16958.18 |
| 12856.34 | 14743.84 | 11182.59 | 16538.84 | 12343.7 | 14469.3 |
| 16758.75 | 19667.24 | 14091.09 | 8728.5 | 18351.78 | 11309.34 |
| 33305 | 12686.8 | 18950 | 6167.5 | 24237 | 10913.5 |
| 17645.48 | 18397.72 | 14288.4 | 11283.15 | 17393.65 | 10140.39 |
| 15413.96 | 7842.42 | 17377.77 | 30230.09 | 4570.34 | 30601.95 |
| 9457.15 | 7318.51 | 16080.82 | 21877.25 | 9819.12 | 15127.72 |
| 26236 | 13822.8 | 13981.5 | 4559 | 20357 | 8886.25 |
| 14430.05 | 5368 | 19570.86 | 21813.31 | 3653.15 | 22703.88 |
| 15578.24 | 15320.07 | 13335.58 | 5469.33 | 14130.28 | 9518.91 |
| 4901.82 | 10885.88 | 10805.14 | 9330.52 | 7698.35 | 8151.02 |
| 16750.31 | 4291.71 | 19538.05 | 26572.21 | 2081.94 | 26536.92 |
| 16715.31 | 4252.71 | 19493.05 | 26528.88 | 2080.94 | 26533.92 |
| 13184.48 | 16970.53 | 11668.97 | 6062.96 | 17311.4 | 8618.26 |
| 13180.48 | 16166.78 | 11264.97 | 6041.21 | 16752.7 | 8512.26 |
| 21034.33 | 6895.56 | 17583.49 | 18610.16 | 9741.69 | 20132.66 |
| 5146.14 | 12859.31 | 11103.06 | 9497.11 | 8651.22 | 6560.27 |
| 12106.31 | 10972.71 | 14750.8 | 19296.38 | 6687.44 | 19484.1 |
| 8374.91 | 11428.25 | 11215.83 | 12375.99 | 6955.91 | 13701.66 |
| 14572.08 | 9271.57 | 10642.75 | 3135 | 11691.45 | 7082.91 |
| 14572.08 | 9271.57 | 10642.75 | 3135 | 11691.45 | 7082.91 |
| 14572.08 | 9271.57 | 10642.75 | 3135 | 11691.45 | 7082.91 |
| 14572.08 | 9271.57 | 10642.75 | 3135 | 11691.45 | 7082.91 |
| 14572.08 | 9271.57 | 10642.75 | 3135 | 11691.45 | 7082.91 |
| 14572.08 | 9271.57 | 10642.75 | 3135 | 11691.45 | 7082.91 |
| 9422.5 | 11488.43 | 10872.59 | 13192.41 | 7326.49 | 12658.25 |
| 9422.5 | 11488.43 | 10872.59 | 13192.41 | 7326.49 | 12658.25 |
| 12184.65 | 5911.71 | 8318.22 | 12987.04 | 10015.7 | 14892.85 |
| 8088.3 | 7255.08 | 11573.27 | 17298.41 | 6787.69 | 14972.71 |
| 14503 | 6814.4 | 10946.25 | 9866.5 | 9543.5 | 11794 |
| 15071 | 1071 | 15384 | 22416.5 | 166 | 24732 |
| 15071 | 1071 | 15384 | 22416.5 | 166 | 24731 |
| 7018.73 | 3157.54 | 7152.3 | 7261.21 | 2618.45 | 5606.68 |
| 14884 | 993 | 15276 | 22396.5 | 150 | 24522 |
| 6874.15 | 5608.71 | 8298.72 | 13371.21 | 5455.9 | 12241.69 |
| 6406.17 | 4923.67 | 8897.25 | 2603.33 | 2309.53 | 3018.58 |
| 1522.42 | 8099.08 | 4618.74 | 4851.34 | 6971.25 | 4395.07 |
| 10320 | 713 | 10261 | 14756 | 66.4 | 16103.82 |
| 9828.16 | 1025 | 8067.83 | 10921.16 | 338.33 | 8996.32 |
| 7975.73 | 696.46 | 7051.14 | 9498.03 | 689.19 | 8805.18 |
| 1699.99 | 6883.83 | 8813.66 | 4182.16 | 2755.16 | 2785.5 |
| 9448 | 1025 | 6427 | 15206.5 | 716 | 16138.33 |
| 5911.67 | 3066.17 | 8550 | 2208.58 | 982.03 | 2098.08 |
| 5911.67 | 3066.17 | 8550 | 2208.58 | 982.03 | 2097.08 |
| 2202.98 | 2447.43 | 4504.04 | 6172.87 | 1701.45 | 4056.42 |
| 8181.5 | 451.5 | 6735.5 | 9042.33 | 178.5 | 8554.83 |
| 8476 | 1738.5 | 5603.5 | 1995 | 7490.5 | 2824.08 |
| 1503.16 | 6139 | 2875.08 | 2335.33 | 2445.33 | 2467 |
| 5891 | 700.5 | 5468.25 | 7814.83 | 66 | 8283.57 |
| 995.67 | 3187.25 | 3372.66 | 2088.25 | 1135.89 | 2265.58 |
| 1684.66 | 6305 | 2776.08 | 2309.83 | 2675.53 | 2842.25 |
| 5597 | 553.5 | 5829.25 | 7605.5 | 55 | 8218.58 |
| 729 | 4279.67 | 1649.67 | 2342.83 | 4476.33 | 1797.17 |
| 1581.16 | 5845 | 2649.08 | 1927.33 | 2369.23 | 2337.66 |
| 1578.16 | 5845 | 2649.08 | 1927.33 | 2369.23 | 2332.66 |
| 5560 | 519.5 | 5221.25 | 7584.5 | 58 | 8244.83 |
| 1374.67 | 1626.17 | 3389.5 | 1479.99 | 606.83 | 1058.58 |

| 5401 | 374 | 5375 | 7607.49 | 72 | 8221.25 |
| --- | --- | --- | --- | --- | --- |
| 1027.16 | 6006 | 2742.83 | 2282.33 | 2419.33 | 2371 |
| 4845 | 248 | 4976 | 7378 | 34 | 7929 |
| 1123.5 | 1340.5 | 2437.5 | 990.49 | 557.5 | 305.15 |
| 611.33 | 5732.17 | 1682 | 1618.33 | 2230.7 | 2144.83 |
| 422.5 | 2398.75 | 820.75 | 154.5 | 987.4 | 2090.2 |
| 1086.46 | 1616.93 | 401.79 | 1246.41 | 1925.27 | 889.01 |
| 674.17 | 1020.67 | 1517 | 692.33 | 595.33 | 933.42 |
| 974.5 | 854.5 | 2176.5 | 1103.82 | 14 | 204.66 |
| 1453.25 | 350.25 | 1483.5 | 266 | 124.25 | 1047 |
| 1453.25 | 350.25 | 1483.5 | 266 | 124.25 | 1047 |
| 2169 | 142 | 890 | 1191.83 | 40 | 429.91 |
| 669 | 726.5 | 1052 | 1490 | 323.5 | 1112 |
| 371 | 248 | 884 | 313.5 | 5 | 101.66 |
| 329.4 | 206.14 | 306.06 | 1809.54 | 878.14 | 322.86 |
| 11 | 2107 | 38 | 60 | 1832 | 370 |
| 473.67 | 823.33 | 947 | 1673.67 | 805.67 | 1274.92 |
| 174 | 72 | 115 | 100 | 4 | 89 |
| 393 | 214 | 293 | 302.33 | 6 | 97.99 |
| 368 | 246 | 313 | 317.5 | 0.012 | 114.66 |
| 513.5 | 359.5 | 452.5 | 514.5 | 14.5 | 103.5 |
| 42.5 | 213.5 | 678.5 | 358.5 | 42.5 | 79 |
| 177 | 150 | 195 | 179 | 11.4 | 101 |
| 372.33 | 152.33 | 392.33 | 449.33 | 161.33 | 207 |
| 214 | 81 | 137 | 66 | 17 | 236 |
| 187 | 78 | 108 | 20 | 16 | 209 |
| 187 | 78 | 108 | 20 | 16 | 209 |
| 187 | 78 | 108 | 20 | 16 | 209 |
| 187 | 78 | 108 | 20 | 16 | 209 |
| 187 | 78 | 108 | 20 | 16 | 209 |
| 187 | 78 | 108 | 20 | 16 | 209 |
| 187 | 78 | 108 | 20 | 16 | 209 |
| 187 | 78 | 108 | 20 | 16 | 209 |
| 187 | 78 | 108 | 20 | 16 | 209 |
| 187 | 78 | 108 | 20 | 16 | 209 |
| 187 | 78 | 108 | 20 | 16 | 209 |
| 187 | 78 | 108 | 20 | 16 | 209 |
| 187 | 78 | 108 | 20 | 16 | 209 |
| 187 | 78 | 108 | 20 | 16 | 209 |
| 983 | 980 | 1176 | 1176 | 0.012 | 89 |
| 120 | 7 | 99 | 1 | 17 | 111 |
| 120 | 7 | 99 | 1 | 17 | 111 |
| 34 | 26 | 11 | 11 | 165 | 79 |
| 100 | 10 | 92 | 160 | 3 | 117 |
| 2 | 15 | 31 | 1 | 19 | 30 |
| 2 | 15 | 31 | 1 | 19 | 30 |
| 2 | 32 | 35 | 137 | 21 | 6 |
| 2 | 32 | 35 | 137 | 21 | 5 |
| 2 | 32 | 35 | 137 | 21 | 5 |
| 2 | 32 | 35 | 137 | 21 | 5 |
| 2 | 32 | 35 | 137 | 21 | 5 |
| 2 | 32 | 35 | 137 | 21 | 5 |
| 2 | 32 | 35 | 137 | 21 | 5 |
| 3 | 12 | 4 | 3 | 4 | 11.58 |
| 35 | 39 | 45 | 43.33 | 1 | 5 |
| 35 | 39 | 45 | 43.33 | 1 | 5 |
| 35 | 39 | 45 | 43.33 | 1 | 5 |
| 35 | 39 | 45 | 43.33 | 1 | 5 |

| 35 | 39 | 45 | 43.33 | 1 | 4 |
| --- | --- | --- | --- | --- | --- |
| 41 | 38 | 47 | 42 | 5 | 5 |
| 0.012 | 0.012 | 0.012 | 0.012 | 0.012 | 0.012 |
| 38 | 35 | 42 | 58 | 0.012 | 18.25 |
| 0.012 | 0.012 | 1 | 2 | 0.012 | 0.012 |
| 35 | 35 | 42 | 42 | 0.012 | 4 |
| 35 | 35 | 42 | 42.33 | 0.012 | 4 |
| 35 | 35 | 42 | 42 | 0.012 | 3 |
| 2 | 1 | 2 | 0.012 | 0.012 | 0.012 |
| 1 | 0.012 | 2 | 1 | 0.012 | 2 |
| 0.012 | 0.012 | 0.012 | 0.012 | 0.012 | 25.98 |
| 0.012 | 0.012 | 0.012 | 0.012 | 0.012 | 0.012 |
| 0.012 | 0.012 | 0.012 | 0.012 | 0.012 | 0.012 |
| 0.012 | 0.012 | 0.012 | 0.012 | 0.012 | 1 |
| 0.012 | 0.012 | 0.012 | 0.012 | 0.012 | 0.012 |
| 0.012 | 0.012 | 0.012 | 0.012 | 0.012 | 1 |
| 0.012 | 0.012 | 0.012 | 0.012 | 0.012 | 0.012 |
| 0.012 | 0.012 | 0.012 | 0.33 | 0.012 | 0.012 |
| 0.012 | 0.012 | 0.012 | 0.012 | 0.012 | 0.012 |
| 0.012 | 0.012 | 0.012 | 0.012 | 0.012 | 0.012 |
| 0.012 | 0.012 | 0.012 | 0.012 | 0.2 | 0.012 |
| 0.012 | 0.012 | 0.012 | 0.012 | 0.012 | 0.012 |
| 0.012 | 0.012 | 0.012 | 0.012 | 0.012 | 0.012 |
| 0.012 | 0.012 | 0.012 | 0.012 | 0.012 | 0.012 |
| 0.012 | 0.012 | 0.012 | 0.012 | 0.012 | 0.012 |
| 0.012 | 0.012 | 0.012 | 0.012 | 0.012 | 0.012 |
| 0.012 | 0.012 | 0.012 | 0.012 | 0.012 | 0.012 |
| 0.012 | 0.012 | 0.012 | 0.012 | 0.012 | 0.012 |
| 0.012 | 0.012 | 0.012 | 0.012 | 0.012 | 0.012 |
| 0.012 | 2 | 0.012 | 0.012 | 1 | 0.012 |
| 0.012 | 0.012 | 0.012 | 0.012 | 0.012 | 0.012 |
| 0.012 | 0.012 | 0.012 | 0.012 | 0.012 | 0.012 |
| 0.012 | 0.012 | 0.012 | 0.012 | 0.012 | 1 |
| 0.012 | 0.012 | 0.012 | 0.012 | 0.012 | 0.012 |
| 0.012 | 0.012 | 0.012 | 0.012 | 0.012 | 0.012 |
| 0.012 | 0.012 | 0.012 | 0.012 | 0.012 | 0.012 |
| 0.012 | 0.012 | 0.012 | 0.012 | 0.012 | 0.012 |
| 0.012 | 0.012 | 0.012 | 0.012 | 0.012 | 0.012 |
| 0.012 | 0.012 | 0.012 | 0.012 | 0.012 | 0.012 |
| 0.012 | 0.012 | 0.012 | 0.012 | 0.012 | 0.012 |
| 0.012 | 0.012 | 0.012 | 0.012 | 0.012 | 0.012 |
| 0.012 | 0.012 | 0.012 | 0.012 | 0.012 | 0.012 |
| 0.012 | 0.012 | 0.012 | 0.012 | 0.012 | 0.012 |
| 0.012 | 0.012 | 0.012 | 0.012 | 0.012 | 0.012 |
| 0.012 | 0.012 | 0.012 | 0.012 | 0.012 | 0.012 |
| 0.012 | 0.012 | 0.012 | 0.012 | 0.012 | 0.012 |
| 0.012 | 0.012 | 0.012 | 0.012 | 0.012 | 0.012 |
| 0.012 | 0.012 | 0.012 | 0.012 | 0.012 | 0.012 |
| 0.012 | 0.012 | 0.012 | 0.012 | 0.012 | 0.012 |
| 0.012 | 0.012 | 0.012 | 0.012 | 0.012 | 0.012 |
| 0.012 | 0.012 | 0.012 | 0.012 | 0.012 | 0.012 |
| 0.012 | 0.012 | 0.012 | 0.012 | 0.012 | 0.012 |
| 0.012 | 0.012 | 0.012 | 0.012 | 0.012 | 0.012 |
| 0.012 | 0.012 | 0.012 | 0.012 | 0.012 | 0.012 |
| 0.012 | 0.012 | 0.012 | 0.012 | 0.012 | 0.012 |
| 0.012 | 0.012 | 0.012 | 0.012 | 0.012 | 0.012 |
| 0.012 | 0.012 | 0.012 | 0.012 | 0.012 | 0.012 |
| 0.012 | 0.012 | 0.012 | 0.012 | 0.012 | 0.012 |

| 0.012 | 0.012 | 0.012 | 0.012 | 0.012 | 0.012 |
| --- | --- | --- | --- | --- | --- |
| 0.012 | 0.012 | 0.012 | 0.012 | 0.012 | 0.012 |
| 0.012 | 0.012 | 0.012 | 0.012 | 0.012 | 0.012 |
| 0.012 | 0.012 | 0.012 | 0.012 | 0.012 | 0.012 |
| 0.012 | 0.012 | 0.012 | 0.012 | 0.012 | 0.012 |
| 0.012 | 0.012 | 0.012 | 0.012 | 0.012 | 0.012 |
| 0.012 | 0.012 | 0.012 | 0.012 | 0.012 | 0.012 |
| 0.012 | 0.012 | 0.012 | 0.012 | 0.012 | 0.012 |
| 0.012 | 0.012 | 0.012 | 0.012 | 0.012 | 0.012 |
| 0.012 | 0.012 | 0.012 | 0.012 | 0.012 | 0.012 |
| 0.012 | 0.012 | 0.012 | 0.012 | 0.012 | 0.012 |
| 0.012 | 0.012 | 0.012 | 0.012 | 0.2 | 0.012 |
| 0.012 | 0.012 | 0.012 | 0.012 | 0.012 | 0.012 |
| 0.012 | 0.012 | 0.012 | 0.012 | 0.012 | 0.012 |
| 0.012 | 0.012 | 0.012 | 0.012 | 0.012 | 0.012 |
| 0.012 | 0.012 | 0.012 | 0.012 | 0.012 | 0.012 |
| 0.012 | 0.012 | 0.012 | 0.012 | 0.012 | 0.012 |
| 0.012 | 0.012 | 0.012 | 0.012 | 0.012 | 0.012 |

| Control_21 | Control_22 | Control_2 | Control_23 | Control_24 | Control_25 |
| --- | --- | --- | --- | --- | --- |
| 4727187.29 | 8649147.02 | 6732500.37 | 7718425.96 | 8396764.02 | 8403096.08 |
| 2295048.28 | 4131789.36 | 3297678.67 | 3618544 | 4070818.68 | 3997044.78 |
| 1009218.47 | 2088966.74 | 1588718.64 | 1763055.88 | 1963326.89 | 1934722.96 |
| 921869.02 | 1711055 | 1317920.2 | 1472010.63 | 1670468.69 | 1714505.78 |
| 685050.71 | 1332607.1 | 977038.3 | 1123239.43 | 1259149.6 | 1216576.22 |
| 707937.81 | 1195545.42 | 964309.91 | 1137448.42 | 1218505.57 | 1215313.18 |
| 496580.14 | 1132614.23 | 999693.35 | 915315.35 | 929084.18 | 906705.36 |
| 469749.09 | 821446 | 701125.06 | 758463.63 | 822826.96 | 805714.71 |
| 347532.29 | 639248.52 | 525502.7 | 589109.56 | 603448.53 | 630887.93 |
| 364921.18 | 573320.84 | 498352.07 | 584868.91 | 612113.53 | 600442.46 |
| 317515.08 | 538445.56 | 436479.07 | 520066.75 | 551943.78 | 561635.09 |
| 355047.33 | 519055.35 | 486139.96 | 535902.47 | 551389.29 | 512694.13 |
| 338073 | 501801.7 | 434075.54 | 516092.16 | 553928.06 | 514753.98 |
| 286466.93 | 498001.93 | 378503.19 | 460867.6 | 496722.07 | 514948.63 |
| 256081.39 | 531572.53 | 355081.76 | 438148.89 | 526480.33 | 475679.23 |
| 306115.81 | 548560.74 | 450858.33 | 429048.1 | 445236.5 | 454005.28 |
| 254472.57 | 513296.07 | 383192.9 | 426894.56 | 460883.32 | 448419.13 |
| 271669.79 | 465913.31 | 386211.62 | 434906.38 | 468995.03 | 463276 |
| 251196.1 | 425579.87 | 332455.74 | 385929.56 | 426049.22 | 409376.45 |
| 302314.23 | 408148.81 | 384400.9 | 441356.53 | 461728.88 | 394383.63 |
| 267007.81 | 406946.04 | 339292.04 | 402320.23 | 419845.33 | 423515.91 |
| 225437.37 | 397190.8 | 278657.6 | 360290.37 | 399466.15 | 402727.27 |
| 223918.96 | 397474.3 | 316522.22 | 370111.2 | 393195.67 | 386703.51 |
| 111121.75 | 346355.3 | 212450.45 | 318856.16 | 334251.64 | 458343.87 |
| 217295.2 | 385287.35 | 264350.73 | 342005.33 | 376916.76 | 376324.7 |
| 215971.2 | 360998.23 | 295277.55 | 305160.79 | 360057.77 | 372885.68 |
| 172492.23 | 353050.56 | 260752.09 | 314682.33 | 343319.79 | 344469.61 |
| 193307.72 | 331035.25 | 271686.85 | 307098.32 | 328873.07 | 324074.85 |
| 183684.87 | 332653.64 | 281998.68 | 293232.55 | 331069.13 | 308334.11 |
| 175974.28 | 321141.44 | 233001.01 | 259580.4 | 316083.99 | 292126.14 |
| 142896.57 | 385500.29 | 260044.62 | 244191.37 | 275074.2 | 268431.93 |
| 172698.37 | 300155.85 | 248578.79 | 263108.46 | 289451.29 | 293012.34 |
| 147430.42 | 295039.89 | 216682.94 | 235268.73 | 281527.82 | 290336.78 |
| 213149.01 | 249459.21 | 268832.23 | 309068.53 | 310586.75 | 287595.5 |
| 134424.23 | 325382.37 | 210280.95 | 214104.29 | 260636.26 | 278411.85 |
| 142844.89 | 269589.09 | 204061.78 | 247485.45 | 274724.21 | 267050.62 |
| 155654.33 | 268829.11 | 240805.84 | 224942.07 | 256471.62 | 277503.73 |
| 100593.99 | 259388.89 | 184601.35 | 211872.83 | 232820.3 | 252192.3 |
| 150674.64 | 269214 | 221899.78 | 245058.23 | 260877.28 | 269409.84 |
| 151124.46 | 258281.36 | 207167.25 | 245117.78 | 263649.53 | 269596.21 |
| 163614.01 | 246875.34 | 195558 | 248751.11 | 264748.66 | 257499.96 |
| 138828.85 | 306879.72 | 226042.6 | 222043.32 | 237739.52 | 217710.45 |
| 151624.77 | 268866.87 | 216752.6 | 229170.93 | 258593.52 | 264787.34 |
| 192948.8 | 242468.07 | 226640.76 | 284077.89 | 283556.57 | 255939.19 |
| 132181.42 | 239741.26 | 169799.98 | 224807.7 | 226323.66 | 269618.05 |
| 120349.17 | 282854.07 | 192045.1 | 214437.97 | 234361.51 | 235648.15 |
| 144273.6 | 299740.16 | 227477.28 | 219545.45 | 236061.44 | 211649.22 |
| 140393.63 | 229380.65 | 193091.19 | 231726.8 | 242433.2 | 253951.26 |
| 138243.79 | 238023.72 | 184074.85 | 221173.01 | 239085.76 | 237933.14 |
| 119526.68 | 277266.2 | 228812.22 | 217584.12 | 241174.88 | 211108.57 |
| 132603.72 | 233078.14 | 173237.81 | 212109.83 | 236674.02 | 211287.11 |
| 124011.49 | 237075.45 | 164330.68 | 195774.81 | 208099.67 | 196936.87 |
| 127125.98 | 211084.29 | 157574.68 | 199892.36 | 200629.27 | 196731.16 |
| 128693.22 | 199495.64 | 168972.3 | 196689.78 | 209751.51 | 198387.51 |
| 114009.26 | 177944.28 | 158084.07 | 182630.99 | 202457.03 | 200603.56 |
| 118631.69 | 186400.08 | 222129.68 | 229729.1 | 189791.68 | 170727.4 |
| 101858.07 | 164443.3 | 148241.9 | 155509.23 | 181227.16 | 194881.85 |

| 84286.93 | 208955.59 | 162152.71 | 135523.79 | 165838.29 | 176181.71 |
| --- | --- | --- | --- | --- | --- |
| 105895.1 | 169156.12 | 143611.88 | 164917.2 | 181479.07 | 183305.38 |
| 107044.16 | 157125.91 | 143354.88 | 166913.55 | 177042.4 | 171418.76 |
| 65761.63 | 220455.96 | 140279.51 | 122925.72 | 130960.28 | 133531.75 |
| 82974.8 | 172482.25 | 108074.09 | 127455.46 | 151934.82 | 158265.61 |
| 83683.41 | 159199.18 | 137547.42 | 150036.51 | 160234.82 | 154729.64 |
| 68543.74 | 204265.96 | 140418.55 | 123937.14 | 131186.09 | 119329.45 |
| 73360.97 | 160981.48 | 106512.41 | 128162.86 | 136835.41 | 142715.69 |
| 82290.39 | 124039.9 | 109182.05 | 130602.82 | 134380.02 | 132119.63 |
| 77947.99 | 143132.85 | 79120.15 | 116731.61 | 139430.72 | 115637.07 |
| 66487.98 | 158730.15 | 122758.27 | 107318.62 | 121359.13 | 107578.16 |
| 61037.75 | 122507.67 | 56268.85 | 90674.45 | 107576.95 | 125669.93 |
| 80862.31 | 130181.4 | 117493.02 | 126770.67 | 129148.84 | 116800.94 |
| 82201.2 | 119826.37 | 101972.23 | 113960.11 | 115494.91 | 121381.15 |
| 65582.73 | 139545.36 | 116344.84 | 101308.22 | 108256.31 | 93290.91 |
| 71357.48 | 109756.63 | 86437.16 | 103947.06 | 113359.85 | 99813.97 |
| 61136.09 | 125088.36 | 128449.41 | 101358.03 | 115556.09 | 90496.32 |
| 55658.38 | 126842.34 | 109701.66 | 88358 | 114377.29 | 116269.57 |
| 61035.38 | 105752.82 | 116286.23 | 96021.95 | 114536.91 | 114743.51 |
| 59223.05 | 113393.4 | 91690.68 | 99518.9 | 110647.29 | 103267.45 |
| 69993.27 | 104178.23 | 89182.39 | 103732.38 | 113994.05 | 105484.88 |
| 55695.8 | 110542.68 | 97722.34 | 97660.5 | 102195.39 | 100369.45 |
| 72481.07 | 111603.29 | 105293.19 | 103148.93 | 107003.01 | 101905.98 |
| 41444.6 | 116399.5 | 123608.09 | 84902.93 | 98107.89 | 95560.28 |
| 40280.15 | 96056.32 | 67307.43 | 81088.07 | 83188.01 | 102241.63 |
| 86845.85 | 75490.68 | 71853.09 | 118796.58 | 117155.87 | 80779.42 |
| 52878.16 | 107689.59 | 82904.88 | 82866.8 | 91182.31 | 87776.46 |
| 64859.08 | 96404.24 | 91761.84 | 89019.55 | 85848.79 | 87915.19 |
| 56004.8 | 91931.38 | 66898.17 | 80431.21 | 89233.81 | 81175.56 |
| 50306.94 | 88588.41 | 72794.45 | 83197.97 | 86514.14 | 86889.64 |
| 48970.93 | 87797.05 | 82390.56 | 75315.75 | 91041.81 | 76180.32 |
| 46403.13 | 70885.92 | 58060.66 | 72130.66 | 83580.78 | 81477.18 |
| 42097.26 | 75211.68 | 59878.32 | 71101.43 | 75930.91 | 80700.51 |
| 31712.15 | 92644.1 | 50057.01 | 62282.79 | 61971.65 | 64563.22 |
| 46508.13 | 81394.62 | 55828.28 | 71039.27 | 77137.58 | 72864.92 |
| 40515.65 | 76088.44 | 58625.51 | 65102.28 | 72085.84 | 65655.72 |
| 45030.6 | 75487.16 | 42625.4 | 63692.35 | 78141.46 | 62508.5 |
| 47861.86 | 62251.16 | 69425.55 | 73064.62 | 84280.21 | 66213.69 |
| 40392.08 | 86306.47 | 49322.2 | 54241.86 | 57613.39 | 53217.07 |
| 44211.02 | 67082.68 | 48597.28 | 66191.18 | 69369.03 | 84027.75 |
| 40129.37 | 64821.72 | 46479.8 | 63057.69 | 67783.81 | 71478.4 |
| 32781.01 | 68562.52 | 36543.98 | 49357.52 | 52075.93 | 65130.27 |
| 37525.14 | 70710.64 | 47085.8 | 57192.2 | 65262.22 | 65533.31 |
| 32297.11 | 73084.34 | 45274.03 | 56524.2 | 60189.32 | 62152.08 |
| 38979.51 | 71147 | 51764.16 | 55072.03 | 57617.97 | 52469.65 |
| 57274.5 | 47574.48 | 46288 | 76745.83 | 76051.77 | 52451.75 |
| 21897.96 | 89535.84 | 46167.95 | 41139.11 | 53043.18 | 45673.4 |
| 35079.83 | 54203.97 | 52912.76 | 60606.82 | 61207.95 | 62560.69 |
| 31528.64 | 58306.12 | 43341.95 | 48143.7 | 53912.22 | 58713.99 |
| 30586.42 | 56582.16 | 42313.58 | 47721.76 | 53950.33 | 57408.5 |
| 40131.06 | 51512.84 | 45924.19 | 56878 | 59122.68 | 57882.36 |
| 30697.36 | 49175.57 | 37960.04 | 56421.38 | 55224.92 | 55878.88 |
| 54957.67 | 40554.49 | 41058.5 | 69792.33 | 67265.12 | 45993 |
| 37307.49 | 40768.98 | 46279.03 | 52198.64 | 62188.26 | 52244.67 |
| 26992.94 | 49324.69 | 45830.31 | 48707.35 | 60957.51 | 53682.9 |
| 26602.41 | 54874.82 | 40037.03 | 43081.98 | 57353.18 | 44804.17 |
| 22621.66 | 54064.14 | 45765.94 | 47620.87 | 62039.31 | 52687.97 |
| 26152.27 | 41482.38 | 29792.42 | 40648.39 | 42338.02 | 46197.3 |

| 29126.48 | 54271.77 | 31558.81 | 40617.12 | 41975.14 | 33729.88 |
| --- | --- | --- | --- | --- | --- |
| 28657.9 | 36287.15 | 43670.69 | 44562.81 | 56789.85 | 48268 |
| 17245.36 | 52735.67 | 74803.54 | 30872.04 | 45317.47 | 37714.96 |
| 20165.36 | 56487.07 | 47315.09 | 36388.72 | 42380.13 | 35334.28 |
| 24684.94 | 34849.87 | 25628.12 | 41403.05 | 42960.77 | 45327.12 |
| 14328.7 | 26279.57 | 19105.65 | 45701.08 | 41630.45 | 47955.04 |
| 17397.15 | 40524.44 | 20669.72 | 36437.99 | 40903.14 | 44502.11 |
| 15458.97 | 33179.53 | 23853.23 | 34127.07 | 43719.23 | 47064.43 |
| 17602.16 | 57474.04 | 36924.96 | 32396.46 | 38319.72 | 30017.72 |
| 18730.54 | 40296.53 | 18721.78 | 33583.48 | 33173.93 | 34247.63 |
| 22157.49 | 55316.97 | 37904.05 | 28824.71 | 32127.97 | 27569.8 |
| 14602.12 | 30495.27 | 21801.12 | 39153.06 | 41874.68 | 41075.55 |
| 19510.4 | 40700.21 | 30444.54 | 31356.45 | 37380.89 | 35973.88 |
| 10428.35 | 27789.25 | 17076.26 | 26897.78 | 22841.62 | 49568.59 |
| 23733.88 | 38314.41 | 25247.7 | 32001.3 | 38954.73 | 31189.54 |
| 9845.25 | 44982.67 | 34937.82 | 27636.79 | 33727.28 | 35601.06 |
| 23193.02 | 33764.71 | 29043.2 | 34582.47 | 37843.45 | 36893.36 |
| 15461.12 | 50508.79 | 21418.68 | 24364.64 | 31278.04 | 27019.78 |
| 17951.51 | 53486.8 | 19158.29 | 24709.75 | 25882.89 | 21663.09 |
| 23165.77 | 31839.05 | 28786.36 | 33277.14 | 37395.12 | 35755.69 |
| 29104.67 | 26595.99 | 23211.67 | 34307.33 | 28914.34 | 26240.25 |
| 15658.31 | 39058.94 | 32241.79 | 27367.71 | 36725.73 | 28303.2 |
| 19699.57 | 34444.61 | 24369.77 | 32103.71 | 37926.25 | 27937.7 |
| 8290.25 | 17517.25 | 6954.61 | 21333.25 | 34307.91 | 34555.75 |
| 36006 | 19906.29 | 20495.66 | 35121.5 | 29041.53 | 17831.08 |
| 19722.37 | 24486.73 | 21146.44 | 28868.81 | 30219.61 | 29079.28 |
| 16578.44 | 31201.28 | 27770.11 | 26193.39 | 30436.69 | 30626.61 |
| 14258.63 | 37986.84 | 30736.78 | 26333.56 | 31969.19 | 21002.69 |
| 15322.95 | 32886.39 | 27533.44 | 24947.14 | 29766.95 | 27339.11 |
| 21895.86 | 23714.21 | 16520.79 | 27190.39 | 30620.27 | 31204.85 |
| 14089.82 | 27152.41 | 13529.17 | 24747.08 | 23901.98 | 27645.69 |
| 1923.8 | 25815.3 | 6115.5 | 24093.5 | 16489.33 | 31403 |
| 9305.24 | 31887.42 | 25048.06 | 22689.78 | 28437.22 | 27844.06 |
| 15452.78 | 29981.45 | 20879.68 | 23164.64 | 26361.04 | 26491.78 |
| 14368.73 | 28432.87 | 21159.7 | 20658.96 | 26330.06 | 27570.13 |
| 21590.21 | 22616.05 | 20310.48 | 27310.31 | 26119.76 | 19696.19 |
| 13954.76 | 31533.48 | 22102.03 | 18486.14 | 21494.02 | 23120.36 |
| 15341.53 | 24445.22 | 20196.28 | 23097.14 | 23467.61 | 24570.78 |
| 25471.92 | 16519.22 | 19441.47 | 29568.87 | 25138.06 | 20332.88 |
| 6988.19 | 27183.02 | 18782.35 | 17613.9 | 21765.78 | 23550.11 |
| 9781.61 | 32809.85 | 26004.35 | 18062.64 | 20735.71 | 18368.52 |
| 15415.08 | 29937.05 | 21546.71 | 23382.63 | 19795.72 | 16108.96 |
| 21490.12 | 19421.4 | 10095.13 | 25418.81 | 23988.85 | 19249.19 |
| 13248.45 | 22156.81 | 17989.52 | 21575.98 | 22909.86 | 24276.61 |
| 13245.62 | 22266.23 | 17980.94 | 21404.31 | 22854.11 | 23005.78 |
| 13243.45 | 22161.31 | 18307.77 | 21464.98 | 22830.61 | 23543.61 |
| 13243.45 | 22156.31 | 17977.77 | 21438.98 | 22806.61 | 23510.61 |
| 11638.78 | 36945.54 | 16614.42 | 14696.44 | 12617.68 | 9847.56 |
| 12835.72 | 21069.65 | 14625.94 | 22565.89 | 26976.36 | 22934.61 |
| 7707.95 | 30712.52 | 21375.35 | 15286.81 | 16287.04 | 14187.69 |
| 22794.33 | 15795.58 | 14765.5 | 22667.5 | 18294.84 | 11111.58 |
| 22794.33 | 15795.58 | 14765.5 | 22666 | 18294.84 | 11111.58 |
| 12068.61 | 22410.54 | 15934.35 | 17806.15 | 20304.2 | 16574.02 |
| 12264.43 | 29945.18 | 12178.64 | 15628.3 | 14159.68 | 10424.79 |
| 10322.01 | 16812.52 | 12724.88 | 13920.06 | 16343.61 | 18497.11 |
| 10792.12 | 12591.79 | 17422.27 | 16416.23 | 16208.44 | 18098.03 |
| 12927.05 | 13550.6 | 15166.02 | 19471.98 | 17570.85 | 19103.04 |
| 9686.61 | 20406.77 | 9546.18 | 16022.66 | 18493.17 | 18354.76 |

| 12984.26 | 8901.19 | 5759.86 | 18003.24 | 18044.41 | 19023.09 |
| --- | --- | --- | --- | --- | --- |
| 7617.61 | 20918.12 | 16042.34 | 13696.81 | 13612.71 | 13846.69 |
| 1368.47 | 14696.42 | 12313.83 | 8056.5 | 11407.56 | 18256.68 |
| 10271.66 | 12446.33 | 9621.51 | 15046.74 | 15930.41 | 19114.26 |
| 16654.5 | 4871.25 | 6695.5 | 19500.5 | 16484 | 11202.5 |
| 17456.53 | 8660.97 | 22304.67 | 14600.83 | 15142.93 | 17079.15 |
| 10967.82 | 26453.76 | 9150.61 | 11767.3 | 11670.18 | 5897.03 |
| 20901.6 | 18678.6 | 19364.39 | 21143.21 | 13542.76 | 10403.04 |
| 12962.92 | 7801 | 6323 | 14950 | 14772.17 | 12166 |
| 4812.6 | 23412.9 | 6162.82 | 9391.04 | 8010.96 | 6570.63 |
| 9222.24 | 9858.66 | 8628.17 | 13446.91 | 13794.75 | 14593.58 |
| 5097.89 | 8947.04 | 7235.39 | 10544.23 | 8299.94 | 12166.86 |
| 6604.1 | 23545.9 | 5447.67 | 5456.57 | 8497.48 | 1942.51 |
| 6600.1 | 23541.9 | 5430.67 | 5455.57 | 8497.48 | 1942.51 |
| 11014.87 | 6507.39 | 8697.36 | 13536.06 | 15583.03 | 12738.52 |
| 11009.87 | 6506.89 | 8685.61 | 13399.06 | 15479.78 | 11972.52 |
| 7791.67 | 15971.33 | 8741.34 | 7536.33 | 10835.5 | 6000.5 |
| 7240.86 | 6743.04 | 15068.36 | 8234.22 | 9948.12 | 14521.02 |
| 9086.28 | 18238.58 | 6310.67 | 6377.57 | 8636.73 | 4769.01 |
| 4422.06 | 13502.07 | 7821.75 | 6737.33 | 9268.5 | 12076.75 |
| 7148.58 | 7757.33 | 3669.17 | 10645.08 | 9322.08 | 10379.75 |
| 7148.58 | 7757.33 | 3669.17 | 10645.08 | 9322.08 | 10379.75 |
| 7148.58 | 7757.33 | 3669.17 | 10645.08 | 9322.08 | 10379.75 |
| 7148.58 | 7757.33 | 3669.17 | 10645.08 | 9322.08 | 10379.75 |
| 7148.58 | 7757.33 | 3669.17 | 10645.08 | 9322.08 | 10379.75 |
| 7148.58 | 7757.33 | 3669.17 | 10645.08 | 9322.08 | 10379.75 |
| 2950.66 | 14192.1 | 10463.42 | 6129.33 | 9056.49 | 11453.51 |
| 2950.66 | 14192.1 | 10463.42 | 6129.33 | 9056.49 | 11453.51 |
| 9395.38 | 10271.93 | 5613.61 | 11750.23 | 9118.7 | 5853.52 |
| 6492.6 | 12772.62 | 7583.84 | 9651.79 | 7628.96 | 7526.38 |
| 5705.5 | 9081 | 2061.5 | 6527 | 8589.25 | 6184.5 |
| 15 | 20617 | 683 | 1313 | 5004 | 630 |
| 15 | 20617 | 683 | 1313 | 5004 | 630 |
| 4301.38 | 11584.9 | 13359.94 | 7944.65 | 6820.79 | 5058.77 |
| 9 | 20533 | 539 | 1200 | 4917 | 528 |
| 3914.71 | 9954.49 | 4344.96 | 5312.23 | 4556.2 | 6564.52 |
| 702.75 | 5916.41 | 5439 | 4958.67 | 7746.41 | 5794.67 |
| 2424.92 | 2508.23 | 2587.59 | 4637.33 | 6563.24 | 10352.26 |
| 38.49 | 13735.82 | 719.57 | 642 | 3172.6 | 132.5 |
| 2101.32 | 10632.82 | 11989.5 | 2057.33 | 4013.67 | 1496.33 |
| 3849.52 | 9499.05 | 7825.44 | 1598.73 | 3028.45 | 1185.1 |
| 4159.32 | 2734.16 | 7352 | 3660.33 | 5335.84 | 6850.16 |
| 14.83 | 14047.83 | 4988.17 | 1077 | 2792 | 1019.5 |
| 646.75 | 5752.66 | 5153.83 | 4271.92 | 5443.41 | 2611.17 |
| 645.75 | 5750.66 | 5153.83 | 4271.92 | 5443.41 | 2611.17 |
| 3146.56 | 4597.63 | 5594.72 | 6376.06 | 3798.76 | 1578.53 |
| 90.34 | 9794.4 | 5333.01 | 1590 | 2674.33 | 341 |
| 5046.58 | 403.08 | 1329.5 | 5110 | 4387 | 1860.5 |
| 2078.83 | 3656.83 | 4629 | 2925.33 | 4563.92 | 4359.83 |
| 28.41 | 8545.99 | 217.67 | 474.5 | 1693.58 | 237 |
| 481.01 | 1470.33 | 1875.65 | 3662.5 | 2884.51 | 2030.84 |
| 2135.33 | 3898.44 | 4631.5 | 3999.83 | 4659.59 | 4872.83 |
| 10.58 | 8485.83 | 126 | 435 | 1631.25 | 287 |
| 265.17 | 871.01 | 893.84 | 2387.33 | 1883 | 6040.67 |
| 2077.66 | 3724.66 | 4456.4 | 2602.83 | 4107.02 | 3518.83 |
| 2076.66 | 3727.66 | 4456.4 | 2602.83 | 4107.02 | 3518.33 |
| 9.58 | 8461.08 | 133 | 490 | 1633.25 | 220.5 |
| 750.76 | 1360.24 | 983.35 | 1491.67 | 1178.5 | 1605.67 |

| 142.85 | 7569.5 | 199.02 | 590.5 | 1809 | 92.5 |
| --- | --- | --- | --- | --- | --- |
| 2079.33 | 2072.83 | 4633.5 | 2723.33 | 4430.34 | 4237.83 |
| 1 | 6688 | 90 | 284 | 1520 | 52 |
| 59.49 | 349.57 | 407.51 | 136 | 220.83 | 989 |
| 8.83 | 1833.33 | 2587 | 2338.83 | 4261.5 | 3760.5 |
| 144.95 | 606.2 | 294.42 | 1035.75 | 1649.25 | 2570 |
| 3594.35 | 2473.33 | 1669.4 | 1176.14 | 2372.71 | 2641.53 |
| 708.1 | 1215.33 | 828.85 | 1433.17 | 1112 | 1373.67 |
| 61.66 | 181.16 | 246.5 | 72 | 68 | 246 |
| 11.5 | 3539.25 | 240.75 | 1082 | 217 | 901.75 |
| 11.5 | 3539.25 | 240.75 | 1082 | 217 | 901.75 |
| 8.33 | 1750.58 | 4864.34 | 622.5 | 1000.83 | 193.5 |
| 427.34 | 1212.65 | 1356 | 1543 | 467.17 | 483.5 |
| 25.66 | 125.16 | 206 | 31 | 44 | 111.5 |
| 3550.86 | 1512.4 | 1783.2 | 792.4 | 705.94 | 512.26 |
| 242 | 47 | 79 | 220 | 189 | 3510 |
| 854.67 | 994.75 | 963.17 | 1532.33 | 887.33 | 711.67 |
| 12 | 32.5 | 78 | 26 | 28 | 19 |
| 19.83 | 115.66 | 113.67 | 27.5 | 47.33 | 31.5 |
| 29.66 | 79.91 | 182 | 18 | 30 | 6.5 |
| 46.51 | 50.75 | 286.35 | 114.5 | 86.5 | 44.5 |
| 4 | 74 | 147 | 250.5 | 282 | 777 |
| 17 | 38 | 47.17 | 10 | 7 | 48 |
| 2065.66 | 191.33 | 1580 | 317 | 115.67 | 274.33 |
| 6 | 156 | 144 | 121 | 103 | 102 |
| 6 | 85 | 144 | 113 | 87 | 102 |
| 6 | 85 | 144 | 113 | 87 | 102 |
| 6 | 85 | 144 | 113 | 87 | 102 |
| 6 | 85 | 144 | 113 | 87 | 102 |
| 6 | 85 | 144 | 113 | 87 | 102 |
| 6 | 85 | 144 | 113 | 87 | 102 |
| 6 | 85 | 144 | 113 | 87 | 102 |
| 6 | 85 | 144 | 113 | 87 | 102 |
| 6 | 85 | 144 | 113 | 87 | 102 |
| 6 | 85 | 144 | 113 | 87 | 102 |
| 6 | 85 | 144 | 113 | 87 | 102 |
| 6 | 85 | 144 | 113 | 87 | 102 |
| 6 | 85 | 144 | 113 | 87 | 102 |
| 6 | 85 | 144 | 113 | 87 | 102 |
| 56 | 11 | 0.012 | 1 | 0.012 | 0.5 |
| 65 | 326 | 7 | 118 | 119 | 14 |
| 65 | 326 | 7 | 118 | 119 | 14 |
| 0.17 | 12 | 7.17 | 197 | 122 | 64 |
| 0.012 | 215 | 9 | 51 | 60 | 23 |
| 0.012 | 8 | 16 | 67 | 124 | 182 |
| 0.012 | 8 | 16 | 67 | 124 | 182 |
| 0.012 | 10 | 330 | 26 | 24 | 33 |
| 0.012 | 5 | 330 | 26 | 24 | 33 |
| 0.012 | 5 | 330 | 26 | 24 | 33 |
| 0.012 | 5 | 330 | 26 | 24 | 33 |
| 0.012 | 5 | 330 | 26 | 24 | 33 |
| 0.012 | 5 | 330 | 26 | 24 | 33 |
| 0.012 | 5 | 330 | 26 | 24 | 33 |
| 19.25 | 10.33 | 4 | 6 | 7.67 | 7 |
| 5 | 7 | 17 | 1 | 0.012 | 0.012 |
| 5 | 7 | 17 | 1 | 0.012 | 0.012 |
| 5 | 7 | 17 | 1 | 0.012 | 0.012 |
| 5 | 7 | 17 | 1 | 0.012 | 0.012 |

| 5 | 7 | 17 | 1 | 0.012 | 0.012 |
| --- | --- | --- | --- | --- | --- |
| 5 | 3 | 0.012 | 13 | 15 | 16 |
| 0.012 | 0.012 | 5 | 3 | 0.012 | 0.012 |
| 2 | 19.25 | 0.012 | 3 | 0.012 | 1 |
| 0.012 | 5 | 1 | 0.012 | 3 | 1 |
| 2.17 | 0.012 | 0.17 | 1 | 0.012 | 0.012 |
| 2 | 1 | 1 | 0.012 | 0.012 | 0.012 |
| 2 | 0.012 | 0.012 | 0.012 | 0.012 | 0.012 |
| 0.012 | 20 | 2 | 2 | 2 | 0.012 |
| 1 | 0.012 | 1 | 6 | 0.012 | 6 |
| 7.98 | 2.23 | 0.012 | 0.012 | 0.012 | 0.012 |
| 0.012 | 0.012 | 0.012 | 14 | 0.012 | 0.012 |
| 0.012 | 5 | 0.012 | 0.012 | 0.012 | 0.012 |
| 0.012 | 5 | 0.012 | 0.012 | 0.012 | 0.012 |
| 0.012 | 1 | 0.012 | 0.012 | 0.012 | 0.012 |
| 1 | 2 | 0.012 | 0.012 | 0.012 | 0.012 |
| 0.012 | 0.012 | 0.012 | 0.012 | 0.012 | 0.012 |
| 0.012 | 2 | 0.012 | 0.012 | 0.012 | 0.012 |
| 0.012 | 0.012 | 0.012 | 0.012 | 0.012 | 0.012 |
| 0.012 | 1 | 0.012 | 0.012 | 0.012 | 0.012 |
| 0.012 | 0.012 | 0.012 | 0.012 | 0.012 | 0.012 |
| 0.012 | 0.012 | 0.012 | 0.012 | 0.012 | 0.012 |
| 0.012 | 0.012 | 0.012 | 0.012 | 0.012 | 0.012 |
| 0.012 | 0.012 | 0.012 | 0.012 | 0.012 | 0.012 |
| 0.012 | 0.012 | 0.012 | 0.012 | 0.012 | 0.012 |
| 0.012 | 0.012 | 0.012 | 0.012 | 0.012 | 0.012 |
| 0.012 | 0.012 | 0.012 | 0.012 | 0.012 | 0.012 |
| 0.012 | 0.012 | 0.012 | 0.012 | 0.012 | 0.012 |
| 1 | 1 | 0.012 | 0.012 | 0.012 | 0.012 |
| 0.012 | 0.012 | 0.012 | 0.012 | 0.012 | 2 |
| 0.012 | 0.012 | 0.012 | 0.012 | 0.012 | 0.012 |
| 1 | 1 | 0.012 | 0.012 | 0.012 | 0.012 |
| 0.012 | 0.012 | 0.17 | 0.012 | 0.012 | 0.012 |
| 0.012 | 0.012 | 0.012 | 0.012 | 0.012 | 0.012 |
| 0.012 | 0.012 | 0.012 | 0.012 | 0.012 | 0.012 |
| 0.012 | 0.012 | 0.012 | 0.012 | 0.012 | 0.012 |
| 0.012 | 2 | 0.012 | 1 | 0.012 | 0.012 |
| 0.012 | 0.012 | 0.012 | 0.012 | 0.012 | 0.012 |
| 0.012 | 0.012 | 0.012 | 0.012 | 0.012 | 0.012 |
| 0.012 | 0.012 | 0.012 | 0.012 | 0.012 | 0.012 |
| 0.012 | 0.012 | 0.012 | 0.012 | 0.012 | 0.012 |
| 0.012 | 0.012 | 0.012 | 0.012 | 0.012 | 0.012 |
| 0.012 | 0.012 | 0.012 | 0.012 | 0.012 | 0.012 |
| 0.012 | 0.012 | 0.012 | 0.012 | 0.012 | 0.012 |
| 0.012 | 0.012 | 0.012 | 0.012 | 0.012 | 0.012 |
| 0.012 | 0.012 | 0.012 | 0.012 | 0.012 | 0.012 |
| 0.012 | 0.012 | 0.012 | 0.012 | 0.012 | 0.012 |
| 0.012 | 0.012 | 0.012 | 0.012 | 0.012 | 0.012 |
| 0.012 | 0.012 | 0.012 | 0.012 | 0.012 | 0.012 |
| 0.012 | 0.012 | 0.012 | 0.012 | 0.012 | 0.012 |
| 0.012 | 0.012 | 0.012 | 0.012 | 0.012 | 0.012 |
| 0.012 | 0.012 | 0.012 | 0.012 | 0.012 | 0.012 |
| 0.012 | 0.012 | 0.012 | 0.012 | 0.012 | 0.012 |
| 0.012 | 0.012 | 0.012 | 0.012 | 0.012 | 0.012 |
| 0.012 | 0.012 | 0.012 | 0.012 | 0.012 | 0.012 |
| 0.012 | 0.012 | 0.012 | 0.012 | 0.012 | 0.012 |
| 0.012 | 0.012 | 0.012 | 0.012 | 0.012 | 0.012 |
| 0.012 | 0.012 | 0.012 | 0.012 | 0.012 | 0.012 |

| 0.012 | 0.012 | 0.012 | 0.012 | 0.012 | 0.012 |
| --- | --- | --- | --- | --- | --- |
| 0.012 | 0.012 | 0.012 | 0.012 | 0.012 | 0.012 |
| 0.012 | 0.012 | 0.012 | 0.012 | 0.012 | 0.012 |
| 0.012 | 0.012 | 0.012 | 0.012 | 0.012 | 0.012 |
| 0.012 | 0.012 | 0.012 | 0.012 | 0.012 | 0.012 |
| 0.012 | 0.012 | 0.012 | 0.012 | 0.012 | 0.012 |
| 0.012 | 0.012 | 0.012 | 0.012 | 0.012 | 0.012 |
| 0.012 | 0.012 | 0.012 | 0.012 | 0.012 | 0.012 |
| 0.012 | 0.012 | 0.012 | 0.012 | 0.012 | 0.012 |
| 0.012 | 0.012 | 0.012 | 0.012 | 0.012 | 0.012 |
| 0.012 | 0.012 | 0.012 | 0.012 | 0.012 | 0.012 |
| 0.012 | 0.012 | 0.012 | 0.012 | 0.012 | 0.012 |
| 0.012 | 0.012 | 0.012 | 0.012 | 0.012 | 0.012 |
| 0.012 | 0.012 | 0.012 | 0.012 | 0.012 | 0.012 |
| 0.012 | 0.012 | 0.012 | 0.012 | 0.012 | 0.012 |
| 0.012 | 0.012 | 0.17 | 0.012 | 0.012 | 0.012 |
| 0.012 | 0.012 | 0.012 | 0.012 | 0.012 | 0.012 |
| 0.012 | 0.012 | 0.012 | 0.012 | 0.012 | 0.012 |

| Control_3 | Control_26 | Control_27 | Control_28 | Control_4 |
| --- | --- | --- | --- | --- |
| 8896702.78 | 8134673.84 | 8489654.74 | 7740985.96 | 8411079.92 |
| 4291330.42 | 3883765.84 | 4020966.09 | 3679413.11 | 4079399.98 |
| 2161386.09 | 1831154.17 | 1984652.98 | 1746344.4 | 1945001.16 |
| 1781073.3 | 1545695.86 | 1682359.46 | 1502302.82 | 1689682.28 |
| 1349215.15 | 1210797.85 | 1248561.34 | 1123367 | 1245012.35 |
| 1243466.99 | 1169596.29 | 1204179.93 | 1111997.83 | 1236389.21 |
| 1230820.16 | 725725.31 | 987981.84 | 775609.11 | 801592.19 |
| 870678.86 | 778768.44 | 798106.92 | 744779.82 | 822867.91 |
| 618346.46 | 525435.46 | 620856.12 | 525605.95 | 606116.47 |
| 615618.56 | 601846.6 | 603932.9 | 574452.57 | 612165.31 |
| 564325.5 | 523676.06 | 548919.06 | 502171.08 | 559130.55 |
| 534730.57 | 522924.91 | 514797.26 | 509292.98 | 523636.67 |
| 515423.31 | 528306.73 | 505503.48 | 500113.27 | 542407.78 |
| 517111.38 | 511985.82 | 521154.88 | 477414.77 | 507175.75 |
| 509092.29 | 506741.96 | 467616.02 | 462850.61 | 520428.76 |
| 579967.07 | 420670.89 | 470862.66 | 409464.44 | 421637.98 |
| 531250.69 | 443404.03 | 473567.38 | 415283.68 | 448169.66 |
| 493723.1 | 449799.63 | 466436.23 | 423190.81 | 467863.19 |
| 427322.27 | 416886.92 | 418667.13 | 387009.2 | 426463 |
| 400976.31 | 411125.43 | 399107.55 | 418045.13 | 413971.33 |
| 427182.97 | 410778.5 | 406926.2 | 396330.44 | 418631.53 |
| 419655.35 | 422602.64 | 406094.12 | 379273.1 | 413845.94 |
| 413009.86 | 382161.73 | 396068.2 | 358005.73 | 396643.29 |
| 333796.39 | 305235.21 | 431621.59 | 313310.44 | 391995.45 |
| 367634.14 | 382150.25 | 378933.14 | 353295.9 | 382228.75 |
| 370333.77 | 336324.79 | 356502.48 | 325072.28 | 376109.29 |
| 370805.53 | 303999.33 | 340944.32 | 304189.16 | 338235.01 |
| 353390.86 | 311787.49 | 330003.47 | 297069.12 | 330251.2 |
| 351786.29 | 310978.71 | 320246.94 | 292157.84 | 317583.76 |
| 330832.2 | 329694 | 303197.87 | 283026.55 | 319144.59 |
| 361768.55 | 268595.89 | 319628.21 | 249568.94 | 261166.39 |
| 319488.37 | 270986.23 | 300276.6 | 260690.54 | 291624.73 |
| 323656.51 | 292907.99 | 283771.06 | 261660.81 | 297933.22 |
| 301977.66 | 298230.99 | 273586.75 | 301969.38 | 298916.68 |
| 320302.4 | 234223.21 | 288267.45 | 226603.07 | 263341.82 |
| 285408.64 | 260115.22 | 274211.65 | 245256.5 | 275522.79 |
| 294123.48 | 263107.73 | 270844.02 | 242379.74 | 274494.43 |
| 290457.65 | 262081.35 | 292636.6 | 239511.8 | 230916.65 |
| 286417.14 | 249794.89 | 269340.65 | 239511.99 | 266671.36 |
| 275570.55 | 249170.48 | 262537.92 | 250330.89 | 261125.84 |
| 252557.71 | 275064.31 | 256798.57 | 250948.18 | 270454.31 |
| 321957.29 | 221431.35 | 278234.17 | 221349.42 | 217419.99 |
| 283342.38 | 247344.33 | 260284.79 | 237383.81 | 269891.04 |
| 229835.93 | 260716.72 | 245378 | 266146.02 | 268237.83 |
| 240428.84 | 237061.23 | 250611.87 | 225726.29 | 247035.47 |
| 299468.38 | 223061.52 | 258670.09 | 209963.71 | 247388.05 |
| 320738.77 | 222327.38 | 273259.98 | 222225.34 | 212416.01 |
| 244293.81 | 233135.44 | 237467.08 | 226577.09 | 253368.14 |
| 241991.8 | 230652.75 | 241072.12 | 219292.19 | 239600.85 |
| 271697.97 | 217809.72 | 229054.81 | 199987.03 | 223266.12 |
| 223442.22 | 236486.78 | 212857.2 | 211562.59 | 230470.51 |
| 252559.93 | 219731.69 | 218370.05 | 205332.96 | 192847.8 |
| 204739.06 | 207725.57 | 193589.29 | 179621.6 | 193143.1 |
| 218723.22 | 212470.08 | 195109.91 | 189040.87 | 204279.23 |
| 188479.65 | 189247.82 | 187639.84 | 179975.95 | 209822.25 |
| 295954.1 | 134201.09 | 164561.9 | 174558.5 | 153107.28 |
| 180153.16 | 188510.77 | 186141.86 | 173077.43 | 197904.49 |

| 207166.96 | 132282.4 | 179760.32 | 135720.38 | 168849.47 |
| --- | --- | --- | --- | --- |
| 181743.39 | 174234.18 | 179382.73 | 166896.64 | 184622.39 |
| 166167.55 | 173283.72 | 166472.82 | 163644.73 | 172344.97 |
| 233485.66 | 124222.36 | 182756.44 | 127416.29 | 120728.88 |
| 170530.09 | 155156.54 | 159612.99 | 139246.73 | 156029.26 |
| 175910.32 | 147272.95 | 170863.65 | 139935.43 | 153985.06 |
| 193918.9 | 127685.67 | 161648.47 | 116878.26 | 115978.27 |
| 161891.57 | 140768.71 | 149862.52 | 133292.73 | 134136.97 |
| 128293.97 | 126068.37 | 129526.19 | 120949.65 | 134243.6 |
| 108550.48 | 135756.13 | 122266.43 | 121656.49 | 130725.55 |
| 169682.56 | 115345.05 | 119014.38 | 103960.58 | 116650.31 |
| 118758.02 | 124685.19 | 119907.57 | 106344.47 | 118986.6 |
| 140345.28 | 120377.36 | 114664.01 | 115825.09 | 118845.27 |
| 127383.05 | 107251.56 | 122894.03 | 106098.4 | 118245.73 |
| 167271.32 | 110294.35 | 119158.84 | 103063.18 | 85957.68 |
| 116436.76 | 126618.36 | 117644.54 | 106227.32 | 108245.59 |
| 132409.47 | 98502.07 | 104797.03 | 90955.76 | 95769.16 |
| 136549.48 | 94268.08 | 112998.01 | 89252.56 | 118009.14 |
| 150325.17 | 119929.54 | 114847.75 | 111666.9 | 122180.64 |
| 120226.99 | 106093.37 | 112929.15 | 100273.27 | 106090.22 |
| 109084.56 | 113811.35 | 107872.07 | 103947.44 | 114206.81 |
| 128902.16 | 100748.96 | 107906.51 | 95755.13 | 101441.27 |
| 112347.21 | 91146.98 | 109390.22 | 91253.75 | 100694.76 |
| 135527.49 | 66731.19 | 99497.29 | 67648.27 | 92816.61 |
| 101639.42 | 78825.16 | 99586.4 | 76675.17 | 100910.69 |
| 49492.8 | 140388.26 | 93425.19 | 121879.59 | 120535.93 |
| 106341.07 | 84304.64 | 95110.03 | 80111.69 | 87985.33 |
| 102019.96 | 72331.46 | 93615.22 | 75253.92 | 84884.91 |
| 81391.32 | 91452.14 | 85382.73 | 82732.71 | 86126.15 |
| 92200.73 | 79482.72 | 86157.69 | 78057.77 | 84940.68 |
| 89332.08 | 81829.32 | 82095.74 | 77142.88 | 86849.84 |
| 64900.74 | 79967.69 | 74627.62 | 73522.37 | 88123.65 |
| 76348.66 | 71204.63 | 79418.87 | 68365.87 | 82222.49 |
| 73361.91 | 57955.39 | 73478.95 | 52864.65 | 65846.74 |
| 71595.32 | 76463.34 | 80884.68 | 75713.79 | 72054.25 |
| 65376.66 | 66324.8 | 64336.02 | 62715.32 | 72183.3 |
| 52131.5 | 74571.67 | 70638.7 | 65172.11 | 74644.82 |
| 63784.74 | 80639.63 | 63306.47 | 71557.91 | 82796.76 |
| 79249.08 | 67138.66 | 65914.48 | 58964.71 | 51954.74 |
| 64146.74 | 57364.86 | 69477.82 | 73341.66 | 68420.92 |
| 62095.16 | 64420.85 | 71987.58 | 61559.37 | 66816.89 |
| 65311.93 | 62931.92 | 71597.79 | 58744.34 | 56915.61 |
| 66153.58 | 66098.59 | 65600.8 | 60511.72 | 66302.56 |
| 65682.91 | 56350.09 | 65755.85 | 53869.95 | 60084.48 |
| 83019.57 | 57969.51 | 64289.84 | 56907.61 | 53159.53 |
| 30624.48 | 92526.76 | 57677.63 | 82257.59 | 81967.69 |
| 80058.75 | 48266.34 | 57677.88 | 40153.97 | 49865.44 |
| 66214.31 | 52503.18 | 57465.88 | 53690.12 | 60218.68 |
| 63552.76 | 59741.02 | 60642.01 | 55413.61 | 58341.48 |
| 55850 | 51246.86 | 61311.72 | 48121.12 | 53192.91 |
| 55467.74 | 58766.72 | 55242.09 | 55004.12 | 59218.67 |
| 50110.16 | 53329.94 | 58768.14 | 50357.63 | 54061.94 |
| 27994.15 | 87947.11 | 52860.5 | 69938.41 | 70369.47 |
| 38755.83 | 61015.83 | 47456.15 | 56108.83 | 65021 |
| 45407.99 | 49860.48 | 46745.64 | 46989.23 | 61420.09 |
| 47465.67 | 55373 | 44497.16 | 48905.34 | 55927.94 |
| 51682.99 | 51866.92 | 42796.23 | 48730.98 | 63601.66 |
| 43017.26 | 50065.98 | 46492.27 | 45935.37 | 44886.63 |

| 50173.49 | 49339.17 | 44844.98 | 43710.06 | 35763.14 |
| --- | --- | --- | --- | --- |
| 35090.17 | 51387.17 | 39805.32 | 47672.51 | 60656.94 |
| 80271.08 | 31527.03 | 42689.81 | 28133.32 | 42733.91 |
| 56068.25 | 33779.44 | 45310.47 | 32707.47 | 37098.14 |
| 31610.83 | 40102.78 | 47361.31 | 39307.64 | 40121.23 |
| 45938.58 | 38203.58 | 43787.11 | 42661.02 | 41431.02 |
| 28005.26 | 39883.48 | 39870.76 | 36039.05 | 47301.25 |
| 22711.83 | 26177.77 | 41872.11 | 30464.23 | 38507.48 |
| 61308.24 | 32914.59 | 41499.23 | 29514.06 | 31108.59 |
| 33599 | 37973.04 | 38704.74 | 34908.06 | 29363.4 |
| 55949.99 | 29543.09 | 41135.06 | 32175.31 | 24746.39 |
| 37344.33 | 32962.38 | 40732.47 | 33276.4 | 41505.66 |
| 41916.16 | 32692.76 | 39382.31 | 30888.13 | 39520.32 |
| 38175.34 | 24978.5 | 40355.03 | 28847.5 | 34072.27 |
| 30472 | 37229.42 | 34675.64 | 32748.14 | 37297.16 |
| 52861.08 | 27164.64 | 37452.7 | 26837.47 | 36344.94 |
| 28479.33 | 36642.54 | 35297.23 | 33404.4 | 41201.31 |
| 44194.33 | 31448.07 | 31997.27 | 26749.73 | 27270.66 |
| 46337.56 | 28668.87 | 30985.42 | 25879.02 | 16685.7 |
| 28474.33 | 36476.55 | 34018.23 | 33157.41 | 40483.98 |
| 29336.83 | 45378.24 | 35788.16 | 39921.41 | 32251.53 |
| 31907.74 | 28355.02 | 32328.65 | 26615.66 | 32315.16 |
| 25581.99 | 36969.72 | 28889.94 | 30325.91 | 33783.76 |
| 18839.5 | 17842 | 26397.25 | 19096.5 | 32357.4 |
| 16404.91 | 47664.44 | 29471.77 | 38292.83 | 29785.13 |
| 24721.33 | 32185.71 | 27433.07 | 29600.57 | 30511.23 |
| 30070.08 | 25201.38 | 31228.4 | 24702.99 | 30284.08 |
| 42450.66 | 26480.21 | 26860.33 | 23008.57 | 30470.89 |
| 33133.33 | 26485.38 | 27118.4 | 24499.24 | 28917.33 |
| 16477.91 | 33140.68 | 27913.2 | 27420.82 | 29696.59 |
| 26003.76 | 31214.32 | 31356.99 | 28309.89 | 25842.22 |
| 18396 | 18367.67 | 39083.5 | 19927 | 25006.3 |
| 38115.16 | 21219.08 | 26495.14 | 22080.3 | 28337.66 |
| 30487.33 | 25043.07 | 25754.27 | 23035.73 | 26826.66 |
| 31969.33 | 26351.86 | 26411.48 | 23098.9 | 28297.66 |
| 24119.91 | 34328.59 | 22593.3 | 28872.41 | 24078.73 |
| 27888.42 | 22097.29 | 28372.16 | 19422.24 | 20278.5 |
| 23838.33 | 22325.87 | 24020.4 | 21137.73 | 24298.16 |
| 23655.07 | 34075.16 | 20537.32 | 30787.64 | 24542.82 |
| 28967.83 | 18115.55 | 23681.15 | 17812.57 | 23956.51 |
| 31741.08 | 15557.75 | 24292.69 | 16043.82 | 17744.41 |
| 28320.57 | 18975.76 | 25936.07 | 18625.23 | 14116.17 |
| 16638.41 | 34168.59 | 22828.46 | 28733.91 | 23305.73 |
| 23035.08 | 21892.88 | 22797.32 | 21511.15 | 23538.66 |
| 22991.83 | 21848.71 | 22627.57 | 20848.57 | 23245.83 |
| 23040.83 | 21844.88 | 22643.57 | 21139.9 | 23357.66 |
| 23034.83 | 21838.88 | 22642.57 | 21135.9 | 23354.66 |
| 33915.24 | 12460.22 | 16963.15 | 10965.39 | 9013.45 |
| 14476.41 | 22340.37 | 18644.91 | 22893.48 | 25146.64 |
| 30323.58 | 13088.58 | 22664.36 | 13552.65 | 12463.41 |
| 13145.83 | 28936.75 | 21927.66 | 25168.42 | 18449.3 |
| 13145.83 | 28936.75 | 21927.66 | 25168.42 | 18449.3 |
| 19230.5 | 18916.71 | 19050.07 | 16995.57 | 19525.58 |
| 21944.9 | 13535.63 | 15675.27 | 13434.41 | 7226.34 |
| 19825.75 | 18545.62 | 18917.08 | 17532.9 | 15903.5 |
| 17423.92 | 16696.12 | 19054.58 | 15078.07 | 17547.75 |
| 19443.66 | 17576.71 | 19116.74 | 18817.73 | 15790.41 |
| 13800.25 | 17891.97 | 18894.08 | 21190.39 | 18138.34 |

| 6476.5 | 22233.32 | 15560.09 | 19917.66 | 19719.8 |
| --- | --- | --- | --- | --- |
| 21335.08 | 10847.08 | 19336.52 | 12338.15 | 11851.91 |
| 28872.84 | 11261.16 | 20345.66 | 10202.48 | 19249.5 |
| 11024.5 | 16492 | 18791.74 | 15824.66 | 20058.4 |
| 4933 | 28430 | 11412 | 20838 | 17137.8 |
| 18400 | 17021.35 | 13952.82 | 15589.85 | 17629.9 |
| 17280.65 | 12204.31 | 12760.01 | 10895.83 | 5100.19 |
| 27880.8 | 11794.65 | 11538.27 | 14658.18 | 7623.06 |
| 6114 | 20728 | 14929 | 17530 | 14568.8 |
| 17890.08 | 8516.96 | 12161.3 | 7670.97 | 4265.77 |
| 4581 | 13611.67 | 12407.08 | 12702.67 | 14544.9 |
| 14161.33 | 6804.46 | 10561.84 | 8814.98 | 9904.51 |
| 15767.99 | 9383.93 | 9265.02 | 6589.59 | 2681.93 |
| 15758.99 | 9383.93 | 9265.02 | 6589.59 | 2681.93 |
| 3888.91 | 15081.38 | 10613.24 | 15861.49 | 15049.66 |
| 3888.66 | 15027.38 | 10458.49 | 15486.24 | 14865.66 |
| 15476.5 | 14656.66 | 8115.5 | 10679.16 | 7852.73 |
| 15325.75 | 7607.71 | 8116.74 | 8389.49 | 14286.6 |
| 12296.49 | 7970.93 | 10452.52 | 10369.09 | 4178.43 |
| 12626.67 | 8046.67 | 12462.99 | 7038.33 | 12569.75 |
| 3157.5 | 11136.5 | 10777.75 | 10207.5 | 9260.9 |
| 3157.5 | 11136.5 | 10777.75 | 10207.5 | 9260.9 |
| 3157.5 | 11136.5 | 10777.75 | 10207.5 | 9260.9 |
| 3157.5 | 11136.5 | 10777.75 | 10207.5 | 9260.9 |
| 3157.5 | 11136.5 | 10777.75 | 10207.5 | 9260.9 |
| 3157.5 | 11136.5 | 10777.75 | 10207.5 | 9260.9 |
| 14430.92 | 7510.33 | 12419.08 | 7404.08 | 9873.67 |
| 14430.92 | 7510.33 | 12419.08 | 7404.08 | 9873.67 |
| 7666.16 | 13941.88 | 8138.83 | 11414.74 | 7919.76 |
| 16839.99 | 8794.55 | 9759.96 | 9066.47 | 5946.35 |
| 7689.5 | 12318 | 8886.5 | 9655 | 7410.4 |
| 13797 | 6420 | 6392 | 3774 | 479 |
| 13797 | 6420 | 6392 | 3774 | 479 |
| 14151.66 | 3578.38 | 9105.74 | 4451.91 | 4028.26 |
| 13707 | 6405 | 6243 | 3714 | 444 |
| 12072.66 | 6263.88 | 7169.66 | 6563.74 | 4656.76 |
| 9087 | 3366 | 7016.5 | 3478.5 | 4613 |
| 5692.33 | 5114.49 | 5899.82 | 6003.48 | 9666.91 |
| 9171 | 4254.2 | 4186.37 | 2272 | 399 |
| 13831.5 | 2646.67 | 4123.83 | 1463.67 | 1314 |
| 10210 | 3435.46 | 5026.33 | 1750.66 | 1108.93 |
| 2297 | 2881.84 | 1836.66 | 2790.84 | 6756 |
| 10284 | 4086 | 5908 | 2662 | 689 |
| 9055.75 | 2203 | 6227 | 2194.75 | 2398.75 |
| 9054.75 | 2203 | 6227 | 2194.75 | 2398.75 |
| 9091.33 | 3143.66 | 3352.3 | 3548.89 | 2033.32 |
| 8988.5 | 2241.5 | 3327.84 | 1214.5 | 611.5 |
| 1405.5 | 8990 | 2538.5 | 6133.5 | 5228.5 |
| 2338.5 | 2548.17 | 3271 | 2565.67 | 5316 |
| 5897 | 2206.5 | 4816.67 | 1264 | 204 |
| 4061.25 | 1685.69 | 2274.45 | 2595.74 | 1355.83 |
| 2438 | 2639.67 | 3460.83 | 2646.17 | 5621 |
| 5796 | 2174.5 | 4740 | 1260 | 242 |
| 2928 | 2591.33 | 4893.17 | 3050.67 | 3855.67 |
| 2719.5 | 2450.87 | 4180.53 | 2389.17 | 5018 |
| 2719.5 | 2450.87 | 4179.53 | 2388.17 | 5018 |
| 5804 | 2179.5 | 4756 | 1266 | 164 |
| 524 | 474 | 1202.01 | 466 | 1050 |

4637 2262.5 2351.35 1258.5 474

1405.5 2463.67 1595.83 2503.67 5254

4497 2099 1966 1131 127

257.5 91.5 295.17 370.5 485

563 2154.5 1492 2448.5 4914

144.5 276 1258.75 1078 519.75

1198.33 2529.2 3303.59 1786.34 1963.2

467.5 449.5 1123.51 459.5 833

86.5 27 127.5 11.5 244.5

1285 117 3820.5 180 649.25 1285 117 3820.5 180 649.25 4199 78 1028 123 287.5

3820 463.5 309.17 986 521.5

95 24 53 1 146

610.66 1226.77 1029.46 1371 812.6

49 1114 933 1047 442

620.33 323.67 400.33 682.33 297.33

47 24 173 4 41

111 20 123.67 4 50

87 12 52 0.012 51

78.5 26.01 83.01 8.5 58

14.5 23.5 58 106.5 337

20 0.012 48 18 21

825 292.67 103.33 37.67 249

93 24 152 60 36

90 15 149 60 35

90 15 149 60 35

90 15 149 60 35

90 15 149 60 35

90 15 149 60 35

90 15 149 60 35

90 15 149 60 35

90 15 149 60 35

90 15 149 60 35

90 15 149 60 35

90 15 149 60 35

90 15 149 60 35

90 15 149 60 35

90 15 149 60 35

0.012 0.012 0.012 0.012 0.012

0.012 68 170 63 162

0.012 68 170 63 162

4 23 106.17 11 54

37 27 95 46 3

0.012 45 12 61 38

0.012 45 12 61 38

6 6 1 4 3

6 6 1 4 3

6 6 1 4 3

6 6 1 4 3

6 6 1 4 3

6 6 1 4 3

6 6 1 4 3

0.012 6 10 6 6

10 0.012 0.012 0.012 0.012

10 0.012 0.012 0.012 0.012

10 0.012 0.012 0.012 0.012

10 0.012 0.012 0.012 0.012

| 10 | 0.012 | 0.012 | 0.012 | 0.012 |
| --- | --- | --- | --- | --- |
| 2 | 12 | 1 | 1 | 3 |
| 0.012 | 0.012 | 0.012 | 0.012 | 0.012 |
| 0.012 | 0.012 | 0.012 | 0.012 | 0.012 |
| 18 | 0.012 | 0.012 | 0.012 | 1 |
| 0.012 | 0.012 | 0.17 | 0.012 | 1 |
| 0.012 | 0.012 | 0.012 | 0.012 | 0.012 |
| 0.012 | 0.012 | 0.012 | 0.012 | 0.012 |
| 3 | 10 | 0.012 | 0.012 | 2 |
| 0.012 | 1 | 0.012 | 0.012 | 0.012 |
| 0.012 | 0.012 | 0.012 | 0.012 | 0.012 |
| 0.012 | 0.012 | 0.012 | 0.012 | 12 |
| 1 | 0.012 | 1 | 0.012 | 0.012 |
| 0.012 | 0.012 | 0.012 | 0.012 | 0.012 |
| 0.012 | 0.012 | 0.012 | 0.012 | 0.012 |
| 1 | 0.012 | 0.012 | 0.012 | 0.012 |
| 0.012 | 0.012 | 0.012 | 0.012 | 0.012 |
| 0.012 | 0.012 | 0.012 | 0.012 | 0.012 |
| 0.012 | 0.012 | 0.012 | 0.012 | 0.012 |
| 0.012 | 0.012 | 0.012 | 0.012 | 0.012 |
| 0.012 | 0.012 | 0.012 | 0.012 | 0.012 |
| 0.012 | 0.012 | 0.012 | 0.012 | 0.012 |
| 0.012 | 0.012 | 0.012 | 0.012 | 0.012 |
| 0.012 | 0.012 | 0.012 | 0.012 | 0.012 |
| 0.012 | 0.012 | 0.012 | 0.012 | 0.012 |
| 0.012 | 0.012 | 0.012 | 0.012 | 0.012 |
| 0.012 | 0.012 | 0.012 | 0.012 | 0.012 |
| 0.012 | 0.012 | 0.012 | 0.012 | 0.012 |
| 0.012 | 0.012 | 0.012 | 0.012 | 0.012 |
| 0.012 | 0.012 | 0.012 | 1 | 0.012 |
| 0.012 | 0.012 | 0.012 | 0.012 | 0.012 |
| 0.012 | 0.012 | 0.012 | 0.012 | 0.012 |
| 0.012 | 0.012 | 0.012 | 0.012 | 0.012 |
| 0.012 | 0.012 | 0.012 | 0.012 | 0.012 |
| 0.012 | 0.012 | 0.012 | 0.012 | 0.012 |
| 0.012 | 0.012 | 0.012 | 0.012 | 0.012 |
| 0.012 | 0.012 | 0.012 | 0.012 | 0.012 |
| 0.012 | 0.012 | 0.012 | 0.012 | 0.012 |
| 0.012 | 0.012 | 0.012 | 0.012 | 0.012 |
| 0.012 | 0.012 | 0.012 | 0.012 | 0.012 |
| 0.012 | 0.012 | 0.012 | 0.012 | 0.012 |
| 0.012 | 0.012 | 0.012 | 0.012 | 0.012 |
| 0.012 | 0.012 | 0.012 | 0.012 | 0.012 |
| 0.012 | 0.012 | 0.012 | 0.012 | 0.012 |
| 0.012 | 0.012 | 0.012 | 0.012 | 0.012 |
| 0.012 | 0.012 | 0.012 | 0.012 | 0.012 |
| 0.012 | 0.012 | 0.012 | 0.012 | 0.012 |
| 0.012 | 0.012 | 0.012 | 0.012 | 0.012 |
| 0.012 | 0.012 | 0.012 | 0.012 | 0.012 |
| 0.012 | 0.012 | 0.012 | 0.012 | 0.012 |
| 0.012 | 0.012 | 0.012 | 0.012 | 0.012 |
| 0.012 | 0.012 | 0.012 | 0.012 | 0.012 |
| 0.012 | 0.012 | 0.012 | 0.012 | 0.012 |
| 0.012 | 0.012 | 0.012 | 0.012 | 0.012 |
| 0.012 | 0.012 | 0.012 | 0.012 | 0.012 |
| 0.012 | 0.012 | 0.012 | 0.012 | 0.012 |
| 0.012 | 0.012 | 0.012 | 0.012 | 0.012 |
| 0.012 | 0.012 | 0.012 | 0.012 | 0.012 |

| 0.012 | 0.012 | 0.012 | 0.012 | 0.012 |
| --- | --- | --- | --- | --- |
| 0.012 | 0.012 | 0.012 | 0.012 | 0.012 |
| 0.012 | 0.012 | 0.012 | 0.012 | 0.012 |
| 0.012 | 0.012 | 0.012 | 0.012 | 0.012 |
| 0.012 | 0.012 | 0.012 | 0.012 | 0.012 |
| 0.012 | 0.012 | 0.012 | 0.012 | 0.012 |
| 0.012 | 0.012 | 0.012 | 0.012 | 0.012 |
| 0.012 | 0.012 | 0.012 | 0.012 | 0.012 |
| 0.012 | 0.012 | 0.012 | 0.012 | 0.012 |
| 0.012 | 0.012 | 0.012 | 0.012 | 0.012 |
| 0.012 | 0.012 | 0.012 | 0.012 | 0.012 |
| 0.012 | 0.012 | 0.012 | 0.012 | 0.012 |
| 0.012 | 0.012 | 0.012 | 0.012 | 0.012 |
| 0.012 | 0.012 | 0.012 | 0.012 | 0.012 |
| 0.012 | 0.012 | 0.012 | 0.012 | 0.012 |
| 0.012 | 0.012 | 0.012 | 0.012 | 0.012 |
| 0.012 | 0.012 | 0.012 | 0.012 | 0.012 |
| 0.012 | 0.012 | 0.012 | 0.012 | 0.012 |
